# Properties of LINE-1 proteins and repeat element expression in the context of amyotrophic lateral sclerosis

**DOI:** 10.1186/s13100-018-0138-z

**Published:** 2018-12-15

**Authors:** Gavin C. Pereira, Laura Sanchez, Paul M. Schaughency, Alejandro Rubio-Roldán, Jungbin A. Choi, Evarist Planet, Ranjan Batra, Priscilla Turelli, Didier Trono, Lyle W. Ostrow, John Ravits, Haig H. Kazazian, Sarah J. Wheelan, Sara R. Heras, Jens Mayer, Jose Luis García-Pérez, John L. Goodier

**Affiliations:** 1 McKusick-Nathans Institute of Genetic Medicine, Johns Hopkins University School of Medicine, Baltimore, Maryland USA; 2 GENYO. Centre for Genomics and Oncological Research: Pfizer, University of Granada, Andalusian Regional Government, Granada, Spain; 3 Oncology Center-Cancer Biology, Johns Hopkins University School of Medicine, Baltimore, Maryland USA; 4 School of Life Sciences, École Polytechnique Fédérale de Lausanne (EPFL), CH-1015 Lausanne, Switzerland; 5 Department of Neurosciences, School of Medicine, University of California at San Diego, San Diego, California USA; 6 Neuromuscular Division, Johns Hopkins University School of Medicine, Baltimore, Maryland USA; 7 Department of Biochemistry and Molecular Biology II, Faculty of Pharmacy, University of Granada, Granada, Spain; 8 Department of Human Genetics, Medical Faculty, University of Saarland, Homburg/Saar, Germany; 9 MRC Human Genetics Unit, Institute of Genetics and Molecular Medicine (IGMM), University of Edinburgh, Western General Hospital, Edinburgh, UK

## Abstract

**Background:**

Amyotrophic lateral sclerosis (ALS) is a fatal neurodegenerative disease involving loss of motor neurons and having no known cure and uncertain etiology. Several studies have drawn connections between altered retrotransposon expression and ALS. Certain features of the LINE-1 (L1) retrotransposon-encoded ORF1 protein (ORF1p) are analogous to those of neurodegeneration-associated RNA-binding proteins, including formation of cytoplasmic aggregates. In this study we explore these features and consider possible links between L1 expression and ALS.

**Results:**

We first considered factors that modulate aggregation and subcellular distribution of LINE-1 ORF1p, including nuclear localization. Changes to some ORF1p amino acid residues alter both retrotransposition efficiency and protein aggregation dynamics, and we found that one such polymorphism is present in endogenous L1s abundant in the human genome. We failed, however, to identify CRM1-mediated nuclear export signals in ORF1p nor strict involvement of cell cycle in endogenous ORF1p nuclear localization in human 2102Ep germline teratocarcinoma cells. Some proteins linked with ALS bind and colocalize with L1 ORF1p ribonucleoprotein particles in cytoplasmic RNA granules. Increased expression of several ALS-associated proteins, including TAR DNA Binding Protein (TDP-43), strongly limits cell culture retrotransposition, while some disease-related mutations modify these effects. Using quantitative reverse transcription PCR (RT-qPCR) of ALS tissues and reanalysis of publicly available RNA-Seq datasets, we asked if changes in expression of retrotransposons are associated with ALS. We found minimal altered expression in sporadic ALS tissues but confirmed a previous report of differential expression of many repeat subfamilies in *C9orf72* gene-mutated ALS patients.

**Conclusions:**

Here we extended understanding of the subcellular localization dynamics of the aggregation-prone LINE-1 ORF1p RNA-binding protein. However, we failed to find compelling evidence for misregulation of LINE-1 retrotransposons in sporadic ALS nor a clear effect of ALS-associated TDP-43 protein on L1 expression. In sum, our study reveals that the interplay of active retrotransposons and the molecular features of ALS are more complex than anticipated. Thus, the potential consequences of altered retrotransposon activity for ALS and other neurodegenerative disorders are worthy of continued investigation.

**Electronic supplementary material:**

The online version of this article (10.1186/s13100-018-0138-z) contains supplementary material, which is available to authorized users.

## Background

With the discovery in 1950 of transposable elements (TEs) genomes began to seem far more dynamic than hitherto conceived [1]. It is now clear that TEs have been important long-term drivers of genome evolution. Year by year, more and more ways in which mobile DNA impacts gene expression and integrity, cell variability and viability, and ultimately human health are revealed. With recent discoveries that TEs are active not only in the germline but also in somatic cells, it is evident that each of us is a mosaic of different genomes that now seem dynamic indeed (reviewed by [2] and many others).

Retrotransposon TEs include long terminal repeat (LTR) and non-LTR class elements. Both retrotranspose by a “copy and paste” mechanism involving reverse transcription of an RNA intermediate and insertion of its cDNA copy at a new site in the genome. LTR-retrotransposons, including human endogenous retroviruses (HERVs), are remnants of past germ line infections by retroviruses that subsequently lost their ability to reinfect cells. While the HERV-K(HML-2) group includes some polymorphic proviral loci [3, 4], human LTR retrotransposons generally are insertionally inactive, although many remain capable of transcription. Long Interspersed Element-1 (LINE-1, L1) retrotransposons are the only active autonomous mobile DNA in humans. Alone they occupy at least 17% of our genome and have also been responsible for the insertion *in trans* of thousands of processed pseudogenes and a million non-autonomous Short Interspersed Elements (SINEs), including Alu and SVA (composite SINE/VNTR/Alu) elements [5]. The 6.0 kilobase (kb) bicistronic human L1 has a 5' untranslated region (UTR) that functions as an internal promoter, two open reading frames (ORF1 and ORF2), and a 3' UTR. A weak promoter also exists on the antisense strand of the human L1 5' UTR [6]. ORF2 encodes a 150-kilodalton (kD) protein with DNA endonuclease and reverse transcriptase (RT) activities. While the 40 kD ORF1p RNA-binding protein is essential for retrotransposition, its exact role in retrotransposition is unclear, although it possesses RNA chaperone and packaging properties [7–9]. The great majority of L1s in the genome are 5' truncated and otherwise rearranged or mutated and so incapable of autonomous transcription.

There are 145 fully intact L1s in the human genome of which cell culture retrotransposition assays suggest about 100 remain potentially mobile in any individual diploid genome [10–12]. There are also hundreds of full length L1s lacking intact ORFs but possibly capable of generating protein [13, 14]. While L1 expression is normally suppressed by a host of cellular factors, the suppression is relaxed in embryonic stem cells, the early embryo, and some cancers (reviewed in [15, 16]).

Notably, retrotransposons are also active in some brain cells [17]. Loss of piRNA pathway proteins correlates with elevated retrotransposon expression in Drosophila brain [18], although in mammals this pathway seems to act primarily in the germ line to control retrotransposon activity (see [19] for review). Early studies showed L1 retrotransposition in dividing neuronal progenitor cells (NPCs), especially those of the hippocampus [20, 21], and subsequently in non-dividing neurons [22, 23]. High-throughput sequencing of single neurons confirmed endogenous L1 retrotransposition in the human brain, although frequency estimates differed significantly (reviewed in [24–27]). Thus, it has been proposed that L1 activity contributes to neuronal plasticity [28]. Elevated L1 retrotransposition has also been reported for several human neurological conditions, including ataxia telangiectasia [29], Rett syndrome [30], autism [31], schizophrenia [32, 33], and major depressive disorder [34], as well as in neuronal cell lines or brains of patients exposed to opioids [35–37], and in brains of a mouse model of Huntington's disease [38] and hippocampi of mice following novel exploration [39] or diminished maternal care [40]. However, some of these studies relied solely on DNA amplification by quantitative (q)PCR or digital droplet PCR to compare L1 insertion copy differences between test and normal states, strategies that may fail to distinguish between *bona fide* genomic L1 insertions and contaminating extrachromosomal L1-derived nucleic acids. Some studies may therefore warrant additional verification (see also [41, 42] and discussion).

Several studies have also drawn connections between altered expression of LTR retrotransposons and amyotrophic lateral sclerosis (ALS). ALS is a fatal neurodegenerative disease involving loss of upper and lower motor neurons and afflicts 2 in 100,000 people each year. Death typically follows 2 to 3 years after onset and, while about 90% of cases are sporadic, the rest have a family history of the disease. There are no current means to reverse the course of ALS, and treatment involves efforts to slow progression of symptoms [43]. ALS has overlapping clinical presentations with frontotemporal lobar degeneration (FTLD) and its most common subtype frontotemporal dementia (FTD), a neurolgical condition affecting the frontal and temporal lobes and marked by cognitive and behavioral impairment. About 20% of ALS patients also exhibit FTLD, and ALS and FTLD have been seen as part of a continuous disease spectrum [44].

Increased reverse transcriptase activity from an unknown source is detectable in sera and cerebrospinal fluids of non-HIV-infected ALS patients [45–48]. Douville et al. [49] correlated this RT activity with elevated expression of a few HERV-K(HML-2) loci and increased amounts of *pol* gene transcripts and RT protein in cortical neurons of some ALS patients. Hadlock et al. [50] noted elevated immune response to HERV-K(HML-2) Gag protein in serum samples from ALS patients, and recently it was shown that overexpression of the HERV-K(HML-2) envelope protein causes motor neuron toxicity and motor dysfunction in transgenic mice [51]. However, it cannot be excluded that some of the elevated RT activity observed is also due to increased expression of LINE-1 retrotransposons. It is reasonable to presume that cellular changes that increase HERV-K(HML-2) expression in ALS patients may similarly activate other retrotransposons. Indeed, a recent study reported global increases in expression of selected families of both LTR and non-LTR retrotransposons in ALS and FTLD patients with a hexanucleotide expansion in the Chromosome 9 Open Reading Frame 72 (*C9orf72*) ALS gene but not in sporadic ALS cases or controls [52].

The accumulation of neuronal RNA and protein aggregates, including cytoplasmic stress granules (SGs), is a pathogenic hallmark of a number of neurodegenerative diseases. Pathological aggregation of RNA-binding proteins has been implicated in ALS, FTLD, Alzheimer's disease, spinocerebellar ataxia, Huntington’s disease, and inclusion body myositis. In the case of ALS, there is increasing evidence that abnormal RNA processing and abnormal self-aggregation of proteins, leading to altered RNA granule formation and malfunction of protein pathways, contribute to motor neuron death [53]. A key pathological feature of ALS is the presence of cytoplasmic inclusions in degenerating motor neurons and oligodendrocytes. Inclusions are not restricted to the spinal cord and motor cortex but are present in other brain regions, such as the frontal and temporal cortices, hippocampus, and cerebellum, and are especially evident in patients with accompanying FTD [54]. What triggers protein aggregation and what it means for cell pathology and progression of the disease remain unclear.

Aggregation of TAR DNA binding protein 43 (TDP-43, product of the *TARDBP* gene) is especially interesting as a unifying pathological marker of both FTLD and ALS. Mutations in *TARDBP* are involved in about 4% of familial (fALS) and 1% of sporadic ALS (sALS) cases. However, even lacking a mutation, TDP-43 protein, while typically nuclear in healthy cells, is cleaved and hyperphosphorylated and accumulates in ubiquitinated cytoplasmic inclusions in almost all ALS and almost half of FTLD patients (reviewed in [55]). TDP-43 protein aggregation pathology also characterizes other neurodegenerative disorders, including Parkinson's [56], Alzheimer's [57] and Huntington's [58] diseases, and inclusion body myopathies [59].

Several features of LINE-1-encoded ORF1p are reminiscent of neurodegeneration-associated proteins. ORF1p is an ubiquitinated and phosphorylated RNA-binding protein prone to forming cytoplasmic aggregates, including SGs [60–63]. Therefore, it is conceivable therefore that abnormal expression of ORF1p in neuronal cells might aggravate formation of cytoplasmic aggregates and contribute to disease pathology. Here we analyzed subcellular localization and aggregation features of LINE-1 ORF1p and ways they may be analogous to or differ from those of neurodegeneration-associated RNA-binding proteins. We show that some ALS-associated RNA-binding protein mutants closely associate with ORF1p in cytoplasmic RNA granules of tumor cell lines, and that increasing the expression of some ALS proteins, including TDP-43, inhibits L1 retrotransposition in a cell culture reporter assay. We also considered the possibility that LINE-1 retrotransposon activity may be associated with ALS disease. Reverse transcription (RT)-qPCR) analyses failed to detect significantly altered expression of non-LTR Alu or L1 elements in sALS tissues. However, by reanalyzing publicly available RNA-Seq datasets, one previously examined for TE levels [52] and one hitherto untested, we confirmed misregulation of selected TE subfamilies in *C9orf72* gene-related ALS samples. While so far the evidence is not compelling for ALS, we believe the potential for altered non-LTR retrotransposon expression playing a role in neurodegenerative disorders is worthy of continued investigation.

## Results

### A common LINE-1 polymorphism alters formation of ORF1p cytoplasmic RNA granules

**Fig. 1:**
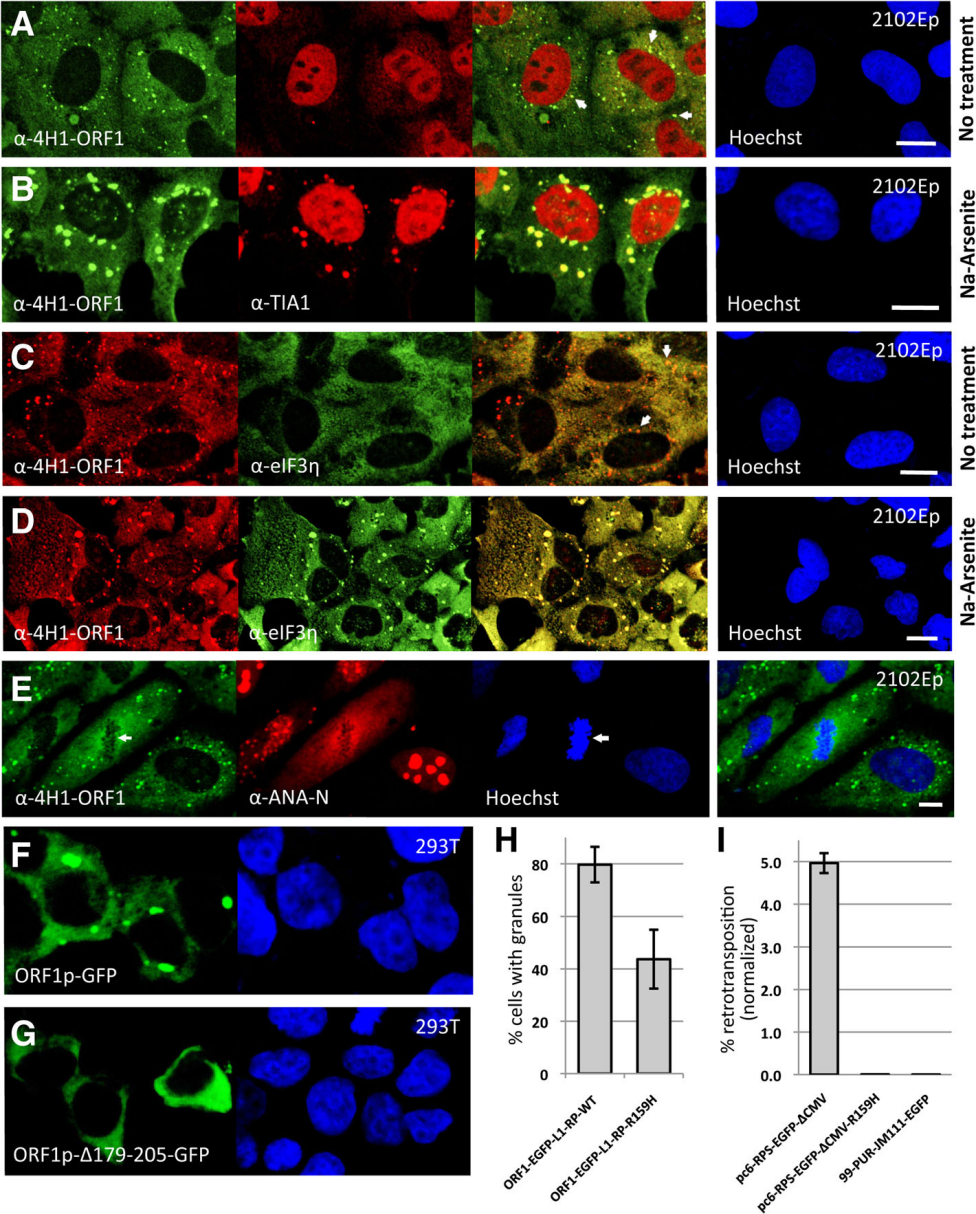
Cytoplasmic localization of L1 ORF1 protein. Stress granule marker proteins TIA1 (**a**, **b**) and eIF3η (**c**, **d**) show minimal colocalization with endogenous ORF1p in cytoplasmic granules (shown by arrows) of unstressed human embryonal carcinoma 2102Ep cells (**a**, **c**) but colocalize in large stress granules of cells treated with Na-arsenite (0.5 mM for 1 hour) (**b**, **d**). Cell nuclei are stained with Hoechst. Size bars are 10 μm. **e** ORF1p cytoplasmic granules retain integrity during cellular mitosis. Patient sera-derived α-ANA-N marks nucleoli [60]. Endogenous ORF1p is mostly excluded from metaphase chromatin plates (arrow), as shown by Hoechst staining (see arrow). **f** Ectopically expressed EGFP-tagged human ORF1p induces prominent cytoplasmic granules, but (**g**) deletion of its Q-N-rich region abolishes granule formation. **h** The ORF1p R159H point mutation reduces cytoplasmic granule formation by 50%. Approximately 500 cells were examined. **i** The R159H mutation abolishes cell culture LINE-1 retrotansposition. pc6-RPS-EGFP-ΔCMV wild-type or R159H mutant retrotransposition reporter constructs were transfected in HEK 293T cells and 5 days later the percentages of EGFP-positive cells were determined by flow cytometry. The construct 99-PUR-JM111-EGFP served as a negative control for retrotransposition [84]. Each construct was tested in quadruplicate wells with results for one biological replicate shown

Early studies showed that endogenous ORF1p concentrates in cytoplasmic granules in cultured cells or fixed tissues [60, 64]. Overexpression or cell stress causes ORF1p to enter stress granules (Fig. 1F) [60, 61, 65, 66]. Here we used the monoclonal α-human α-4H1 ORF1 antibody (Millipore Sigma; [67]), which targets ORF1p N-terminal amino acids (aa) 35 to 44, to examine expression of endogenous ORF1p in 2102Ep cells, an embryonal germ cell teratocarcinoma line that has unchanging human embryonal stem cell (hESC) characteristics [68, 69]. By Western blotting, α-4H1-ORF1 detected a robust approximately 42 kD band of size consistent with the ORF1p monomer, a much weaker 85 kD band that likely marks ORF1p dimers, and sporadically a band of 65 kD of unknown identity (Additional file 1: Figure S1A). Single, less intense 42 kD bands were seen in human embryonic kidney HEK 293T and neuroblastoma SH-SY5Y cells. SH-SY5Y is a thrice-cloned sub-line of the bone marrow-derived neural line SK-N-SH, which shows significantly less ORF1p signal. No endogenous ORF1p was detected in human cervical adenocarcinoma HeLa cells (Additional file 1: Figure S1A).

Confirming previous results [60], constitutive aggregation of ORF1p in the cytoplasm was detected in multiple cell lines by multiple α-ORF1p antibodies (Additional file 1: Figure S1B-E). However, the pattern and degree of granule formation by ORF1p can vary significantly with cell type. In human neuroblastoma SH-SY5Y cells, for example, ORF1p granules are rare in the main cytoplasm but evident in neurite outgrowths (Additional file 1: Figure S1C). Notably, endogenous ORF1p cytoplasmic aggregates differ from SGs in certain ways. In the absence of external stress, in 2102Ep cells small ORF1p aggregates are numerous but only faintly and rarely marked by SG proteins such as cytotoxic granule associated RNA binding protein (TIA1) and elongation initiation factor 3 (eIF3η) (Fig. 1A, C). Furthermore, unlike SGs [70], endogenous ORF1p granules do not obviously dissemble during cell mitosis (Fig. 1E). As previously reported, when exposed to sodium arsenite, an inducer of oxidative stress, ORF1p redistributes to larger-sized aggregates that now mostly colocalize with SG proteins (Fig. 1B, D). Thus ORF1p aggregates form constitutively but do not chronically induce a cellular stress state that is marked by redistribution of SG proteins.

Previously, it was shown that overexpressed ORF2p and L1 RNA also colocalize with ORF1p in cytoplasmic aggregates [60, 61, 63, 71]. Interestingly, we noted [72], and others confirmed [73], that ORF2p is visible in only a minor percentage of ORF1p-positive cells when the two proteins are coexpressed from an L1 construct. The reason for this is unknown but may relate to an unconventional translation mechanism of LINE-1 ORF2 [74]. Unfortunately, although α-ORF2p antibodies exist [75–79], they are not widely available or are ineffective in detecting endogenous ORF2p, and so we did not examine ORF2p localization in this study.

Many RNA-binding proteins that form SGs have intrinsically disordered prion-like domains rich in glutamine and asparagine (Q-N) residues. Aggregation of prion-like domain proteins is characteristic of various neurodegenerative disorders including ALS (reviewed in [80]). No prion-like domain is predicted in ORF1p using the PrionW [81] or PLAAC [82, 83] algorithms. However, human (but not mouse) L1 ORF1p contains a Q-N-rich internal region (36% Q or N between residues 179 and 205, numbering according to accession number AF148856.1). Deletion of this region abolishes granule formation (Fig. 1F, G), indicating it is critical for human L1 ORF1p aggregation properties.

Previously, we and others [60, 61] showed that mutations in the N-terminus leucine zipper domain or the C-terminal domain double-point mutation R261/262A (the so-called JM111 mutation that abrogates cell culture retrotransposition; [84]) also alter ORF1p cytoplasmic aggregation. We also reported that a non-conservative mutation, R159G, inhibits ORF1p granule formation. This residue was subsequently shown to be important for RNA-binding and is within the RNP2 sequence of the ORF1p RRM (RNA recognition motif) [85]. In the present study, to ascertain the prevalence of L1s in the human genome with R159 polymorphisms, we queried the L1Base2 database [13]. L1Base2 is subdivided into 3 categories: L1s with intact ORF1 and ORF2 (FLI-L1s), L1s with intact ORF2 but disrupted ORF1 (ORF2-L1s), and non-intact L1s >4500 nucleotides in length (FLnI-L1s). Although the R159G variant was detected at only very low frequency (0.47% of 6346 alignable FLnI-L1 sequences), many other R159 polymorphisms were found, with R159H being most common. In all, we identified R159 changes to histidine, cysteine or proline residues in 4.8% of FLI-L1s, 11.5% of ORF2-L1s, and 40.3% of FLnI-L1s (Additional file 1: Figure S2A). Thus, sequence variation in the aggregation-control R159 codon of human L1 ORF1p is common in endogenous L1s.

We introduced the R159H change into ORF1-EGFP-L1-RP, a construct with CMV promoter and ORF1 C-terminally tagged with EGFP followed by intact downstream L1 sequence, and as expected observed a 30% decline in the number of HEK 293T cells with ORF1p cytoplasmic granules (Fig. 1H). We next tested the effect of the R159H polymorphism in a cell culture retrotransposition assay. In this assay, an enhanced green fluorescent protein (EGFP) reporter gene reporter cassette, interrupted by a backwards γ-globin intron, is inserted in opposite transcriptional orientation into the 3' UTR of L1-RP (a highly active human L1 [86]). The EGFP reporter gene can be expressed from its own promoter only after the L1 is transcribed, the γ-globin intron is removed by splicing, the L1-reporter cassette hybrid transcript is reverse-transcribed, and its cDNA inserted in the genome [84, 87]. The R159H mutation abolished cell culture retrotransposition to levels similar to that observed for an L1 containing the ORF1p JM111 mutation that cannot form a functional L1 ribonucleoprotein (RNP) complex [88] (Fig. 1I).

Finally, we considered the possibility that the abundance of R159 polymorphisms might be due to a CpG dinucleotide methylation hotspot. Following genome bisulfite conversion, PCR amplification, cloning of the amplicons and Sanger sequencing, we queried the methylation status of nine CpGs within a 436-nt stretch (1169-1604) of ORF1 surrounding the R159 codon. Although CpGs were methylated (16 to 64%), we observed no preference for methylation at the ORF1 R159 codon (Additional file 1: Figure S2B).

Thus, L1 ORF1 polymorphisms can alter not only retrotransposition efficiency but also ORF1p aggregation dynamics for a subset of L1s abundant in the human genome.

### LINE-1 ORF1 protein concentrates in nuclear aggregates

**Fig. 2:**
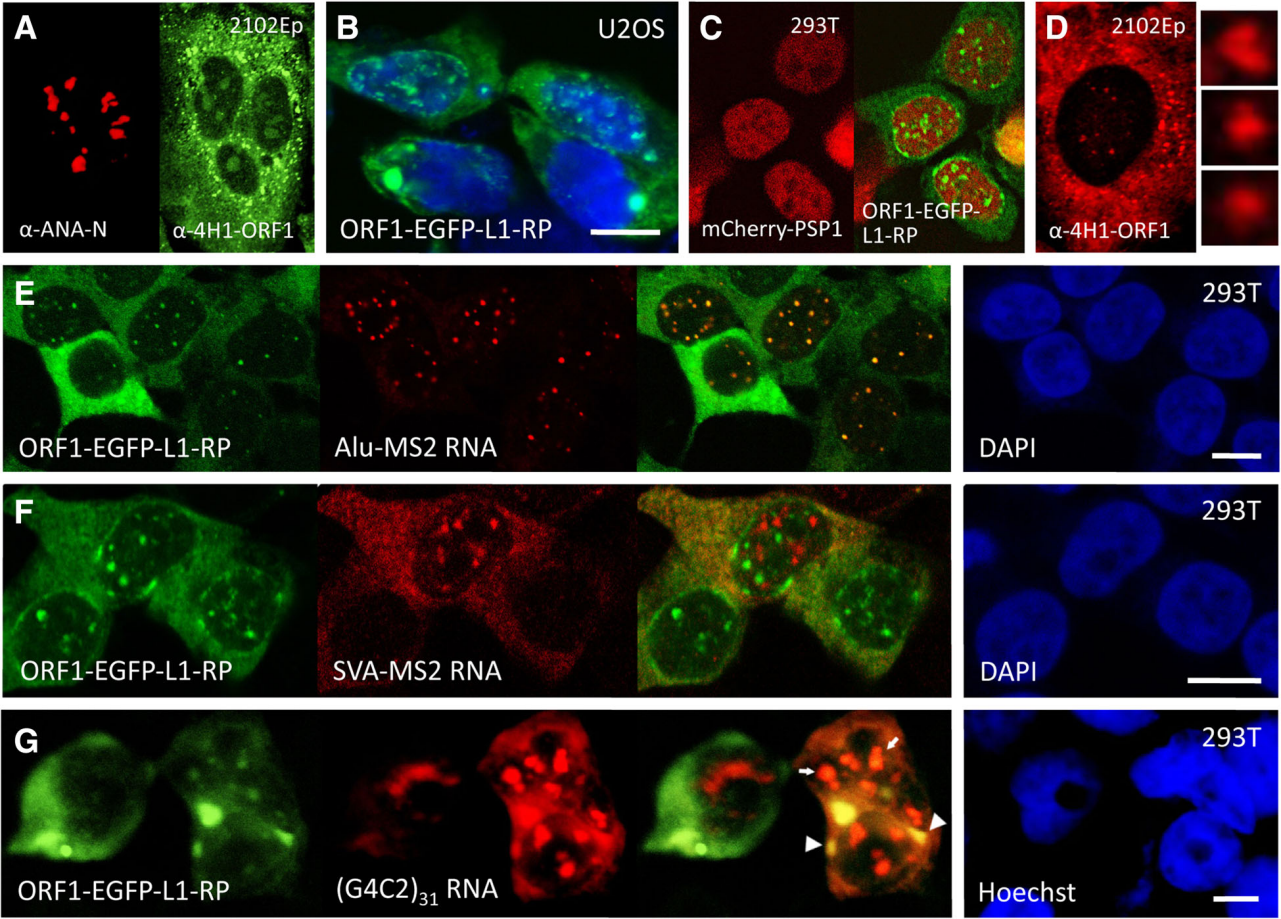
Nuclear localization of L1 ORF1 protein. **a** Endogenous ORF1p detected in 2102Ep cells by the α-4H1-ORF1 antibody. ORF1p is mostly cytoplasmic where it concentrates in granules and occasionally at the nuclear membrane. It is faintly seen in the nucleoplasm and concentrates in nucleoli of a subset of cells. **b**, **c** Exogenously expressed EGFP-tagged ORF1p strongly concentrates at the nuclear membrane and in perinucleolar foci of 5% or fewer human (**b**) U2OS or (**c**) HEK 293T cells with attendant reduction in size and number of cytoplasmic granules. Cotransfected mCherry-PSP1 marks nuclei and is excluded from nucleoli. **d** Endogenous ORF1p detected by α-4H1-ORF1 also forms discrete nuclear foci in a minor percentage of 2102Ep cells. Selected foci are enlarged in panels to the right. **e** Alu RNA, tagged with six MS2 coat protein recognition stem loops and expressed from construct pBS 7SL Alu-MS2 (Ya5), was detected by FISH using a Cy3-tagged DNA probe to the MS2 stem loops. Alu RNA colocalizes with nuclear foci marked by EGFP-tagged ORF1p in HEK 293T cells. **f** Nuclear foci of MS2 stem loop-tagged full-length SVA RNA detected by the Cy3-MS2 DNA probe do not colocalize with foci marked by ORF1p-EGFP. **g** RNA having 31 tandem G4C2 repeats detected by FISH using a Cy3-conjugated (C4G2)_4_ DNA probe induces intense intranuclear or cytoplasmic RNA aggregates that colocalize with ORF1p-EGFP in a minor percentage of HEK 293T cells (nuclear granules are marked by small arrows and cytoplasmic granules by large arrows). Size bars are 10 μm

Previously we showed that both overexpressed and endogenous L1 ORF1p are not only cytoplasmic but colocalize with nucleoli of a subset of cells (Fig. 2A). Overexpressed ORF2p also enters nucleoli [76]. Here we report that exogenously expressed GFP-tagged human ORF1p also strongly concentrates at the nuclear membrane and forms small discrete perinucleolar foci in 5% or fewer of human osteosarcoma U2OS or HEK 293T cells. These cells show an attendant reduction in the size and number of cytoplasmic granules (Fig. 2B,C). Consistently, endogenous ORF1p nuclear foci are also seen in a small fraction of 2102Ep cells (Fig. 2D); presence of the foci in nuclei was confirmed by z-series confocal imaging. Recently, De Luca et al. [79] also showed in human melanoma cells both endogenous ORF1p and ORF2p in nuclear puncta that partially colocalized.

Using the MS2-NLS-GFP detection protocol [89], we previously reported that overexpressed Alu SINE RNA forms small distinct nuclear foci that partially associate with coiled (Cajal) body marker proteins [72]. Coiled bodies are nuclear non-membrane RNP suborganelles involved in the processing of non-coding RNAs and have been linked with the rare motor neuron disease spinal muscular atrophy (SMA) [90]. In our present experiments, we show that in the minor percentage of HEK 293T cells that form ORF1p nuclear foci, these foci closely colocalize with coexpressed MS2-tagged Alu RNA (Fig. 2E, detected here by fluorescent in situ hybridization (FISH)). Thus, ORF1 protein and Alu RNA may directly interact in the nucleus. SVA SINE RNA expressed from plasmid pcDNA SVA_SPTA1_-MS2 is mainly cytoplasmic but also forms nuclear foci [72]. Interestingly, these foci do not colocalize with ORF1-EGFP foci, despite the fact that both Alu and SVA RNAs depend upon L1 for their retrotransposition and insert in the genome by a common mechanism (Fig. 2F). As previously reported, L1 RNA failed to form nuclear foci in our experiments [61, 72].

Certain neurodegenerative conditions, including myotonic dystrophy, fragile X-associated tremor ataxia syndrome and spinocerebellar ataxias, are associated with genes that undergo long simple repeat expansion mutations. RNAs transcribed from these mutant genes accumulate in nuclear foci [91]. A pathogenic GGGCC (G4C2) hexanucleotide expansion in intron 1 of the *C9orf72* gene is the most common mutation associated with both ALS and FTD [92, 93] and is implicated in Huntington's disease [94]. Mutant *C9orf72* gene transcripts form toxic RNA foci in affected neuronal cells and are associated with the disease pathology [92, 95]. We transiently coexpressed in HEK 293T cells ORF1p-EGFP together with *C9orf72* RNA having 31 tandem G4C2 repeats, the latter detected by RNA FISH using a Cy3-conjugated (C4G2)_4_ probe [96]. As with Alu SINE RNAs, ORF1p-EGFP granules directly overlapped or juxtaposed with G4C2_31_ RNA granules in nuclei and cytoplasm of some cells (Fig. 2G).

Thus, being a promiscuous RNA-binding protein, it is possible that L1 ORF1p is able to bind and sequester many cellular RNAs in granules present in both the cytoplasm and nucleus.

### The control of LINE-1 ORF1p nuclear localization in 2102Ep cells

Several studies have reported that cell division facilitates efficient retrotransposition, citing a failure of L1s to retrotranspose in cultured primary and tumor cells blocked at G_0_ phase but disagreeing on the extent of retrotransposition loss in G_1_/S-arrested cells (reporting a 3-fold to 10-fold decline; [97–99]). Mita et al. [100] recently reported that cell culture retrotransposition occurs preferentially in S-phase. On the other hand, we previously showed significant retrotransposition in non-dividing neuronal cells differentiated from hESCs [23], and similar data was earlier observed in transformed cell lines [97]. Since ORF1 protein is essential for active retrotransposition [84], we chose to examine two mechanisms postulated to control ORF1p subcellular localization, cell cycle and active nuclear export.

2102Ep cells are nullipotent, manifesting a stable phenotype; indeed, they are used as a reference to characterize newly derived hESC lines [69]. However, we noticed considerable variation in the percentage of 2102Ep cells showing obvious nucleolar localization of endogenous ORF1p when examining clusters of cells across a single slide using immunofluorescence (IF) and the α-4H1-ORF1 antibody (between 0.6% and 36% of total cells randomly examined in three separate experiments). Less densely clustered cells more frequently showed ORF1p nucleolar concentration. Germline tumor cells, including 2012Ep cells, are altered for cell cycling by cell-to-cell contact inhibition or serum depletion [101], and we therefore wondered if concentration of ORF1p in the nucleus might relate to cell cycle status.

**Fig. 3:**
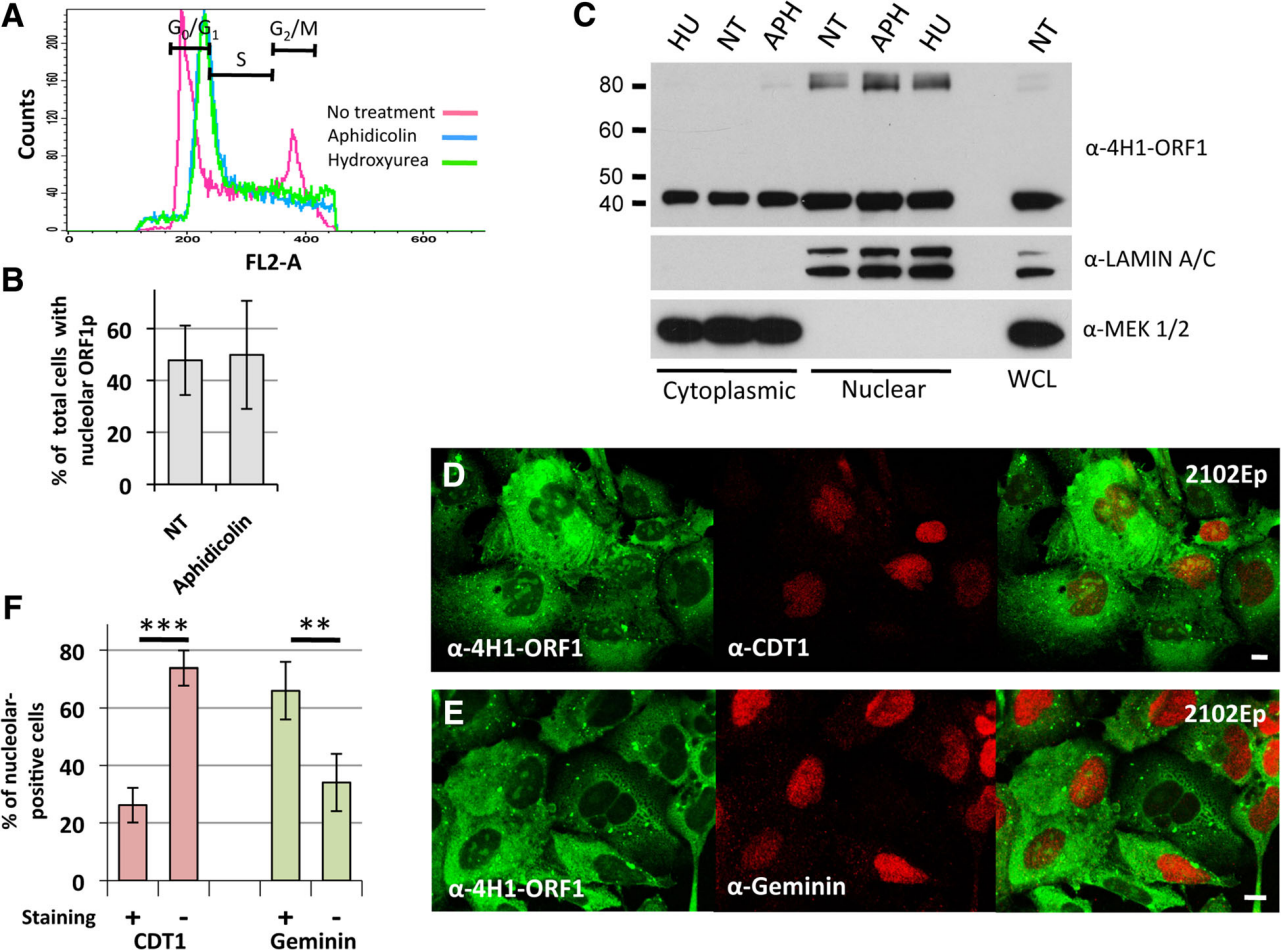
ORF1p nuclear localization in 2102Ep cells is not strictly influenced by cell cycle status. **a** Cell cycle arrest was induced for 22 hours with 10 μg/ml aphidicolin or 3 mM hydroxyurea and confirmed by propidium iodide staining and flow cytometry. The percentages of cells in G_0_/G_1_, S or G_2_/M phases were determined using BD CELLQuest software (BD Biosciences). FL2-Area is plotted against cell counts. **b** Percentage of cells having visible nucleolar localization when not treated (NT) or treated with aphidicolin to induce cell cycle arrest at G_0_/G_1_ phase. **c** Endogenous ORF1p was detected by Western blotting in both nuclear and cytoplasmic cell fractions left untreated (NT) or treated with aphidicolin (APH) or hydroyxurea (HU). Purities of nuclear and cytoplasmic fractions are shown using α-Lamin A/C and α-MEK1/2 antibodies, respectively. WCL: whole cell lysate. **d**,**e**) Immunofluorescence of 2102Ep cells showing that cells both with or without nucleolar ORF1p localization can express CDT1 or Geminin. **f** The percentages of untreated 2102Ep cells having visible ORF1p nucleolar localization that are marked (+) or unmarked (-) by α-CDT1 (red bars) or Geminin (green bars) staining. The data summarizes three replicate experiments with at least 400 cells scored for each experiment. Statistical significance was calculated by Student’s t-test (** p<0.01; *** p<0.001)

Accordingly, we seeded 2102Ep cells at low densities and blocked G_1_/S phase transition using aphidicolin, an inhibitor of DNA polymerase α, or hydroxyurea, which inhibits ribonucleotide reductase causing a loss of deoxyribonucleotides [102, 103], and examined effects on endogenous ORF1p nucleolar localization. Cell cycle blockage was confirmed by propidium iodide staining followed by flow cytometry (Fig. 3A). Blocking the cell cycle at G_1_/S had no significant effect on the average percentage of cells with endogenous ORF1p nucleolar localization (Fig. 3B), or on nuclear-cytoplasmic levels following cell fractionation and Western blotting (Fig. 3C). For both treated and non-treated 2102Ep cells, Western blotting showed a major amount of endogenous ORF1p in the nuclear fraction, in agreement with Sokolowski et al. [104] who reported nuclear fraction concentration of plasmid-expressed ORF1p in human HeLa and mouse NIH3T3 cells. However, it is likely that some of the ORF1p we detect in the nuclear fraction is due to copurification of insoluble ORF1p cytoplasmic aggregates [105]. Significantly, however, the amounts of ORF1p detected by Western blotting in both cytoplasmic and nuclear fractions remained unaltered by cell cycle blockage.

We next stained untreated 2102Ep cells with antibodies to chromatin licensing and DNA replication factor 1 (CDT1), a G_1_ phase nuclear protein lost after initiation of S phase [106], or Geminin (GMNN), a protein expressed only in S/G2/M phases [107], and then examined cells for L1 ORF1p nucleolar concentration. Immunocytochemistry showed that both CDT1 and Geminin marked 2102Ep cells with or without endogenous ORF1p visible in nucleoli (Fig. 3D, E). However, a majority of cells showing nucleolar ORF1p failed to stain with CDT1, while the opposite was true for Geminin (Fig. 3F). This suggests partial nucleolar exclusion of ORF1p during G1 phase but without stringent cell cycle control. Our results in part contradict a recently published observation [100] that overexpressed LINE-1 ORF1p is nuclear in HeLa cells expressing CDT1 (G1 phase) and almost completely cytoplasmic in cells expressing Geminin (see Discussion).

Next, we considered if LINE-1 ORF1p shuttles between the nucleus and cytoplasm, as is the case with TDP-43 and some other prion-domain RNA-binding proteins associated with neurodegenerative diseases [108]. Shuttling proteins often contain nuclear export signals (NESs), consisting of a short stretch of hydrophobic leucine-rich residues [109]. We previously reported that subcellular localization of overexpressed GFP-tagged ORF1p in HEK 293T cells was unaltered by leptomycin B (LMB), a chemical inhibitor of the chromosomal region maintenance 1 (XPO1/CRM1) nuclear export pathway [76]. We now observed that treatment of 2102Ep cells with 55 nM LMB for 18 hours also had no obvious effect on endogenous ORF1p localization (Additional file 1: Figure S3A). On the other hand, controls revealed that LMB efficiently inhibited cytoplasmic export of endogenous cyclin B1, which contains an NES responsive to CRM1 (Additional file 1: Figure S3B) [110], as well as a GFP-tagged phosphorylation mimetic mutant of MAPKAP kinase 2 (MK2-mut T205/317E) that remains in the cytoplasm once exported from the nucleus (Additional file 1: Figure S3C) [111].

Previously, we fused a suspected ORF1p leucine-rich NES (aa 87-93, LKELMEL) and linker to the C-terminus of EGFP. While a functional NES should cause EGFP, which is normally both cytoplasmic and nuclear, to become more cytoplasmic [112], we failed to observe increased concentration of EGFP-LKELMEL in the cytoplasm [76]. For the present study, we used the NetNES 1.1 Server [113] to predict a second NES site at the C-terminus of ORF1p (ORF1 aa 313-321, LKELLKEAL). Fusing this sequence to the N-terminus of EGFP also failed to alter distribution of EGFP (Additional file 1: Figure S3D). Moreover, altering the sequence to encode LKEAAAAAL in construct ORF1-EGFP-L1-RP failed to visibly affect its ORF1p localization (Additional file 1: Figure S3E).

In contrast to our results, Mita et al. [100] reported a 20 to 35% increase in nuclear retention of exogenous ORF1p overexpressed in HeLa cells treated with LMB. While we failed to detect NES sequences in ORF1p or obvious sensitivity to the CRM1 export pathway, we cannot exclude the possibility that LMB causes nuclear retention of minor amounts of endogenous ORF1p not visibly obvious in our system. Also, despite previous reports of attenuated cell culture retrotransposition following G_1_/S phase arrest, our results suggest this is not due to failure of ORF1p RNPs to enter nuclei, at least in 2102Ep cells, which are known to accommodate cell culture retrotransposition [114]. Moreover, despite the previous suggestion that nuclear membrane breakdown is required for nuclear entry of L1 ORF1p [100] this does not appear to be the case for this cell line.

### ALS-related protein mutants colocalize with ORF1p in cytoplasmic granules

To date at least 25 genes have been linked to ALS [43, 53]. The first ALS gene discovered, superoxide dismutase (*SOD1*) [115], is mutated in about 20% of familial cases. *C9orf72* is by far the most frequent gene accounting for about 35% of fALS, 25% of fFTD, and 6% of sALS cases [92, 93, 116]. RNA-binding protein FUS (*FUS*) and *TARDBP* mutations each account for about 4% of fALS cases. Other ALS-associated genes, including alsin (*ALS2*), angiogenin (*ANG*), heterogeneous nuclear ribonucleoprotein A1 (*HNRNPA1*), optineurin (*OPTN*), sequestosome-1 (*SQSTM1*/*P62*), ubiquilin 2 (*UBQLN2*), TANK binding kinase 1 (*TBK1*), valosin-containing protein (*VCP*) and VAMP-associated protein B and C (*VAPB*) among others, account for only a small percentage of cases so confounding treatment strategies. ALS animal models of neurodegeneration have mostly examined the toxic effects of overexpressing disease-related aggregation-prone proteins. Mutants of several ALS-associated RNA-binding proteins are known to shift localization from the nucleus to the cytoplasm and form RNA foci in the disease state [117, 118].

**Fig. 4:**
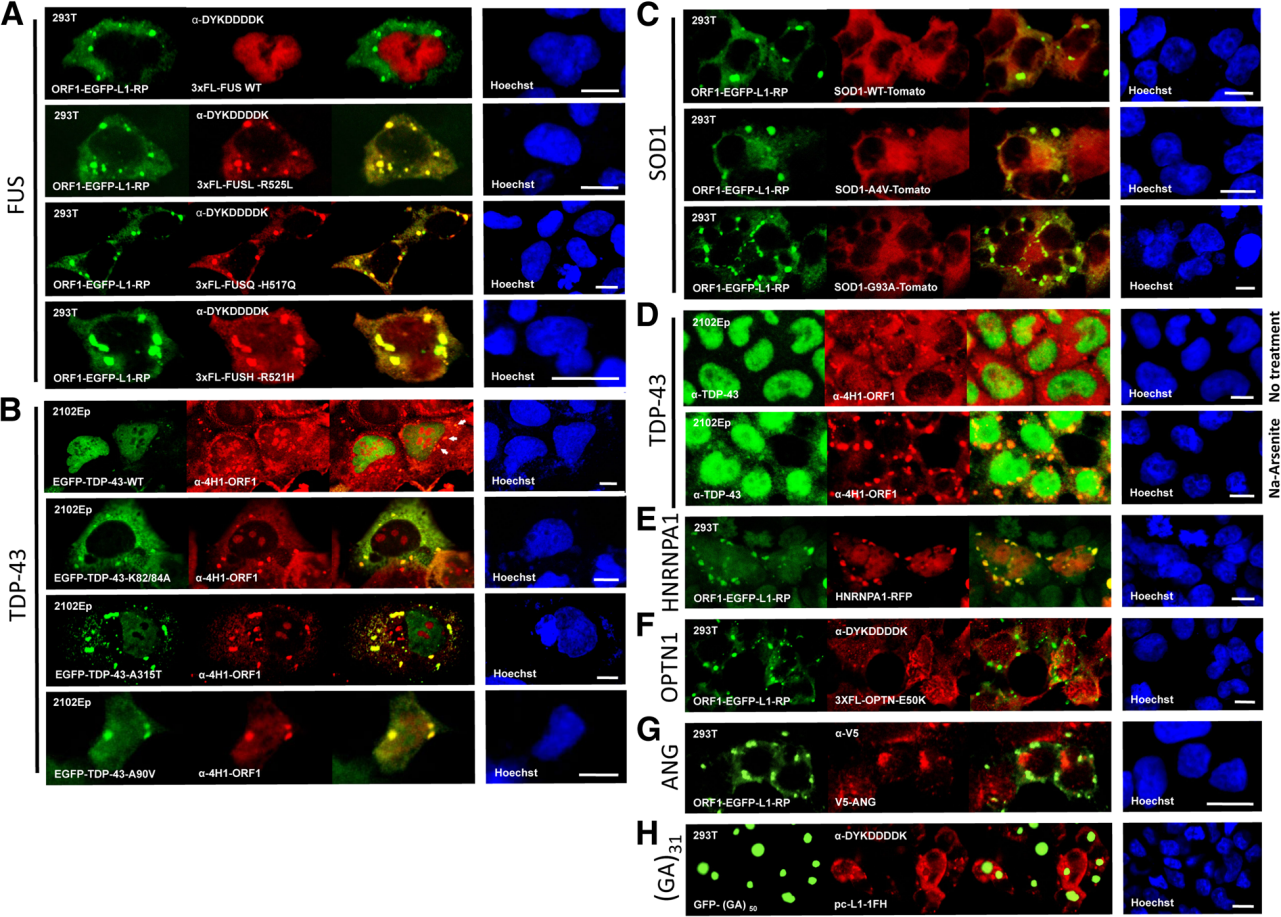
ALS-associated protein mutants colocalize with ORF1p in cytoplasmic aggregates of HEK 293T and 2102Ep cells. **a** Exogenously expressed wild-type 3XFLAG-tagged FUS protein is nuclear and only rarely colocalizes with ORF1-EGFP in the cytoplasm, but some mutants strongly overlap in cytoplasmic granules of unstressed HEK 293T cells. **b** GFP-tagged wild-type TDP-43 is mostly nuclear with only faint colocalization with endogenous ORF1p in cytoplasic foci of some unstressed cells (arrows, top panels). However, mutations to the TDP-43 NLS (K82/84A and A90V) and some ALS-associated mutations (for example, A315T) show colocalization with ORF1p in cytoplasmic granules. **c** Cherry tomato-tagged wild-type SOD1 protein is diffusely cytoplasmic (top), but some ALS mutants are present with ORF1p in cytoplasmic foci of 293T cells. **d** Endogenous TDP-43 strongly colocalizes with endogenous ORF1p in cytoplasmic granules of Na-arsenite stressed but not untreated 2102Ep cells. ORF1p foci are much increased in size in stressed cells. **e** Red fluorescent protein (RFP)-tagged hnRNPA1 colocalizes with ORF1-EGFP cytoplasmic granules in unstressed HEK 293T cells. **f**, **g** Cytoplasmic granules formed by exogenously expressed OPTN or ANG do not colocalize with ORF1-EGFP granules. **h**) ORF1p is generally excluded from GFP-(GA)_31_ dipeptide aggregates. The full-length L1 construct pc-L1-1FH expresses ORF1p with HA-FLAG tags. Size bars are 10 μm

Previously, in a yeast two-hybrid screen we identified FUS protein as an ORF1p interaction partner, and we confirmed that the two wild-type proteins colocalized in cytoplasmic granules of a minor percentage of stressed human nTERA-2 embryonal carcinoma cells [60]. Here we generated in-house or obtained from other sources tagged constructs for selected ALS disease-associated mutants and transfected these in HEK 293T or 2102Ep cells in the presence of EGFP-tagged ORF1p or endogenous ORF1p alone. Various ALS-associated mutant but not wild-type FUS, TDP-43, and SOD1 proteins strongly colocalized with ORF1p in granules of unstressed cells (Fig. 4A-C). When cellular oxidative stress was induced by application of sodium arsenite, endogenous TDP-43 formed numerous cytoplasmic granules that strongly colocalized with endogenous ORF1p in most cells (Fig. 4D). Wild-type TDP-43 is known to form SGs in ALS neurons in response to cellular stress [119–121].

HNRNPA1 and HNRNPA2B1 are prion domain proteins that bind TDP-43 and have been linked with some ALS cases [122, 123]. Both proteins bind the L1 RNP, and HNRNPA1 colocalizes with ORF1 in SGs, as previously reported ([60, 124]; Fig. 4E). Wild-type TIA1, recently found mutated in cases of ALS and FTD [125], also strongly colocalizes with ORF1p in stressed cells as noted above (Fig. 1B). However, some ALS-related proteins, including OPTN and ANG (Fig. 4F, G), fail to colocalize in the same granules with ORF1p.

Expanded hexanucleotide repeats within transcripts of the *C9orf72* ALS gene can undergo non-conventional repeat-associated non-ATG (RAN) translation and generate dipeptide repeats that form inclusions in cerebellum, neocortex, and hippocampal neurons of C9 patients and toxic cytoplasmic aggregates in cultured neuronal cells or Drosophila models ([126–129], reviewed in [130]). To determine if these aggregates also colocalize with those of L1 ORF1p, we coexpressed in cultured cells a C9orf72 RAN translation product consisting of 50 GA repeats tagged with EGFP [131] and full length L1 with FLAG-HA-tagged ORF1. However, while overexpressed dipeptide proteins formed one to three large cytoplasmic aggregates in each cell, these did not colocalize with and generally excluded ORF1p (Fig. 4H).

Thus, a subset of RNA-binding proteins mutated in ALS bind and colocalize with L1 ORF1p RNP in cytoplasmic RNA granules.

### Overexpression of some ALS-associated proteins inhibit cell culture retrotransposition

**Fig. 5:**
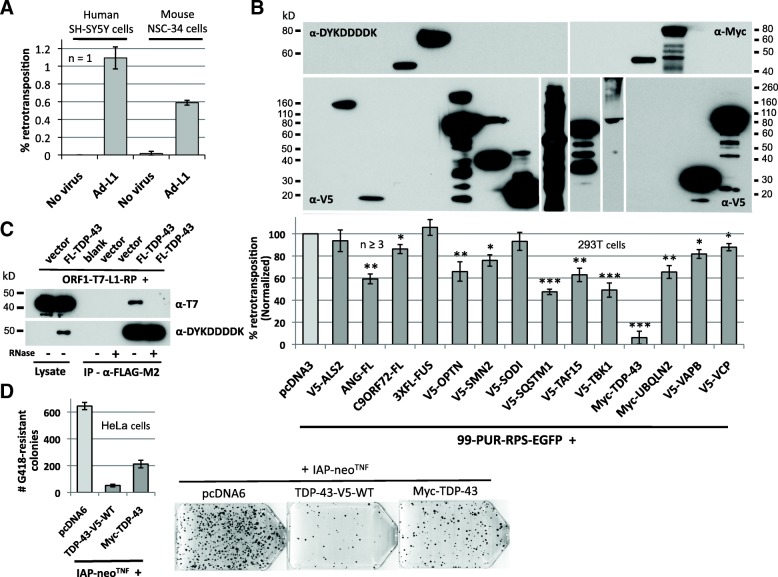
Increasing expression of some ALS-associated proteins alters retrotransposition in cell culture assays. n=number of biological replicates. **a** L1-RP is retrotransposition-competent in human SH-SY5Y neuroblastoma and mouse NSC-34 motor neuron-like cells. Cells in 6-well plates were infected with A/RT-pgk-L1RP-EGFP (Ad-L1) L1-reporter adenovirus [97] at about 8 × 10^12^ viral particles/ml. Flow cytometry analysis was performed at 9 days post-infection. **b** 99-PUR-RPS-EGFP was co-transfected in HEK 293T cells with empty vector (pcDNA3) or test constructs expressing tagged ALS-related proteins. Five days later, percentages of EGFP-positive cells were determined by flow cytometry. Each plasmid pair was transfected in four replicate wells, with at least 3 replicate experiments performed for each construct. Results are normalized to pcDNA3 vector control (lighter bar). Statistical significances compared with vector control were calculated by Student’s t-test (* p<0.05; ** p<0.01; *** p<0.001). All test proteins were expressed as confirmed by Western blotting of whole cell lysates using α-DYKDDDDK (FLAG)-tag, α-Myc-tag, or α-V5-tag antibodies as indicated (top). Four-fold less V5-SQSTM1- and three-fold more V5-TBK1-transfected lysates were loaded on the gel. Full-length TBK1 expressed poorly, most of the protein existing as a high molecular weight smear. **c** FLAG-tagged TDP-43 co-immunoprecipitates T7-tagged L1 ORF1p complexes from HEK 293T cell lysates after α-FLAG-M2 affinity gel purification. Interaction is lost following treatment of lysates with RNase. **d** Expression of V5- or Myc-tagged TDP-43 strongly inhibits mouse IAP element retrotransposition in HeLa-JVM cell culture. Cells were treated with neomycin (G418) to select for retrotransposition events. Colony counts are not normalized. On the right are representative T_75_ flask images with Giemsa-stained IAP retrotransposition-positive colonies in the absence or presence of TDP-43. The apparent diminished effect on retrotransposition of Myc-TDP-43 compared with TDP-43-V5-WT is likely because its plasmid backbone does not replicate and is diluted out of cells during the course of antibiotic selection which spans a couple of weeks

We previously showed that retrotransposition occurs in non-dividing mature neurons [23]. Here we extended these analyses and tested retrotransposition in two cell lines often used to study neurodegeneration, human SH-SY5Y neuroblastoma and mouse NSC-34 neuronal cells, the latter a hybrid line generated by fusing spinal cord motor neurons with neuroblastoma cells (Fig. 5A). Due to inefficient plasmid transfection, we infected cells with the adenovirus-retrotransposon hybrid virus, A/RT-pgk-L1RP-EGFP (Ad-L1) [23, 97]. This viral construct contains L1-RP tagged with the EGFP retrotransposition reporter cassette. Retrotransposition detected by flow cytometry was 1.1% and 0.6% of gated SH-SY5Y and NSC-34 cells, respectively. Thus, both primary and transformed neuronal cell lines are competent for retrotransposition.

We next asked if ALS-related proteins alter L1 retrotransposition in the cell culture assay described above [84, 87]. Briefly, we transfected HEK 293T cells with the retrotransposition reporter construct 99-PUR-RPS-EGFP together with constructs expressing tagged ALS-related proteins. 99-PUR-RPS-EGFP includes full-length L1-RP with the EGFP reporter cassette in its 3' UTR cloned in a modified version of pCEP4 vector (Invitrogen) lacking a cytomegalovirus (CMV) promoter. All constructs were expressed in HEK 293T cells (Fig. 5B, top). At least 3 biological replicates were performed. Test proteins did not cause significant cell death during the course of the experiment as determined by trypan blue exclusion staining (Additional file 1: Figure S4A). Three out of 14 proteins tested, including SQSTM1, TDP-43, and TBK1 kinase, reduced cell culture retrotransposition 50% or more when compared with cells transfected with empty vector only as control (Fig. 5B, bottom). SQSTM1/P62 is an autophagy receptor that targets bound proteins for selective degradation. Autophagy has previously been linked with retrotransposon restriction, and it was shown that SQSTM1 colocalizes with L1 RNA in stress granules, and that its knockdown causes increased accumulation of L1 and Alu RNAs and genomic insertions in cultured cells [71]. Autophagy misregulation has also been linked with numerous neurodegenerative disorders, including ALS.

TDP-43 is a multifunctional RNA-binding protein with roles in mRNA transcription, translation, transport, splicing, and stability [132–134]. Studies in model organisms have shown that overexpression of wild-type TDP-43 mimics loss-of-function phenotypes of neurodegeneration and motor dysfunction [135, 136]. Several other studies have considered how endogenous TDP-43 levels affect expression of TEs but with inconsistent results ([49, 51, 52, 137–139]; see discussion). In the HEK 293T cell culture retrotransposition assay, ectopic expression of TDP-43 with an N-terminal Myc-tag inhibits L1 retrotransposition over 90% (Fig. 5B). As this was the ALS-related gene that most altered retrotransposition levels, we next sought to characterize TDP-43 effect on L1 activity in more detail. To determine if ORF1p and TDP-43 interact, we co-expressed a construct containing L1-RP with T7-tagged ORF1 (ORF1-T7-L1RP) and TDP-43 with a C-terminal FLAG-tag: the two proteins co-immunoprecipitated on α-FLAG agarose (Fig. 5C). This association was RNA-dependent and was lost upon treatment with RNase, similar to almost all other proteins previously identified within the L1 ORF1p RNP [73, 124, 140, 141].

Over-expression of TDP-43 is toxic to neurons and cell toxicity has been associated with increased cytoplasmic mislocalization of some TDP-43 mutant proteins [142, 143]. We therefore thoroughly tested for TDP-43-induced toxicity of HeLa or HEK 293T cells by three methods: 1) comparison of the effect of TDP-43 overexpression on constitutive expression of antibiotic resistance in HeLa cells (Additional file 1: Figure S4B, C), 2) trypan blue staining for cell viability in HEK 293T cells (Additional file 1: Figure S4D), and 3) MultiTox-Fluor Multiplex Cytotoxicity Assay kit (Promega) analysis in HEK 293T cells (Additional file 1: Figure S4E). Overexpression of TDP-43 had no significant effect on cell viability during the time course of our assays, indicating the drop in retrotransposition efficiency is not a reflection of cellular toxicity.

We next tested if overexpression of TDP-43 might also inhibit the mobilization of LTR TEs. Human endogenous retroviruses are thought to be incapable of replication due to the presence of inactivating mutations in their ORFs [4]. However, mouse intracisternal A particle (IAP) LTR retrotransposons actively replicate and cause new mutations by insertional mutagenesis. Using an established cell culture assay [144], we found that in HeLa cells overexpression of C-terminal V5- or N-terminal Myc-tagged TDP-43 strongly restricted retrotransposition of an IAP element tagged with a neomycin phosphotransferase reporter cassette (Fig. 5D).

In a reciprocal assay, we next asked whether loss of endogenous TDP-43 affects L1 cell culture retrotransposition. We confirmed by Western blotting that two different siRNAs efficiently repressed endogenous TDP-43 protein when transfected in HEK 293T cells. However, TDP-43 depletion had no obvious effect on L1 retrotransposition, at least using the EGFP-based retrotransposition assay (Additional file 1: Figure S5A). We note that an inherent limitation of these assays is the transient nature of the siRNA-mediated protein depletion.

We also wondered if TDP-43 expression might affect the methylation status of the CpG island within the L1 5' UTR promoter [145]. We performed bisulfite conversion of genomic DNA from HEK 293T cells in which TDP-43 was either overexpressed (1 experiment; Additional file 1: Figure S6A) or depleted (2 independent experiments; Additional file 1: Figure S6B, C). PCR-amplified fragments containing the CpG island were cloned and at least 15 amplicons were sequenced for each sample [146]. Unexpectedly, when compared with controls, the 17 CpG residues of this region showed a significant overall increase in methylation in all experiments, although fully unmethylated sequences were found in all conditions. Therefore, one might speculate that perturbing steady-state TDP-43 protein levels alters DNA methylation status, a function for TDP-43 not to our knowledge previously reported. However, changes in L1 promoter methylation associated with TDP-43 expression were not accompanied by significant change in activity of either the L1 sense or antisense promoter in luciferase assays, at least using a plasmid-based assay (Additional file 1: Figure S6D). Moreover, TDP-43 overexpression failed to alter levels of endogenous or ectopically expressed ORF1p in cell culture, as determined by Western blotting (Additional file 1: Figure S5B, C), nor consistently affected levels of endogenous L1 RNA in HEK 293T cells as detected by RT-PCR (Additional file 1: Figure S5D).

**Fig. 6:**
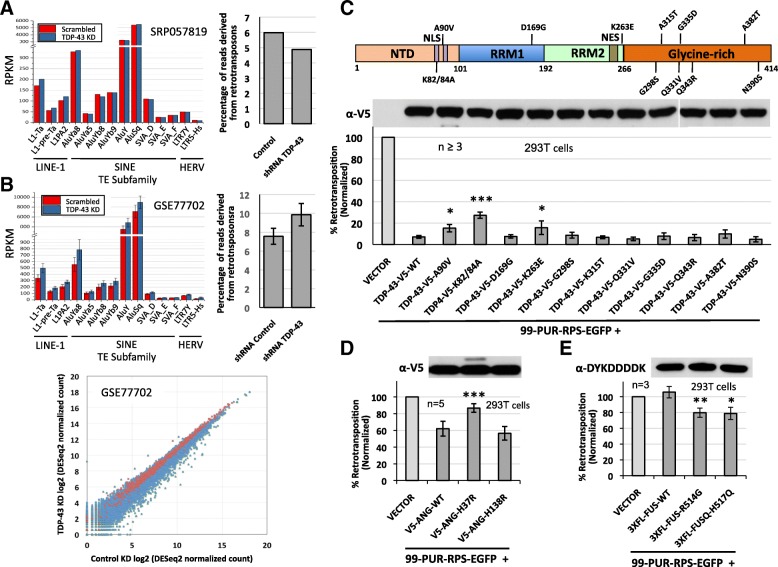
Effects of wild-type and mutant ALS-associated proteins on retrotransposon activity. **a** Plots of RPKM values for selected primate-specific non-LTR L1, Alu and SVA or LTR5_Hs (HERV-K(HML-2)) and LTR7Y (HERV-H) retrotransposon subfamilies in RNA-Seq data of control and TDP-43-depleted HeLa cells of study SRP057819 [147] (left). The percentage of reads originating from retrotransposons among total mapped reads (TEs and genes; right). **b** Plots of RPKM values for selected primate-specific retrotransposon subfamilies for human iPSC-derived motor neurons depleted or not depleted of TDP-43 by shRNA targeting from study GSE77702 [148]. Two replicate libraries were generated and sequenced in the GSE77702 dataset (left). The percentage of retrotransposon-related reads among total mappable reads (right). Scatter plot of the expression profiles of all genes (blue) and retrotransposon TEs (red) for the TDP-43 knockdown versus control motor neuron samples of GSE77702 (bottom). **c** Mutations within the nuclear localization signal (NLS) and RRM2 domains partially rescue inhibition of cell culture L1 retrotransposition by wild-type V5-tagged TDP-43. Top: the domain structure of TDP-43 showing the location of point mutations tested. NTD: N-terminal domain. n=number of biological replicates. **d** The ALS-associated ANG mutation H37R, but not H138R, significantly rescues cell culture L1 retrotransposition inhibition caused by expression of wild-type V5-ANG. **e** ALS-associated point mutations within the NLS of FLAG-tagged FUS significantly reduce cell culture retrotransposition compared with the wild-type protein. Statistical significance was calculated by Student’s t-test (*, p<0.05; **, p<0.01; ***, p<0.001)

To complement these analyses, we next re-analyzed two available RNA-Seq datasets not previously examined for TE expression (Fig. 6A, B). The first study (SRA SRP057819) generated single-replicate paired-end 100-bp RNA-Seq data of control and TDP-43-depleted HeLa cells (using the same esiTARDBP siRNA shown in Additional file 1: Figure S5A) [147]. The second dataset (GEO GSE77702) includes single-end 50-bp RNA-Seq data (two replicates each) of wild-type human iPSC-derived motor neurons depleted by shRNAs of TDP-43, TAF15, FUS, or combined TAF15-FUS [148]. FUS and TAF15 are both members of the FET family of RNA-binding proteins, which are linked with several neurodegenerative disorders [149]. In the HEK 293T cell culture retrotransposition assay, overexpressing FUS had no effect, while TAF15 reduced retrotransposition by 38% (Fig. 5B).

To detect changes in TE expression, we plotted RPKM (Reads Per Kilobase of transcripts per Million mapped reads) values for a subset of mostly evolutionarily young primate-specific non-LTR retrotransposons, including L1, Alu and SVA, and LTR5_Hs (HERV-K(HML-2)) and LTR7Y (HERV-H) subfamilies. Study SRP057819 showed an increase (approximately 15%) in RPKM values for the TDP-43 knockdown (KD) versus control HeLa cell lines for L1s only (including L1PA2 and human-specific L1-Ta and L1-pre-Ta subfamilies) (Fig. 6A, left). However, there was a slight overall decrease in the percentage of retrotransposon (LINE, SINE, SVA, and LTR)-related RNA-Seq reads among the total number of mappable (gene and TE) reads in TDP-43 KD cells (Fig. 6A, right). For TDP-43-depleted motor neurons of study GSE77702, there was a modest but consistent increase in RPKM values for all retrotransposon subfamilies when compared with scrambled shRNA control (Fig. 6B, left). In addition, there was a modest increase from 7.6 to 9.8% in the percentage of retrotransposon RNA-Seq reads among total mapped reads (TEs and genes) for TDP-43 KD versus control motor neurons (Fig. 6B, right). A scatter plot, however, showed minimal change in the expression profile of all mapped TE subfamilies versus genes for the GSE77702 dataset (Fig. 6B, bottom).

We then analyzed the GSE77702 dataset with TEtranscripts [150], a software package that uses short-read alignment files to identify differentially expressed (DE) TE subfamilies listed in RepBase, a database of representative repeat sequences in eukaryote genomes [151, 152]. A total of 192 retrotransposon subfamilies were expressed at significantly different levels in TDP-43 KD cells at an adjusted P-value (padj) <0.05 (Additional file 2: Table S1). However, only 55 retrotransposon TEs were significantly DE in the TDP-43 KD but in neither the TAF15 nor FUS KD datasets. Notably, only three DE retrotransposon TEs (HERVK3-int, MamGyp-int, and MER51D) were unique to TDP-43 KD cells and absent in the TAF15, FUS, and combined TAF15-FUS KD groups.

Therefore, and in contrast to some reports (see discussion), our analyses do not indicate a clear TDP-43-specific link with elevated activity of TEs, particularly LINE-1 retrotransposons. In fact, overexpression of wild-type TDP-43 strongly inhibits cell culture retrotransposition of both human L1 and mouse IAP elements.

### Mutation of some ALS-associated proteins alters cell culture retrotransposition

TDP-43 contains a NLS and NES, two RNA-recognition motifs (RRM1 and RRM2) that bind nucleic acids, and a C-terminal glycine-rich region that mediates protein interactions (Fig. 6C, top) [133]. A review in 2009 identified 70 pathogenic mutations in TDP-43, a majority in the glycine-rich domain [153]. We wished to determine if ALS-associated TDP-43 mutations might restore inhibition of retrotransposition by the wild-type protein, and so we tested the effects on L1 cell culture retrotransposition of a subset of mutant constructs. While all constructs expressed at levels similar to the wild-type, most mutations had no significant effect (Fig. 6C, bottom). However, a non-ALS TDP-43 double-point mutation in the N-terminal bipartite NLS (K82/84A) restored retrotransposition 3.5-fold (p<0.001). Similarly, ALS-associated NLS domain mutation A90V and RRM2 domain mutation K263E [154, 155] each rescued retrotransposition 2-fold (p<0.05).

We also considered the effect of angiogenin mutations on cell culture L1 retrotransposition. ANG is a member of the pancreatic RNase A superfamily and a potent mediator of neovascularization, as well as being a host defense factor against some microorganisms [156] and an enhancer of motor neuron survival [157, 158]. To date 33 *ANG* mutations have been implicated in ALS and Parkinson's disease [159]. Overexpression of V5-tagged ANG protein reduced cell culture retrotransposition to 62% of empty vector control without obvious cytotoxicity (Fig. 5B, 6D, S4C). We then introduced two disease-associated mutations known to abolish ANG RNase activity [159–161]. Notably, mutation H138R (H114R in the mature protein after signal peptide cleavage) had no effect, while H37R (H13R) restored retrotransposition to 87% when compared with vector-only control (Fig. 6D).

Similarly, we examined the effect of mutations in FUS protein. Exogenous expression of wild-type FUS had no effect on cell culture retrotransposition nor obvious cytotoxicity, but ALS-related mutations in its C-terminal NLS (R514G and H517Q) inhibited retrotransposition over 20% (Fig. 5B, 6E S4D, E). Finally we tested the effect of mutations in TBK1 on L1 retrotransposition. TBK1 is a member of the IKB kinase family, and an important player in innate immune signaling. Mutations in TBK1 also impair autophagy (153). Two mutants of TBK1, the kinase-dead mutant S172A and the ALS-associated mutant E696K (152) were tested, but neither showed any change from the 50% reduction of cell culture L1 retrotransposition caused by overexpression of the wild-type protein (Additional file 1: Figure S4F).

In sum, increased expression of some neurodegeneration-related proteins may decrease retrotransposon activity, while some disease-related mutations can modify these effects.

### Retrotransposon expression in tissues of ALS patients and controls

To further determine if changes in expression of non-LTR class retrotransposons are associated with ALS, we performed RT-qPCR analyses of 108 bulk spinal cord and brain tissue samples of 38 ALS patients and 27 non-affected controls (Additional file 3: Table S2) according to methods described in [146]. We assayed 30 thoracic or cervical spinal cord samples (15 ALS, 15 controls), 16 cerebellum (9 ALS, 7 controls), 35 motor cortex samples (23 ALS, 12 controls), 19 occipital cortex samples (14 ALS, 5 controls), and 8 hippocampal samples, all of the latter from ALS patients. Most samples were from sALS patients or patients of unknown etiology; only 5 patients had a known gene mutation. RT-qPCR primer pairs targeted the ORF1 and ORF2 regions of the young human-specific and retrotranspositionally active L1Hs subfamily (Additional file 1: Figure S7A, B) and two Alu subfamilies, AluS and AluY (Additional file 1: Figure S7C, D). Younger than AluJ elements, the AluS subfamily arose about 40 million years ago and may include some retrotransposition-competent elements [162, 163]. AluY, the youngest lineage, has the most retrotranspositionally active elements, and many genetic disorders in humans have been generated by AluY insertions [162, 164, 165]. Only L1Hs (L1P1)-type L1s are known to be retrotransposition-competent in the human genome [12, 166]. For the purposes of comparison, transcript levels were also determined for H9-hESCs [167]), human embryonic fibroblasts (HEFs), and HeLa cells.

Transcript levels were determined in duplicate for each brain and spinal cord sample, normalized to *GAPDH* internal control, and averaged. We considered all measurements of sample-specific transcript levels as real and did not omit possible outliers from analyses. Averaged RT-qPCR reactions within each experiment were normalized to expression of H9-hESCs as these cells strongly express endogenous L1 RNAs [168]; means and standard deviations are shown in Additional file 1: Figure S7. As previously observed, H9-hESCs expressed 5 to 25 times more L1 RNA than differentiated cultured cells such as HEFs or HeLa [146]. Expression levels of Alu and L1 element-related sequences detected in tissue samples were as high or higher than in H9 cells. Average expression in cerebellum was 2- to >3-fold higher than in other tissues for both Alu subfamilies and for L1s (except for ORF1 in occipital cortex); however, transcript levels in cerebellum and for L1 ORF1 in all brain tissue regions varied considerably between samples. Comparing expression of Alu and L1 elements in ALS versus unaffected controls, only expression of AluS elements in occipital cortex was significantly elevated for the 14 ALS versus 5 control samples (p=0.02) (Additional file 1: Figure S7C).

We next examined ORF1p expression by Western blotting of 60 brain and spinal cord tissue lysates (Additional file 1: Figure S8, Additional file 3: Table S2). There are very few studies of endogenous L1 protein expression in the brain. Baudin de The et al. [22] detected L1 ORF1p in ventral mid-brain tissues of mouse. Using a commercial antibody, Moszcynska et al. [169] showed by immunocytochemistry putative ORF2p expression in several rat brain regions, although antibody specificity was not assessed. Sur et al. [170] detected ORF1p by immunohistochemistry of various brain regions, and antibody detection by Western blotting was confirmed for a single frontal cortex sample. Here, using Western blotting and the α-4H1-ORF1 antibody, we were, surprisingly, unable to detect an ORF1p band of appropriate size in frontal cortex, cerebellum, or hippocampal brain tissue samples, and only very faintly in some motor cortex samples, even when 50 μg of whole cell lysate was loaded in a well (Additional file 1: Figure S8A-D) and despite the detection of L1-related RNAs expressed in these tissue types by RT-qPCR (Additional file 1: Figure S7). In contrast, we could detect a very robust full-length ORF1p signal from an equal amount of 2102Ep cell protein lysate (Additional file 1: Figure S8). Distinct bands consistent in size with full-length ORF1p were observed in some spinal cord samples; bands of smaller size were also seen, including a robust 38 kD signal of unknown origin (Additional file 1: Figure S8E). However, no overall differences in expression of ORF1p were evident in ALS compared with control spinal cord samples. Testing two different antibodies showed that failure to detect ORF1p signal in the brain was not limited to the α-4H1-ORF1 antibody (Additional file 1: Figure S8F, G).

In general, interpretation of TE expression from RT-qPCR data may be influenced by the presence of exonized TE-derived sequences in genes, the possible presence in the cell of non-integrated TE-derived cDNAs (see discussion below), and the cellular heterogeneity of the tissues analyzed. TE activation may occur in only a subset of cells within bulk tissue samples, so limiting sensitivity of detection, and in the case of motor neurons these cells may be progressively eliminated in the disease state. In sum, however, no major differences in TE expression where detected in ALS patients when compared with controls.

### Retrotransposon expression in ALS RNA-Seq datasets

Prudencio et al [171] generated a paired-end total RNA-Seq data set (GSE67196) from cerebellum and frontal cortex samples of 9 healthy, 8 *C9orf72*-associated ALS (C9ALS), and 10 sALS individuals and analyzed these for differentially expressed genes. A subsequent reanalysis of the same datasets using the HOMER analyzeRepeats program revealed significantly increased global expression of repetitive element types in frontal cortex but not cerebellum of C9ALS compared with sALS patients and healthy controls [52]. Setting FDR<0.1, the authors reported 300 DE TE subfamilies in the C9ALS samples: LTR class elements predominated (46%), followed by DNA elements (19%) and LINEs (18%). Notably, 91% of significant C9ALS DE repetitive elements had increased expression.

**Fig. 7:**
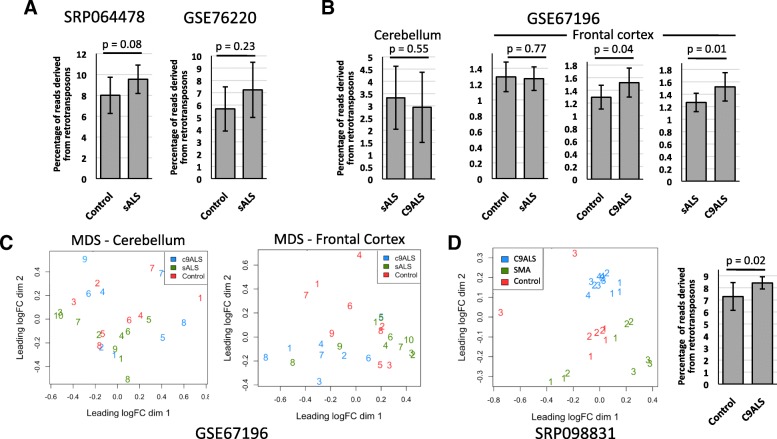
Analyses of RNA-Seq datasets for TE expression. **a** The percentage of retrotransposon reads among total mappable reads (TEs and genes) in the SRP064478 and GSE76220 RNA-Seq datasets. Significance was determined by Student's t-test. **b** The percentage of reads originating from retrotransposons among total mappable reads in cerebellum (left) and frontal cortex (right) samples of the GSE67196 dataset. **c** MDS plots of the GSE67196 dataset for TEs of C9ALS (blue), sALS (green), and control (red) samples. Samples are consecutively numbered for each group. **d** MDS plot of the NeuroLINCS SRP098831 dataset for TEs of C9ALS (blue), SMA (green), and control (red) samples (left). Individual patient samples are numbered consecutively and each replicate is given the same number. Discrete clustering of C9ALS samples is evident. Right: the percentage of retrotransposon reads among total mappable reads for C9ALS vs control samples of the NeuroLINCS dataset

We sought to assess further the degree to which TEs are differentially expressed in sALS-associated tissues by using TEtranscripts [150] to analyze two RNA-Seq datasets, SRP064478 and GSE76220, both publicly available in sequence read archives and neither previously examined for repeat expression (see also the Methods section). SRP064478 includes paired-end 150-bp sequence derived from total RNA of 7 sALS and 8 healthy control post-mortem cervical spinal cord samples. No significant difference in the percentage of averaged retrotransposon-derived reads among the total number of mappable reads (TEs and genes) was detected for ALS vs control samples; 1.72 to 3.41 million sample reads mapped to retrotransposon subfamilies (Fig. 7A, left; Additional file 2: Table S1). Only one significant DE retrotransposon subfamily was detected for SRP064478 (padj<0.05; Additional file 2: Table S1). However, overall mapping efficiency was low: about 25 million reads were alignable to the genome and 30% of these mapped to mitochondrial genes.

The GSE76220 dataset consists of single-end 50-bp sequence of total RNA isolated from laser-capture microdissected lumbar spinal cord sections of 13 sALS patients and 8 control individuals [172]. There was no significant change in the percentage of retrotransposon reads among total mappable reads in the sALS vs control samples (Fig. 7A, right; Additional file 2: Table S1). Between 0.27 and 1.26 million sample reads mapped to retrotransposons. TEtranscripts detected only four significant DE retrotransposon subfamilies (Additional file 2: Table S1).

We then reanalyzed the GSE67196 RNA-Seq dataset of Prudencio et al. [52, 171] using TEtranscripts. Significant increases in retrotransposon reads as a percentage of total mapped reads were seen for frontal cortex C9ALS vs control (p=0.04) and C9ALS vs sALS (p=0.01) samples (between 0.24 and 0.61 million sample reads mapped to retrotransposons) (Fig. 7B). As expected, multidimensional scaling (MDS) plots showed weak clustering of C9ALS samples in frontal cortex but not cerebellum samples (Fig. 7C). TEtranscripts detected no significant DE TEs in cerebellum samples of the GSE67196 dataset (padj<0.05). In the case of the frontal cortex samples however, and supporting Prudencio et al. [52], there were 3 DE TEs (DNAs, LTRs, LINEs, SINEs, and SVAs) in sALS vs controls, 10 DE TEs in C9ALS vs controls, and 133 DE TE subfamilies in C9ALS vs sALS samples, all increased in expression and including 36% LTR, 32% DNA, 15% LINE, and 17% SINE elements (Additional file 2: Table S1).

We also analyzed for the first time TE expression for the NeuroLINCS dbGaP Study phs001231 (SRP098831). This dataset consists of poly(A)+ non-stranded mRNA of iPSC-derived motor neurons from 4 C9ALS and 3 SMA patients (3 sequencing replicates each) and 3 unaffected controls (2 or 3 replicates each). Transcripts of some TE types are not polyadenylated and so are likely underrepresented in this dataset following poly(A)+ selection. However, although Alu elements are transcribed by RNA polymerase III and not polyadenylated, they contain both internal and 3'-end poly(A) stretches guaranteeing capture of their transcripts. An MDS plot showed C9ALS samples clustered away from SMA and control samples, while clustering of SMA from control samples was less evident (Fig. 7D, left). There was a significant increase (p=0.02) in the percentage of retrotransposon reads among total mappable reads in the C9ALS vs control dataset (Fig. 7D, right). The increase was also significant for C9ALS vs SMA (p=0.002) but not significant for SMA vs control samples (p=0.46) (not shown). TEtranscripts analysis showed that at padj<0.05, significant DE TE subfamilies (DNAs, LTRs, LINEs, SINEs, and SVAs) numbered 536 for C9ALS vs controls, 232 for C9ALS vs SMA, and 304 for SMA vs controls, most TEs being increased in expression (Additional file 2: Table S1). Three SVA and 30 Alu TEs were upregulated for C9ALS vs controls, including 6 AluY subfamilies. (The human-specific L1Hs/L1P1 subfamily was not detected). Interestingly, a recent literature review noted at least 37 neurological and neurodegenerative disorders linked with misregulated Alu retrotransposon activity [173]. A caveat of this dataset analysis is that sample numbers were small.

Algorithms such as Homer and TEtranscripts map sequencing reads to TE consensus sequences only and locus-specific information is lost. The ability to map individual transcribed retrotransposons to their source loci can reveal (i) the particular loci that contribute to repeat family transcription differences between diseased and healthy states, (ii) the coding capacity of transcribed repeat loci of possible relevance for a specific disease, and (iii) potentially variant retrotransposon proteins and RNAs that should be considered when studying disease relevance. We therefore applied a recently developed locus-specific TE mapping pipeline (PT, EP, DT, unpublished data), as described in the Methods section, to reanalyze the GSE67196 data set [171]. The numbers of mapped loci are summarized in Additional file 1: Figure S9A. Principal component analysis (PCA) and heatmap plots again showed cerebellum TE expression to be as variable within C9ALS, sALS, and control groups as between groups and without significant clustering (Additional file 1: Figure S9B, C, left). However, as expected, clustering of frontal cortex C9ALS samples distinct from control and sALS samples was evident (Additional file 1: Figure S9B, C right). Supporting Prudencio et al. [52], the greatest number of DE TE loci (determined as having padj<0.05 and greater than 2-fold differential transcription) were identified for C9ALS vs sALS (3963 loci), followed by C9ALS vs controls (652 loci), and sALS vs controls (109 loci). However, these DE TE loci comprised only 1.8%, 0.3%, and 0.06%, respectively, of a total of 2.12 x 10^5^ TE loci mapped (Additional file 4: Table S3).

Caveats of this type of analysis should be noted. For example, most of the significant DE L1 loci were likely not transcribed from their own promoters, since 1) almost 95% of those mapped were less than 5600 bp in length and so lacked much of their 5' UTRs, and 2) 70% of DE L1s were within genes, and so may be transcribed as part of a longer gene transcript. Moreover, only 2 younger primate-specific L1P1 and 4 L1PA2 L1 loci were differentially expressed (among a total of 164 L1P1 and 135 L1PA2 individual elements mapped). As for DE Alu loci, 81% were within genes (98% of all Alu loci being upregulated). Furthermore, most mapped Alu loci were older elements, with only 5% of them AluY subfamily members, a bias likely due to the inability of currently available algorithms to confidently map short sequence reads to young highly similar TEs. In general, designing RNA-Seq analysis pipelines that efficiently map short sequence reads of young highly similar TEs to their source loci has to date been difficult for reasons discussed below.

In summary, RNA-Seq analysis of the SRP098831 NeuroLINCs dataset suggests widespread upregulation of TE sequences from numerous subfamilies in *C9orf72* ALS patients, as previously reported for the GSE67196 dataset [52]. However, additional locus-specific analysis of the GSE67196 dataset suggests that many loci contributing mappable reads were not autonomously transcribed from their own promoters and were likely part of longer gene transcripts. More detailed transcription analyses targeting a selected cohort of full-length intact intergenic TE loci are needed to validate misregulation of retrotransposon expression in *C9orf72*-associated ALS disease.

## Discussion

Self-aggregation of RNA-binding proteins is a leitmotif of neurodegenerative diseases, including amyotrophic lateral sclerosis. The ORF1 protein of LINE-1 retrotransposons is also an aggregation-prone RNA-binding protein. Of the approximately 500,000 L1s in the human genome, about 5000 are full-length, or about one percent of DNA [10, 174]. Many of these L1s have the potential to be transcribed and translated, although different tissues may express different L1s [175]. We speculate that misregulation of even a small number of these, leading perhaps to mislocalization and augmented aggregation of ORF1p, could have negative effects on some cells, including neurons. In this study, to increase our understanding of the role of ORF1p in disease, we extended previous investigations of its subcellular localization and aggregation properties. We then considered potential interactions of amyotrophic lateral sclerosis-related proteins and the ORF1p RNP and the possibility of misregulated L1 activity in the ALS state.

Analogous to gene products associated with certain neurodegenerative diseases, L1 ORF1p RNPs are prone to forming cytoplasmic RNA granules. In unstressed cells of some lines, ORF1p constitutively forms cytoplasmic granules that are only faintly and partially marked by canonical stress granule proteins. As shown in Fig. 1, stress to the cell increases both the size of ORF1p cytoplasmic aggregates and colocalization with SG proteins, and deleting a Q-N-rich region of human ORF1p abolishes aggregation. Furthermore, we showed here that L1 elements with a variant ORF1 R159 codon, a residue that controls both retrotransposition and the ability of ORF1p to seed cytoplasmic RNA aggregates, are common in the human genome. Thus, cell stress promotes and certain sequence polymorphisms alter cytoplasmic aggregation of L1 ORF1p.

A functional role for L1-associated cytoplasmic RNA granules in retrotransposition remains unknown. This begs the question, what are these constitutively expressed ORF1p aggregates? ORF1p fails to associate with Golgi, lysosome, or endoplasmic reticulum marker proteins [60, 176, 177]. Endogenous ORF1p aggregates in unstressed cells occasionally abut P-bodies but generally do not overlap ([60]; Additional file 1: Figure S1F). Guo et al. [71] found that exogenous and endogenous ORF1p colocalized with autophagosome marker LC3 protein in HEK 293T cells, an association that increased with inhibition of autophagy. We here confirmed that endogenous ORF1p granules of unstressed 2012Ep cells are also partially marked by red fluorescent protein (RFP)-tagged LC3 (Additional file 1: Figure S1G), but we failed to detect their colocalization with endogenous autophagy marker proteins ATG12 or ATG16L1 (Additional file 1: Figure S1H, I). It has also been reported that ORF1p co-IPs and colocalizes in some cytoplasmic granules with IGF2BP1/IMP1 [124], part of a multi-protein complex found in granules of neuronal axons [178–180]. IMP1 granules have been reported as distinct from P-bodies and stress granules [181]. It is therefore possible that ORF1p aggregates in more than one type of cytoplasmic structure.

Endogenous ORF1p may concentrate as perinuclear, nuclear, or nucleolar. We showed in Fig. 2 that in some cells ORF1p also forms small nuclear foci distinct from nucleoli; ectopically expressed Alu and likely other RNAs colocalize with these foci. Other studies have also reported endogenous ORF1p nuclear localization, specifically in some human cancer cell lines and tissues [60, 76, 77, 79, 182], and murine germline, chloroleukemia, and cardiomyocyte cells [183–187]. Why ORF1p is cytoplasmic in some cells and nuclear in others is unclear but suggests a dynamic aspect of ORF1p biology that is starting to be appreciated in the retrotransposon field [100].

We therefore examined the cell cycle as one possible mechanism controlling ORF1p subcellular localization. Blocking 2102Ep cell cycling at G_1_/S phase transition did not obviously alter ORF1p nuclear localization. Non-blocked cells showed significant concentration of ORF1p in nucleoli, whether the cells were in G1 phase or not. Our results in part contradict a recent study proposing a strong cell cycle bias for ORF1p accumulation in the nucleus during mitosis where it remains during G1 phase [100]. This discrepancy may in part be due to the fact that we queried strictly nucleolar accumulation as the most obvious feature of ORF1p nuclear localization in 2102Ep cells; failure to observe ORF1p in nucleoli does not necessarily preclude its diffuse presence in the nucleoplasm. Also, the Mita et al. study [100] tested a different cell line (HeLa) and ORF1p overexpressed from plasmids, while here we examined endogenous ORF1p localization as more biologically relevant. Indeed, 2102Ep cells mimic early human embryogenesis, where heritable L1 insertions accumulate [69, 168], while HeLa cancer cells mimic L1 activity in human cancers. While both cellular niches are known to support L1 retrotransposition, it stands to reason that differences may exist with respect to L1 regulation. Thus, the mechanisms that control L1 ORF1p nuclear localization clearly require further investigation in a range of cell lines and with care paid to their growth conditions (for as noted above, the frequency of ORF1p nucleolar localization varies with 2102Ep cell density).

Previously, we showed that ORF1p point mutations and C-terminal and N-terminal deletions increased nuclear accumulation (see text and Supplemental data of [60]). Thus, maintaining the integrity of ORF1p structure seems to be important for cytoplasmic retention and aggregation. Furthermore, concentration of ORF1p at the nuclear membrane of some cells (Fig. 2), its reported RNA-dependent association with karyopherin subunit alpha 2 (KPNA2; [124]), and the detection by mass spectrometry of importin 7 (IPO7) within an ORF2p complex [141], suggest that L1 RNPs interact with the nuclear import machinery. Indeed, it was recently shown that loss of transportin 1 (TNPO1), the beta subunit of the karyopherin receptor complex, reduces nuclear localization of epitope-tagged ORF1p [188]. It was also recently proposed that ORF1p expression is required for nuclear ORF2p localization [141]. However, this is not supported by our earlier findings that ORF2p overexpressed alone efficiently enters nucleoli of human osteosarcoma 143B TK- cells; at that time we also mapped a functional nuclear localization signal to the N-terminus of ORF2p [76].

Might there be cellular consequences for misregulated expression or mislocalization of aggregation-prone LINE-1 proteins? L1 ORF1p is a promiscuous RNA-binding protein able to capture many cellular RNAs. Co-IP experiments with tagged L1 RNPs have identified numerous bound RNAs, including SVA and Alu SINEs and small non-coding RNAs of importance for the cell [124, 189]. Direct co-IP experiments also confirmed over 60 proteins that associate with tagged L1 ORF1p RNPs, mostly in an RNA-dependent manner [60, 73, 124, 190]. Among these were several RNA-binding proteins associated with ALS and FTLD, including FUS, HNRNPA1, HNRNPA2B1, and TDP-43. As we have shown here, pathogenic mutants of ALS proteins FUS, SOD, and TDP-43 also colocalize with ORF1p in cytoplasmic RNP aggregates. As with certain neurodegeneration-associated proteins, increased expression or mislocalization of ORF1p, whether through mutation or loss of L1 suppression, could seed protein aggregation, co-sequester other cellular proteins or RNAs, disrupt normal patterns of protein degradation or RNA processing, and trigger cytotoxicity. Retrotransposon-encoded proteins can also induce cellular stress responses. Gasior et al. [191] showed that overexpression of L1 ORF2p causes double-strand chromosome breaks. These results are consistent with observations that L1 overexpression can induce apoptosis and senescence or potentially an immune response in some cell lines [192–195]. Perhaps these are reasons for the evolution of so many cellular factors that restrict L1 activity [42].

Previous studies have considered links between TDP-43 and retrotransposon expression. TDP-43 was first identified as a transcriptional repressor that binds the RNA regulatory element TAR of HIV-1 proviruses to inhibit their expression [196]. However, a role for TDP-43 in regulating HIV or HERV transcription is not clear [197]. Douville et al. [49] found expression of HERV-K(HML2) *pol* and *TARDBP* genes to be strongly and positively correlated, and their encoded proteins colocalized in ALS neurons. Douville and Nath [198] also linked TDP-43 with altered HERV-K(HML-2) RT expression in brain tissues. Data-mining rodent and human interaction experiments, Li et al. [137] found that TDP-43 protein targets and binds LTR and non-LTR TE transcripts and that this association is reduced in cortical tissues of FTLD patients. Furthermore, reanalysis of RNA-Seq datasets of human TDP-43 overexpressed in transgenic mice [199] and endogenous TDP-43 depleted in mouse striatum [200] showed a general increase in expression of LTR, non-LTR and DNA TEs under both conditions, with concordance between TE transcripts upregulated and those bound by TDP-43 protein [137]. While it was reported [51] that TDP-43 protein bound the HERV-K LTR with an attendant increase in HERV-K(HML-2) transcription and RT activity, Manghera et al. [138] found wild-type TDP-43 bound the HERV-K(HML-2) promoter without activating its transcription, while overexpressed ALS-associated TDP-43 mutants promoted HERV-K(HML-2) protein aggregation and clearance from astrocytes (but not neurons) by stress granule formation and autophagy. Overexpression of a human TDP-43 transgene in Drosophila was accompanied by motor problems and derepression of retrotransposons in general and glial cell-specific upregulation of gypsy elements in particular, along with an increase in programmed cell death induced by DNA-damage [139].

We found that, while TDP-43 binds and colocalizes with the L1 ORF1p RNP, its increased expression strongly represses rather than derepresses human L1 and mouse IAP cell culture retrotransposition (without attendant cytotoxicity). On the other hand, inhibition of endogenous TDP-43 had no effect on L1 retrotransposition in HEK 293T cells. Although altered levels of TDP-43 were associated with modestly increased methylation of endogenous L1 promoters, this was not accompanied by a change in exogenous ORF1p expression or promoter effects in a luciferase assay. Moreover, a recent study by Prudencio et al. [52] found no significant association between levels of TDP-43 RNA or protein and TE expression in frontal cortex samples of a large cohort of ALS/FTLD patients. Our reanalysis of two RNA-Seq datasets [147, 148] also failed to detect strong TDP-43-specific changes in expression of retroelement subfamilies in cell lines depleted of endogenous TDP-43 protein. Therefore, a role for TDP-43 protein in aberrant retroelement activity begs further investigation.

We also reanalyzed for TE subfamily expression two RNA-Seq datasets of sALS tissue samples not previously examined for TE expression (GSE76220 and SRP064478) and one previously tested dataset of both *C9orf72* and sporadic ALS tissue samples (GSE67196). In all three datasets, we failed to find significant misregulation of TE subfamilies in sALS vs controls, consistent with the previous analysis of GSE67196 [52] and with our RT-qPCR and Western blot analyses of ALS and control brain and spinal cord tissues. However, our analysis of a NeuroLINCs dataset (SRP098831) found both SMA and C9ALS vs non-ALS patient-derived iPSC cell lines differentiated to motor neurons to have significant numbers of DE TEs, including young SINE Alu subfamilies: this was in line with the previous findings of Prudencio et al. [52] that TE expression is misregulated in C9ALS vs sALS samples of the GSE67196 dataset. However, our locus-specific analysis of the GSE67196 dataset suggested that many of the reads contributing to the retrotransposon subfamily analyses did not originate from TE sequences transcribed from their own endogenous promoters but rather from sequences contained within longer transcripts.

Several pitfalls exist for RNA-Seq analyses of differential TE expression: conclusions should be drawn with care. High copy number, close sequence similarity, and especially the frequent embedment of TE sequences in longer gene transcripts (i.e., exonization) can lead to misinterpretation. While expression of a TE subfamily may appear misregulated, a change in expression observed may in fact be due to altered expression of a gene in which a member of that TE subfamily resides. In their analysis of RNA-Seq data from HEK 293T cells, for example, Deininger et al. [174] mapped greater than 99 percent of L1-derived sequence reads within other RNAs unrelated to retrotransposition. Moreover, *bona fide* L1 transcripts originating from L1 5' UTR promoters were limited to only a small number of highly expressed full-length L1 loci.

Furthermore, we have speculated that cell conditions that induce elevated expression of L1s or HERVs, and therefore their encoded reverse transcriptases, could induce promiscuous reverse transcription of cytoplasmic RNAs ([42], see also [201]). Indeed, a recent report has demonstrated the accumulation of cytoplasmic L1-related ssDNAs in neurons derived from hESCs lacking the exonuclease TREX1, a gene mutated in Aicardi-Goutières syndrome patients [195, 202]. The ectopic cytoplasmic cDNAs so generated would be amenable to amplification during RNA-Seq or RT-qPCR protocols and so bias upwards estimates of expression from their source loci. Although as yet an unverified concern, recent studies have reported elevated levels of TE-derived cytoplasmic cDNAs or hybrid RNA/DNA molecules in cancer and other disease states [195, 201, 203]. The retrotransposon field is working to control such possible sources of error when interpreting TE expression data [174, 175]. In general, further improvements in transcriptomics, and especially single-cell based approaches, will eventually clarify the degree of deregulation of TE expression in ALS and other neurodegenerative disorders.

## Conclusions

In considering links between retrotransposon expression and neurodegenerative conditions, we expanded previous knowledge of the aggregation properties of the LINE-1-encoded ORF1 protein and factors that control its accumulation. We also presented data that the cell cycle does not strongly alter nuclear localization of endogenous L1 ORF1p in nullipotent embryonal carcinoma cells. We showed that some ALS-associated protein mutants associate with ORF1p in cytoplasmic aggregates and that increased expression of some ALS-linked proteins limit LINE-1 retrotransposition. We emphasized especially TDP-43, a protein that accumulates in the cytoplasm of a majority of ALS patients, but failed to find consistent evidence in cell culture for an effect on retrotransposon activity, in contrast to some previous reports.

By means of RT-qPCR and Western blotting of ALS tissues and reanalysis of available RNA-Seq datasets, we also sought a link between sporadic ALS and retrotransposon misregulation. In sum, clear-cut evidence is so far lacking for involvement of non-LTR retrotransposon expression in sALS. Using the same tissue samples as in the present study, we also recently profiled transcription of HERV-K(HML-2) and HERV-W LTR retrotransposons by direct Sanger sequencing of cloned cDNAs and RT-qPCR and Western blot analyses, but failed to find significant differences when comparing ALS and controls [204]. It is conceivable that previous observations of differential TE expression levels may relate to altered global DNA methylation status and other epigenetic changes observed in some ALS patients [205–208], which could in consequence cause selected TE loci to be differentially transcribed. At least, analyses of additional *C9orf72*-mutated ALS RNA-Seq datasets seem warranted. We believe examining neurodegenerative disease-affected tissues for perturbations in the aggregation dynamics of L1-encoded proteins could also prove informative. It is also reasonable to continue to apply improving methods of next-generation sequencing analysis to examine neurodegenerative and other brain diseases for misregulated activity of TEs in general and the L1 in particular, a mobile element with hundreds of thousands of copies and which through long evolution has been directly responsible for generating over a quarter of the DNA in the human genome [209].

## Methods

### Plasmid and RNAi constructs

Plasmid constructs were kindly provided by the following researchers: 3xFL-FUS-WT, 3xFL-FUSL-R525L, 3xFL-FUSH-R514G, 3xFL-FUSQ-H517Q, and 3xFL-FUSQ-R521H (J. Manley, Columbia University; [210]); pcDNA5 FRT/TO (G4C2)_31_ (M. Cozzolino, University of Rome "Tor Vergata"; [96]); GFP-(GA)_50_ (L. Petrucelli, Mayo Clinic, Florida; [131]); IAP-neo^TNF^ (M. Dewannieux, Institut Gustave Roussy; [144]); pmRFP-LC4 (T. Yoshimori, Addgene 21075); pEGFP-C1-MK2-mut T205/317E (M. Gaestel, Medical School Hannover, Germany; [111]); pcDNA3 3XFL-OPTN-E50K (H. Kawakami, Hiroshima University; [211]), mCherry-PSP1 (D. Spector, Cold Spring Harbor Laboratory; [212]); EGFP-TDP-43-WT, EGFP-TDP-43-A315T, EGFP-TDP-43-K82/84A (B. Wolozin, Boston University; [213]); pRK5-Myc-TDP-43 (J. Wang, Johns Hopkins University, [214]); SOD1 WT-Tomato, SOD1 A4V-Tomato, SOD1 G93A-Tomato (J. Yerbury, University of Wollongong; [215]), pCS2 (+)MT UBQLN2 WT2 (Myc-UBQLN2), and pCS2 (+)MT UBQLN2 P497H (D. Ito, Keio University School of Medicine; [216]). The following plasmids have been previously described: 99-PUR-RPS-EGFP, 99-PUR-JM111-EGFP [87], ORF1-T7-L1RP, ORF1p-EGFP, HNRNPA1-RFP [60], ORF1-EGFP-L1-RP, pBS 7SL Alu-MS2 (Ya5), pcDNA SVA_SPTA1_-MS2, ORF1-WT-GFP [72], and pc-L1-1FH [124].

Ultimate ORF cDNA clones (Invitrogen) were cloned with V5-epitope tags and tobacco etch virus (TEV) protease cleavage sites on their N-termini by shuttling them from pENTR221 vector into pcDNA3.1/nV5-DEST vector using Gateway Technology (Invitrogen). Ultimate ORF Clone ID numbers were V5-ALS2 (IOH62502), V5-ANG (IOH29453), V5-OPTN (IOH57143), V5-SMN2 (IOH10903), V5-SOD1 (IOH4089), V5-SQSTM1 (IOH5103), V5-TAF15 (IOH40855), V5-TBK1 (IOH21006), V5-VAPB (IOH4934), and V5-VCP (IOH52832). FL-TDP-43 was generated using Ultimate ORF Clone IOH45677 and Gateway vector pEZYflag (Y.-Z. Zhang, Addgene 18700). TDP-43-V5-WT and TDP-43-FL were generated by PCR-amplification of TDP-43 from pRK5-Myc-TDP-43 with AAG linker and C-terminal V5- and FLAG-tags, respectively, and cloned in pcDNA6/myc-His B (pcDNA6, Invitrogen). C9ORF72-FL with AAG linker and C-terminal FLAG tag was amplified from Ultimate ORF Clone IOH45695 and cloned in pcDNA6/myc-His B.

The helper-dependent adenovirus for construct A/RT-pgk-L1RP-EGFP (Ad-L1) [97, 217] was prepared as described [218]. N1-EGFP was from Clontech. The pc6-RPS-EGFP-ΔCMV retrotransposition reporter construct included full-length L1-RP and the EGFP reporter cassette in a modified version of pcDNA6/myc-His B vector lacking a cytomegalovirus (CMV) promoter. PCR and QuickChange mutagenesis methods were used to generate ORF1-EGFP-L1-RP-R159H (from ORF1-EGFP-L1-RP), ORF1-EGFP-L1-RP-LKEAAAAAL, ORF1p-Δ179-205-GFP (from ORF1p-EGFP), LKELLKEAL-EGFP (from N1-EGFP), V5-ANG-H37R and V5-ANG-H138R (from V5-ANG), V5-TBK1-S172A and V5-TBK1-E696K (from V5-TBK1), and pc6-RPS-EGFP-ΔCMV-R159H (from pc6-RPS-EGFP-ΔCMV).

siRNAs (Additional file 1: Figure S5A) were synthesized or purchased from Sigma-Aldrich:

siCNT2: CUCCCGUGAAUUGGAAUCC[dT][dT]

siCNT3: AUGUAUUGGCCUGUAUUAG[dT][dT]

siCNT4: UAAGGCUAUGAAGAGAUAC[dT][dT].

2siTDP43: AUAUCCAUUAUGCACCACC[dT][dT]

esiTARDBP: EHU109221 MISSION esiRNA.

### Cell culture and tissues

Human 2102Ep embryonal carcinoma cells (a gift from P.K. Andrews, University of Sheffield), human cervical cancer HeLa-JVM cells [219], human embryonic kidney (HEK) 293T cells (ATCC), human embryonic fibroblasts (HEFs, ATCC), and mouse hybrid motor neuron NSC-34 cells (a gift from D. Griffen, JHU) were grown in Dulbecco’s modified Eagle’s medium (DMEM). SH-SY5Y cells (ATCC CRL-2266) were grown in DMEM/F12 (Ham) medium (Gibco) and human SK-N-SH cells (a gift from D. Valle, Johns Hopkins University) were grown in Eagle's Minimum Essential Medium. Medium was supplemented with 10% FBS (Hyclone or Sigma), GlutaMax, and Pen-Strep (Invitrogen). Plasmid and siRNA transfections used FuGENE HD (Promega) or Lipofectamine 3000 (Thermo Fisher Scientific) reagents. H9 human ESCs [167] were obtained from Wicell (RRID: CVCL_9773) and cultured and passaged as previously described [168].

Post-mortem brain and spinal cord frozen tissues were obtained from the University of Maryland Brain and Tissue Bank of the NIH NeuroBioBank, the Target ALS Multicenter Postmortem Tissue Core at Johns Hopkins University, and the Department of Neurosciences of the University of California San Diego School of Medicine, as indicated in Additional file 3: Table S2. All tissues were obtained following approval of the Institutional Review Boards of the UCSD School of Medicine (to JR) and the JHU School of Medicine (IRB00066246 to JLG).

### Immunofluorescence and microscopy

Commercial antibodies included rabbit (rb) α-ATG12 (D88H11), rb α-ATG16L1 (D605), rb α-CDT1 (D10F11), rb α-cyclin B1, mouse (ms) α-DYKDDDDK (FLAG)-tag (9A3) (all Cell Signaling Technology), goat (gt) α-eIF3η (N-20, Santa Cruz), rb α-Geminin (Cell Signaling Technology or ab195047, Abcam), ms α-p70 S6 kinase (which recognizes HEDLS/EDC4; [220]) (H-9, Santa Cruz), rb α-Myc-tag (71D10, Cell Signaling Technology), ms α-TDP-43 (10782-2.AP, Proteintech), gt α-TIA1 (C-20, Santa Cruz), ms α-T7-Tag (Novagen), ms α-TLS (FUS) (BD Transduction Laboratories), rb α-β-tubulin-2 (Pierce), and ms α-V5-tag (Invitrogen). Purified ORF1p antibodies included rb polyclonal α-ORF1p-AH40.1 (4292) (a gift from M. Singer, Carnegie Institute for Science; [68]), rb monoclonal α-JH73-ORF1 (from J. Han, Tulane University and K. Burns, Johns Hopkins University; [177]), rb polyclonal α-V14-ORF1 (a gift from C. Harris, The Verto Institute; [182]) and ms α-4H1-ORF1 (from K. Burns, and Millipore MABC1152; [67]). Human α-ANA-N was obtained from a patient with autoimmune disease [76]. Donkey Cy3-, DyLight 488-, DyLight 549-, Alexa Fluor 594-, and peroxidase-conjugated secondary antibodies were from Jackson ImmunoResearch Laboratories.

Western blotting, IF, and RNA FISH were performed as described [60, 72]. All Western blots were run on NuPAGE 4-12% Bis-Tris gels (ThermoFisher). Cells were examined using a Nikon Eclipse Ti-A1 confocal microscope with NIS-Elements AR software.

### Whole-cell protein and RNA extraction

For protein extracts, tissues or cells were lysed in RIPA buffer (Sigma) with Mammalian Protease Inhibitor Cocktail and phenylmethanesulfonyl fluoride (Sigma) and homogenized with a Diagenode Bioruptor. In the case of tissues, 2 mm zicronium silicate beads (Next Advance) were added to the tubes. Samples were centrifuged at 11K at 4^o^C for 15 minutes to recover supernatant and resuspended in 3X SDS loading buffer. Isolation of HEK 293T cell nuclear and cytoplasmic extracts utilized the NE-PER kit (Thermo Scientific).

For RNA extracts, all brain tissue and some spinal cord tissues were disrupted and homogenized in 500 ml of Trizol (Invitrogen) using the TissueLyser LT (Qiagen). Briefly, 30 mg of sample were transferred to a 2 ml tube containing 250 μl of Trizol and one 5 mm stainless steel bead. The TissueLyser LT program used was 50Hz for 1 min. After a spin, the supernatant was collected and another 250 μl were added to the sample to repeat the same procedure. Finally, both fractions were combined and RNA purification with Trizol followed the manufacturer`s instructions. Some spinal cord samples were homogenized in 500 μl of Trizol and zicronium silicate beads using a Benchmark BeadBlaster24. Following centrifugation, the supernatant was further purified using an RNeasy Mini Kit with On-column DNase digestion with RNase-Free DNase Set (Qiagen).

Next, the RNA was treated with RQ1 RNase-free DNAse (Promega) for 30 min, purified with ultrapure phenol:chloroform:isoamyl alcohol mixed at 25:24:1 (v/v/v) (Ambion) and precipitated with 3 volumes of ice cold 100% ethanol and 0.1 volume 3M sodium acetate. To assure absence of cross-contaminating genomic DNA, 1 μg of total RNA was treated again with another round of RNase-free DNase I (Invitrogen) for 15 min.

RNA Integrity numbers (RINs) are shown in Additional file 3: Table S2 (range: 2.1-10; median 6.6). RNA integrity numbers (RINs) were determined using an Agilent BioAnalyzer and Agilent RNA 6000 Nano Kit following the manufacturer's recommendations. We attribute low RIN numbers in some samples to long post-mortem intervals affecting tissue quality and to the rigorous DNase-treatments of RNA that were required to remove residual contaminating genomic DNA, a strategy necessary for our sensitive PCR amplification of multi-copy repeat cDNAs. To assess effects of RNA quality on our analyses we plotted RIN values versus RT-qPCR Ct-values of *GAPDH* and could detect no significant effect of RIN when the various tissue types were considered separately. However, a mild effect (R^2^=0.38) of RNA quality on Ct-levels is acknowledged when combining RIN and Ct values from all samples. Importantly, omission of samples with lower RINs did not affect our conclusions.

### Retrotransposition assay

The EGFP L1 cell culture retrotransposition assay was conducted as previously described [87, 221, 222]. The IAP retrotransposition assay was carried out essentially as described in [144]. One μg of IAP-neo^TNF^ element reporter plasmid was cotransfected with 0.5 μg of empty vector or test plasmid in HeLa-JVM cells. At eighteen hours post-transfection, the cells were expanded from six-well plates to T_75_ flasks, and two days later selection for retrotransposition events with 500 μg/ml of G418 was begun. After 15 days of selection, cells were fixed, stained with Giemsa, and colonies were counted.

SH-SY5Y and NSC-34 cells were infected with Ad-L1 at ∼8 × 10^12^ viral particles/mL [97, 217].

### Bisulfite experiments

Bisulfite analysis was performed as described [21, 146] using the EZ DNA Methylation Gold Kit (Zymo Research). The analyses of Additional file 1: Figure S2 queried by PCR the methylation status of 9 CpGs within a 436-nt stretch (1169-1604) of L1 ORF1 surrounding the R159 polymorphism and used primers 1ORF1-R159-BisulfFOR (AGGAGTTGATGGAGTTGAAAATTAAG) and 2ORF1-R159-BisulfREV

(GACCTTTCTCTCTAACTACCCTTAAC). PCR amplicons analyzed for Additional file 1: Figure S6 spanned 363 nts of the L1 5' UTR containing 17 CpG dinucleotides; primers were For (AAGGGGTTAGGGAGTTTTTTT) and Rev (TATCTATACCCTACCCCCAAAA). PCR products were subcloned (TOPO TA Cloning Kit, Invitrogen), sequenced, and analyzed with the QUantification tool for Methylation Analysis, QUMA (quma.cdb.riken.jp; [223]). The significance of methylation differences was examined with Fisher's Exact Test statistics generated by QUMA.

### Assessment of toxicity

To test potential protein toxicity (Additional file 1: Figure S4), we co-transfected in HeLa-JVM cells pcDNA6/myc-His B, a blasticidin S-resistance gene (bsr)-containing vector, together with empty vector (pcDNA3) or test expression constructs. On day 2, cells were expanded to T_75_ flasks and selection with 5 μg/ml blasticidin was begun. After 12 days, cells were fixed, stained with Giemsa and colonies were counted. Similarly, we co-transfected in HeLa cells pcDNA3, a neomycin (neo)-resistant vector, together with either empty vector (pcDNA6/myc-His B) or test expression constructs, followed by selection of cells with 500 μg/ml Geneticin (G418, Thermo Fisher).

Trypan Blue exclusion assays were performed in HEK 293T cells. Following staining, live and dead cells were counted using a Countess II Automated Cell Counter (Thermo Fisher Scientific). Use of the MultiTox-Fluor Multiplex Cytotoxicity Assay kit (Promega) followed manufacturer's instructions. This assay simultaneously measures cell viability and cytotoxicity in a single-reagent reaction, permitting ratios of live to dead cell readings to be calculated.

### RT-qPCR

RT-qPCRs were conducted as previously described [146, 224]. A High-Capacity cDNA Reverse Transcription Kit (Applied Biosystems) was used to generate cDNA. RT-negative controls were run in parallel for all qPCR reactions. Duplicate samples were analyzed in a StepOne Real-Time PCR system (Applied Biosystems) using GoTaq qPCR Master Mix (Promega) and PCR primers at 200 nM each. We used two sets of primers to analyze endogenous L1 expression directed against L1Hs ORF1 (N-51-Fwd: GAATGATTTTGACGAGCTGAGAGAA; N-51-Rev: GTCCTCCCGTAGCTCAGAGTAATT) or L1Hs ORF2 sequence (N-22 Fwd: CAAACACCGCATATTCTCACTCA; N-22 Rev: CTTCCTGTGTCCATGTGATCTC). We also analyzed expression of AluS (AluS-Fwd: GCCGAGGCGGGCGGATCACC; AluS-Rev: GCCTCCCGAGTAGCTGGGAT) and AluY (AluY-Fwd: AGATCGAGACCATCCTGGCT; AluY-Rev: CCGCCTCCCGGGTTCACGCC). In all the cases, *GAPDH* was used as an internal normalization control (primers: GAPDH-Fwd: TGCACCACCAACTGCTTAGC; GAPDH-Rev: GGCATGGACTGTGGTCATGAG).

qPCR cycling parameters were as follows: 10 min at 95°C, 40 cycles of 15 sec at 95°C, followed by 60 sec at 60°C. Melting curve analysis was performed to confirm the identity of the amplified product. We employed the ΔΔCt method [225] to determine relative differences in transcript levels. L1 and Alu transcript levels were plotted as "Fold change in transcript level" with respect to the transcript level in H9-hESCs (=1). Standard deviations were calculated based on 4 data points per sample derived from duplicate measurements and a technical replicate for each sample.

RT-PCR reactions used GoTaq Green Master Mix (Promega) and primer pairs 1) L1 5'UTR forward (ACGGAATCTCGCTGATTGCTA) and L1 5'UTR reverse (AAGCAAGCCTGGGCAATG) [226], which amplify a 98-bp fragment of L1 5' UTR, 2) ORF1-fwd (AGGAAATACAGAGAACGCCACAA) and ORF1-rev (GCTGGATATGAAATTCTGGGTTGA), which amplify a 259-bp fragment of L1 ORF1, and 3) GACTBPAIR2-FOR (TTCCAGCCTTCCTTCCTG) and HACTBPAIR2-REV (AATGATCTTGATCTTCATTGTGC), which amplify a 207-bp fragment of actin beta. PCR conditions were 2 min at 95°C, 30 sec at 95°C, 1 min at 58°C, 30 sec at 72°C for 35 cycles, followed by 1 min at 72°C.

### Bioinformatic analysis

#### Occurrence of R159 polymorphisms in human L1 elements

L1Base 2 [13] was used for counting R159 polymorphism-containing human L1 elements. In brief, chromosome coordinates for human Full-Length, Intact LINE-1 elements (FLI-L1), human ORF2 Intact LINE-1 elements (ORF2-L1), and human Full-Length >4500nt LINE-1 elements (FLnI-L1) (Ens84.38) were obtained from L1Base 2. Corresponding sequences of L1 elements were retrieved using UCSC Table Browser [227]. Sequences of each subset were multiply aligned using MAFFT online [228] or as implemented in Geneious (Biomatters Ltd.; https://www.geneious.com; [229]). Occurrences within L1 ORF1 of a codon for R159 and its non-synonymous variants, including the most frequently observed codons for histidine (H), cysteine (C) or proline (P), were counted and their respective percentages calculated. Only a subset of the 13,671 L1 elements in the FLnI-L1 dataset were multiply aligned due to limitations of both the local and online versions of MAFFT. Also for the FLnI-L1 dataset, which included a greater number of evolutionarily older L1 sequences, a minority of aligned L1 sequences displayed structural rearrangements and/or higher sequence divergence in the R159 codon region resulting in unreliable prediction of sequence at the R159 codon position: these were excluded. In all, 6346 FLnI-L elements were included in the analysis.

#### RNA-Seq datasets

Publicly available RNA-Seq datasets were analyzed by TEtranscripts software package [150]. TDP-43-related datasets SRP057819 and GSE77702 have been previously described [147, 148]. Dataset SRP064478, submitted by the Bennett Lab at Virginia Commonwealth University, consists of RNA-Seq data for total stranded RNA with >50 million 2x150 bp sequencing reads from 15 postmortem cervical spinal cord sections (7 ALS and 8 healthy controls). GSE76220 includes 20-30 million mappable 1x50 bp reads from total stranded RNA isolated from laser capture microdissected motor neurons from post-mortem lumbar spinal cords [172]. GSE67196 consists of on average 83 million 1X100 bp reads per sample (91.5 million for cerebellum and 73.6 million for frontal cortex), as described by [52, 171].

The Library of Integrated Network-Based Cellular Signatures (LINCS)-NeuroLINCS dGAP dataset (accession number phs001231.v1.p1, SRP098831) includes RNA-Seq of iPSC-derived motor neurons from 4 C9ALS and 3 SMA patients (3 sequencing replicates each), and 3 unaffected healthy controls (2 or 3 replicates each). It has been reported that L1 activity in iPSCs can vary with cell passage, increasing during reprogramming but subsequently subsiding [224, 226, 230]. However, passage numbers of the NeuroLINCs cell lines fall within similar ranges, from 25 to 27 for ALS and healthy control and 21 to 30 for SMA samples.

#### Generating RPKM plots

To generate RPKM plots, raw data was aligned to the consensus sequences for a selected group of younger retrotransposons present in RepBase [152], including L1-Ta, L1PA2, L1-pre-Ta, AluYa5, AluYa8, AluYb8, AluYb9, AluY, AluSq, SVA_D, SVA_E, SVA_F, LTR5_Hs, and LTR7Y subfamilies. Alignments were made using Bowtie 2 [231] with end-to-end sensitive parameters: -D 15 -R 2 -N 0 -L 22 -i S, 1, 1.15. We adapted the RPKM formula to provide a normalized measure of the number of reads that align with each consensus sequence based on their size. Data were plotted using OriginPro (OriginLab Corp.) with standard deviation error bars for replicates.

MDS plots were generated in R script using the edgeR package [232].

#### Use of TEtranscripts

TEtranscripts is a software package that estimates both gene and TE transcript abundances in RNA-Seq data and conducts differential expression analysis on the resultant count tables [150]. Sequences were aligned to human genome assembly GRCh38 using STAR [233]. Alignment parameters were outFilterMultimapNmax100 and winAnchorMultimapNmax 200, which allow up to 100 alignments per read. TE annotation files were downloaded from http://labshare.cshl.edu/shares/mhammelllab/www-data/TEToolkit/ (including 1181 TE types). Following the generation of a count table for gene and TE transcripts, the differential expression analysis closely followed the DESeq2 package [234] for modeling the counts data with a negative binomial distribution and computing adjusted P-values. In addition to the standard transcript abundance normalization approach used by the DESeq2 package, TEtranscripts offers two additional options, reads per mapped million (RPM) and quantile normalization. All other procedures exactly followed the DESeq2 method. TEtranscripts runs the DESeq2 method with a default set of general parameters. When there were no (or very few) replicates, we used the blind method for variance estimation and fit-only for SharingMode. Otherwise, we used pooled or per-condition methods and maximum SharingMode, as suggested by the DESeq2 package.

#### Locus-specific mapping of TEs

The pipeline to map TEs to individual genomic loci used the alignment algorithm HISAT2 [235] to map sequence reads to the human genome. Reads that mapped to more than one genomic position were discarded. Counts per TE integrant (genomic loci) were generated using the multiBamCov tool from the BEDtools software [236]. Normalisation for sequencing depth was performed using voom [237], with total number of reads on genes as size factors. RepeatMasker 4.0.5 (Library 20140131), a newer version than RepeatMasker 4.0 used by Prudencio et al. [52], was used to generate a list of TE subfamilies. In the case of HERVs, we re-assembled fragmented internal and LTR sequences to generate full-length HERV integrants: this step avoids bias in counts due to the highly fragmented nature of the annotated HERVs. We removed from our analyses very small and abundant repeats (low complexity and simple repeats). Any TEs with a low number of reads across all samples or which overlapped exons were also omitted from our analyses. Differential expression was performed as implemented in the voom library of Bioconductor [238]. A TE locus was considered to be differentially expressed if its fold change was greater than 2 and FDR smaller than 0.05. The Benjamini-Hochberg procedure was used to compute the FDR. Hierarchical clustering of the heatmap was performed with Pearson correlation as distance and complete agglomeration method for both, rows and columns. Any raw data files will be provided upon request to the authors.

## Additional files

Additional file 1: **Figure S1.** Patterns of L1 ORF1p expression in various cell lines using multiple antibodies for detection. **Figure S2.** Frequency of polymorphisms and DNA methylation at the L1 ORF1p R159 residue. **Figure S3.** ORF1p does not contain a CRM1-dependent nuclear export signal. **Figure S4.** Cell culture toxicity assays. **Figure S5.** Overexpression or knockdown of TDP-43 protein does not alter L1 expression. **Figure S6.** Methylation analyses of the CpG island of the 5' UTR promoter of endogenous L1 elements show effects of altered levels of TDP-43. **Figure S7.** Expression levels of L1 (and Alu TEs determined by RT-qPCR. **Figure S8.** Representative gels showing expression of ORF1p in normal and ALS-associated tissues determined by Western blotting. **Figure S9.** TE locus-specific analyses of the GSE67196 RNA-Seq dataset [171]. (PDF 25088 kb)

Additional file 2: **Table S1.** Summary of significant DE TE subfamilies determined by TEtranscripts RNA-Seq datasets. (XLSX 326 kb)

Additional file 3: **Table S2.** Tissue samples used in this study. (PDF 65 kb)

Additional file 4: **Table S3.** Summary of significant individual DE TE loci in the GSE67196 RNA-Seq dataset. (XLSX 774 kb)

## Abbreviations

aa: Amino acids
ALS: Amyotrophic lateral sclerosis
bp: Base pair
CTRL: Control
DE: Differentially expressed
EGFP: Enhanced green fluorescent protein
fALS: Familial ALS
FISH: Fluorescent *in situ* hybridization
FDR: False discovery rate
FTD: Frontotemporal dementia
FTLD: Frontotemporal lobar degeneration
HEF: Human embryonic fibroblast
HERV: Human endogenous retrovirus
hESC: Human embryonic stem cell
IF: Immunofluorescence
iPSC: Induced pluripotent stem cell
kb: Kilobase
kD: Kilodalton
LINE-1/L1: Long interspersed element 1
LTR: Long terminal repeat
MDS: Multidimensional scaling
NES: Nuclear export signal
NLS: Nuclear localization signal
nt: Nucleotide
ORF: Open reading frame
padj: Adjusted P-value
PCA: Principal component analysis
PCR: Polymerase chain reaction
RNP: Ribonucleoprotein
RT-qPCR: Reverse transcription quantitative PCR
RNA-Seq: RNA sequencing
RT: Reverse transcriptase
sALS: Sporadic ALS
SG: Stress granule
SMA: Spinal muscular atrophy
SVA: SINE/VNTR/Alu
TE: Transposable element

## References

[1] McClintock B (1950). The origin and behavior of mutable loci in maize. Proc Natl Acad Sci U S A..

[2] Kazazian HH, Moran JV (2017). Mobile DNA in Health and Disease. N Engl J Med..

[3] Wildschutte JH, Williams ZH, Montesion M, Subramanian RP, Kidd JM, Coffin JM (2016). Discovery of unfixed endogenous retrovirus insertions in diverse human populations. Proc Natl Acad Sci U S A..

[4] Mager DL, Stoye JP (2015). Mammalian Endogenous Retroviruses.

[5] Richardson SR, Moran JV, Kopera HC, Doucet AJ, Moldovan JB, Garcia-Perez JL (2015). The Influence of LINE-1 and SINE Retrotransposons on Mammalian Genomes.

[6] Speek M (2001). Antisense promoter of human L1 retrotransposon drives transcription of adjacent cellular genes. Mol Cell Biol..

[7] Martin SL, Branciforte D, Keller D, Bain DL (2003). Trimeric structure for an essential protein in L1 retrotransposition. Proc Natl Acad Sci U S A..

[8] Martin SL, Cruceanu M, Branciforte D, Wai-Lun Li P, Kwok SC, Hodges RS, Williams MC (2005). LINE-1 retrotransposition requires the nucleic acid chaperone activity of the ORF1 protein. J Mol Biol..

[9] Khazina E, Truffault V, Buttner R, Schmidt S, Coles M, Weichenrieder O (2011). Trimeric structure and flexibility of the L1ORF1 protein in human L1 retrotransposition. Nat Struct Mol Biol..

[10] Brouha B, Schustak J, Badge RM, Lutz-Prigge S, Farley AH, Moran JV, Kazazian HH (2003). Hot L1s account for the bulk of retrotransposition in the human population. Proc Natl Acad Sci U S A..

[11] Mills RE, Bennett EA, Iskow RC, Devine SE (2007). Which transposable elements are active in the human genome?. Trends Genet..

[12] Beck CR, Collier P, Macfarlane C, Malig M, Kidd JM, Eichler EE, Badge RM, Moran JV (2010). LINE-1 retrotransposition activity in human genomes. Cell..

[13] Penzkofer T, Jager M, Figlerowicz M, Badge R, Mundlos S, Robinson PN, Zemojtel T (2017). L1Base 2: more retrotransposition-active LINE-1s, more mammalian genomes. Nucleic Acids Res..

[14] Sokolowski M, Chynces M (2017). deHaro D, Christian CM, Belancio VP. Truncated ORF1 proteins can suppress LINE-1 retrotransposition in trans. Nucleic Acids Res..

[15] Scott EC, Devine SE. The Role of Somatic L1 Retrotransposition in Human Cancers. Viruses. 2017;9(6). 10.3390/v9060131 PMC5490808 28561751

[16] Faulkner GJ, Garcia-Perez JL (2017). L1 Mosaicism in Mammals: Extent, Effects, and Evolution. Trends Genet..

[17] Suarez NA, Macia A, Muotri AR. LINE-1 retrotransposons in healthy and diseased human brain. Dev Neurobiol. 2017;78(5):434-55. 10.1002/dneu.22567 PMC5897138 29239145

[18] Perrat PN, DasGupta S, Wang J, Theurkauf W, Weng Z, Rosbash M, Waddell S (2013). Transposition-driven genomic heterogeneity in the Drosophila brain. Science..

[19] Yang F, Wang PJ. Multiple LINEs of retrotransposon silencing mechanisms in the mammalian germline. Semin Cell Dev Biol. 2016;59:118-25. 10.1016/j.semcdb.2016.03.001 PMC5011444 26957474

[20] Muotri AR, Chu VT, Marchetto MCN, Deng W, Moran JV, Gage FH (2005). Somatic mosaicism in neuronal precursor cells mediated by L1 retrotransposition. Nature..

[21] Coufal NG, Garcia-Perez JL, Peng GE, Yeo GW, Mu Y, Lovci MT, Morell M, O’Shea KS, Moran JV, Gage FH (2009). L1 retrotransposition in human neural progenitor cells. Nature..

[22] Blaudin de The FX, Rekaik H, Peze-Heidsieck E, Massiani-Beaudoin O, Joshi RL, Fuchs J, Prochiantz A. Engrailed homeoprotein blocks degeneration in adult dopaminergic neurons through LINE-1 repression. EMBO J. 2018;37(15):e97374. 10.15252/embj.201797374 PMC6068427 29941661

[23] Macia A, Widmann TJ, Heras SR, Ayllon V, Sanchez L, Benkaddour-Boumzaouad M, Munoz-Lopez M, Rubio A, Amador-Cubero S, Blanco-Jimenez E, Garcia-Castro J, Menendez P, Ng P, Muotri AR, Goodier JL, Garcia-Perez JL (2017). Engineered LINE-1 retrotransposition in nondividing human neurons. Genome Res..

[24] Thomas CA, Paquola AC, Muotri AR (2012). LINE-1 retrotransposition in the nervous system. Annu Rev Cell Dev Biol..

[25] Reilly MT, Faulkner GJ, Dubnau J, Ponomarev I, Gage FH (2013). The role of transposable elements in health and diseases of the central nervous system. J Neurosci..

[26] Erwin JA, Marchetto MC, Gage FH (2014). Mobile DNA elements in the generation of diversity and complexity in the brain. Nature Rev Neurosci..

[27] Richardson SR, Morell S, Faulkner GJ (2014). L1 retrotransposons and somatic mosaicism in the brain. Annu Rev Genet..

[28] Singer T, McConnell MJ, Marchetto MC, Coufal NG, Gage FH (2010). LINE-1 retrotransposons: mediators of somatic variation in neuronal genomes?. Trends Neurosci..

[29] Coufal NG, Garcia-Perez JL, Peng GE, Marchetto MCN, Muotri AR, Mu Y, Carson CT, Macia A, Moran JV, Gage FH (2011). Ataxia telangiectasia mutated (ATM) modulates long interspersed element-1 (L1) retrotransposition in human neural stem cells. Proc Natl Acad Sci U S A..

[30] Muotri AR, Marchetto MCN, Coufal NG, Oefner R, Yeo G, Nakashima K, Gage FH (2010). L1 retrotransposition in neurons is modulated by MeCP2. Nature..

[31] Jacob-Hirsch J, Eyal E, Knisbacher BA, Roth J, Cesarkas K, Dor C, Farage-Barhom S, Kunik V, Simon AJ, Gal M, Yalon M, Moshitch-Moshkovitz S, Tearle R, Constantini S, Levanon EY, Amariglio N, Rechavi G (2018). Whole-genome sequencing reveals principles of brain retrotransposition in neurodevelopmental disorders. Cell Res..

[32] Bundo M, Toyoshima M, Okada Y, Akamatsu W, Ueda J, Nemoto-Miyauchi T, Sunaga F, Toritsuka M, Ikawa D, Kakita A, Kato M, Kasai K, Kishimoto T, Nawa H, Okano H, Yoshikawa T, Kato T, Iwamoto K (2014). Increased L1 retrotransposition in the neuronal genome in schizophrenia. Neuron..

[33] Doyle GA, Crist RC, Karatas ET, Hammond MJ, Ewing AD, Ferraro TN, Hahn CG, Berrettini WH (2017). Analysis of LINE-1 Elements in DNA from Postmortem Brains of Individuals with Schizophrenia. Neuropsychopharmacology..

[34] Liu S, Du T, Liu Z, Shen Y, Xiu J, Xu Q (2016). Inverse changes in L1 retrotransposons between blood and brain in major depressive disorder. Sci Rep..

[35] Okudaira N, Ishizaka Y, Nishio H (2014). Retrotransposition of long interspersed element 1 induced by methamphetamine or cocaine. J Biol Chem..

[36] Okudaira N, Ishizaka Y, Nishio H, Sakagami H (2016). Morphine and Fentanyl Citrate Induce Retrotransposition of Long Interspersed Element-1. In Vivo..

[37] Doyle GA, Doucet-O'Hare TT, Hammond MJ, Crist RC, Ewing AD, Ferraro TN, Mash DC, Kazazian HH, Berrettini WH (2017). Reading LINEs within the cocaine addicted brain. Brain Behav..

[38] Tan H, Wu C, Jin L (2018). A Possible Role for Long Interspersed Nuclear Elements-1 (LINE-1) in Huntington's Disease Progression. Med Sci Monit..

[39] Bachiller S, Del-Pozo-Martin Y, Carrion AM (2017). L1 retrotransposition alters the hippocampal genomic landscape enabling memory formation. Brain Behav Immun..

[40] Bedrosian TA, Quayle C, Novaresi N, Gage FH (2018). Early life experience drives structural variation of neural genomes in mice. Science..

[41] Goodier JL (2014). Retrotransposition in tumors and brains. Mobile DNA..

[42] Goodier JL (2016). Restricting retrotransposons: a review. Mobile DNA..

[43] Brown RH, Al-Chalabi A (2017). Amyotrophic Lateral Sclerosis. N Engl J Med..

[44] Burrell JR, Halliday GM, Kril JJ, Ittner LM, Gotz J, Kiernan MC, Hodges JR (2016). The frontotemporal dementia-motor neuron disease continuum. Lancet..

[45] Steele AJ, Al-Chalabi A, Ferrante K, Cudkowicz ME, Brown RH, Garson JA (2005). Detection of serum reverse transcriptase activity in patients with ALS and unaffected blood relatives. Neurology..

[46] MacGowan DJ, Scelsa SN, Imperato TE, Liu KN, Baron P, Polsky B (2007). A controlled study of reverse transcriptase in serum and CSF of HIV-negative patients with ALS. Neurology..

[47] McCormick AL, Brown RH, Cudkowicz ME, Al-Chalabi A, Garson JA (2008). Quantification of reverse transcriptase in ALS and elimination of a novel retroviral candidate. Neurology..

[48] Alfahad T, Nath A (2013). Retroviruses and amyotrophic lateral sclerosis. Antiviral Res..

[49] Douville R, Liu J, Rothstein J, Nath A (2011). Identification of active loci of a human endogenous retrovirus in neurons of patients with amyotrophic lateral sclerosis. Ann Neurol..

[50] Hadlock K, Miller R, Jin X, Yu S, Reis J, Mass J, Gelinas D, McGrath M (2004). Elevated rates of antibody reactivity to HML-2/Herv-K but not other endogenous retroviruses in ALS. Amyotroph Lateral Scler Other Motor Neuron Disord..

[51] Li W, Lee MH, Henderson L, Tyagi R, Bachani M, Steiner J, Campanac E, Hoffman DA, von Geldern G, Johnson K, Maric D, Morris HD, Lentz M, Pak K, Mammen A, Ostrow L, Rothstein J, Nath A. Human endogenous retrovirus-K contributes to motor neuron disease. Sci Transl Med. 2015;7(307):307ra153-307ra153. 10.1126/scitranslmed.aac8201 PMC6344353 26424568

[52] Prudencio M, Gonzales PK, Cook CN, Gendron TF, Daughrity LM, Song Y, Ebbert MTW, van Blitterswijk M, Zhang YJ, Jansen-West K, Baker MC, DeTure M, Rademakers R, Boylan KB, Dickson DW, Petrucelli L, Link CD (2017). Repetitive element transcripts are elevated in the brain of C9orf72 ALS/FTLD patients. Hum Mol Genet..

[53] Li HF, Wu ZY (2016). Genotype-phenotype correlations of amyotrophic lateral sclerosis. Transl Neurodegener..

[54] Al-Chalabi A, Jones A, Troakes C, King A, Al-Sarraj S, van den Berg LH (2012). The genetics and neuropathology of amyotrophic lateral sclerosis. Acta Neuropathol..

[55] Baralle M, Buratti E, Baralle FE (2013). The role of TDP-43 in the pathogenesis of ALS and FTLD. Biochem Soc Trans..

[56] Nakashima-Yasuda H, Uryu K, Robinson J, Xie SX, Hurtig H, Duda JE, Arnold SE, Siderowf A, Grossman M, Leverenz JB, Woltjer R, Lopez OL, Hamilton R, Tsuang DW, Galasko D, Masliah E, Kaye J, Clark CM, Montine TJ, Lee VM, Trojanowski JQ (2007). Co-morbidity of TDP-43 proteinopathy in Lewy body related diseases. Acta Neuropathol..

[57] Amador-Ortiz C, Lin WL, Ahmed Z, Personett D, Davies P, Duara R, Graff-Radford NR, Hutton ML, Dickson DW (2007). TDP-43 immunoreactivity in hippocampal sclerosis and Alzheimer's disease. Ann Neurol..

[58] Schwab C, Arai T, Hasegawa M, Yu S, McGeer PL (2008). Colocalization of transactivation-responsive DNA-binding protein 43 and huntingtin in inclusions of Huntington disease. J Neuropathol Exp Neurol..

[59] Weihl CC, Temiz P, Miller SE, Watts G, Smith C, Forman M, Hanson PI, Kimonis V, Pestronk A (2008). TDP-43 accumulation in inclusion body myopathy muscle suggests a common pathogenic mechanism with frontotemporal dementia. J Neurol Neurosurg Psychiatry..

[60] Goodier JL, Zhang L, Vetter MR, Kazazian HH (2007). LINE-1 ORF1 protein localizes in stress granules with other RNA-binding proteins, including components of RNA interference RNA-induced silencing complex. Mol Cell Biol..

[61] Doucet AJ, Hulme AE, Sahinovic E, Kulpa DA, Moldovan JB, Kopera HC, Athanikar JN, Hasnaoui M, Bucheton A, Moran JV, Gilbert N (2010). Characterization of LINE-1 ribonucleoprotein particles. PLoS Genet..

[62] Cook PR, Jones CE, Furano AV (2015). Phosphorylation of ORF1p is required for L1 retrotransposition. Proc Natl Acad Sci U S A..

[63] MacLennan M, Garcia-Canadas M, Reichmann J, Khazina E, Wagner G, Playfoot CJ, Salvador-Palomeque C, Mann AR, Peressini P, Sanchez L, Dobie K, Read D, Hung CC, Eskeland R, Meehan RR, Weichenrieder O, Garcia-Perez JL, Adams IR. Mobilization of LINE-1 retrotransposons is restricted by Tex19.1 in mouse embryonic stem cells. Elife. 2017;6:e26152. 10.7554/eLife.26152 PMC5570191 28806172

[64] Martin SL, Branciforte D (1993). Synchronous expression of LINE-1 RNA and protein in mouse embryonal carcinoma cells. Mol Cell Biol..

[65] Horn AV, Klawitter S, Held U, Berger A, Vasudevan AAJ, Bock A, Hofmann H, Hanschmann K-MO, Trösemeier J-H, Flory E (2014). Human LINE-1 restriction by APOBEC3C is deaminase independent and mediated by an ORF1p interaction that affects LINE reverse transcriptase activity. Nucleic Acids Res..

[66] Hu S, Li J, Xu F, Mei S, Le Duff Y, Yin L, Pang X, Cen S, Jin Q, Liang C, Guo F (2015). SAMHD1 Inhibits LINE-1 Retrotransposition by Promoting Stress Granule Formation. PLoS Genet..

[67] Rodić N, Sharma R, Sharma R, Zampella J, Dai L, Taylor MS, Hruban RH, Iacobuzio-Donahue CA, Maitra A, Torbenson MS, Goggins M, Shih I-M, Duffield AS, Montgomery EA, Gabrielson E, Netto GJ, Lotan TL, De Marzo AM, Westra W, Binder ZA, Orr BA, Gallia GL, Eberhart CG, Boeke JD, Harris CR, Burns KH (2014). Long interspersed element-1 protein expression is a hallmark of many human cancers. The American Journal of Pathology..

[68] Leibold DM, Swergold GD, Singer MF, Thayer RE, Dombroski BA, Fanning TG (1990). Translation of LINE-1 DNA elements in vitro and in human cells. Proc Natl Acad Sci U S A..

[69] Josephson R, Ording CJ, Liu Y, Shin S, Lakshmipathy U, Toumadje A, Love B, Chesnut JD, Andrews PW, Rao MS, Auerbach JM (2007). Qualification of embryonal carcinoma 2102Ep as a reference for human embryonic stem cell research. Stem Cells..

[70] Sivan G, Kedersha N, Elroy-Stein O (2007). Ribosomal slowdown mediates translational arrest during cellular division. Mol Cell Biol..

[71] Guo H, Chitiprolu M, Gagnon D, Meng L, Perez-Iratxeta C, Lagace D, Gibbings D (2014). Autophagy supports genomic stability by degrading retrotransposon RNA. Nature Commun..

[72] Goodier JL, Mandal PK, Zhang L, Kazazian HH (2010). Discrete subcellular partitioning of human retrotransposon RNAs despite a common mechanism of genome insertion. Hum Mol Genet..

[73] Taylor MS, LaCava J, Mita P, Molloy K, Huang CR, Li D, Adney Emily M, Jiang H, Burns Kathleen H, Chait Brian T, Rout Michael P, Boeke Jef D, Dai L (2013). Affinity proteomics reveals human host factors implicated in discrete stages of LINE-1 retrotransposition. Cell..

[74] Alisch RS, Garcia-Perez JL, Muotri AR, Gage FH, Moran JV (2006). Unconventional translation of mammalian LINE-1 retrotransposons. Genes Dev..

[75] Ergun S, Buschmann C, Heukeshoven J, Dammann K, Schnieders F, Lauke H, Chalajour F, Kilic N, Stratling WH, Schumann GG (2004). Cell type-specific expression of LINE-1 open reading frames 1 and 2 in fetal and adult human tissues. J Biol Chem..

[76] Goodier JL, Ostertag EM, Engleka K, Seleme M, Kazazian HH (2004). A potential role for the nucleolus in L1 retrotransposition. Hum Mol Genet..

[77] Chen L, Dahlstrom JE, Chandra A, Board P, Rangasamy D (2012). Prognostic value of LINE-1 retrotransposon expression and its subcellular localization in breast cancer. Breast Cancer Res Treat..

[78] Sokolowski M, DeFreece CB, Servant G, Kines KJ, de Haro DL, Belancio VP (2014). Development of a monoclonal antibody specific to the endonuclease domain of the human LINE-1 ORF2 protein. Mobile DNA.

[79] De Luca C, Guadagni F, Sinibaldi-Vallebona P, Sentinelli S, Gallucci M, Hoffmann A, Schumann GG, Spadafora C, Sciamanna I (2016). Enhanced expression of LINE-1-encoded ORF2 protein in early stages of colon and prostate transformation. Oncotarget..

[80] Harrison AF, Shorter J (2017). RNA-binding proteins with prion-like domains in health and disease. Biochem J..

[81] Zambrano R, Conchillo-Sole O, Iglesias V, Illa R, Rousseau F, Schymkowitz J, Sabate R, Daura X, Ventura S (2015). PrionW: a server to identify proteins containing glutamine/asparagine rich prion-like domains and their amyloid cores. Nucleic Acids Res..

[82] Alberti S, Halfmann R, King O, Kapila A, Lindquist S (2009). A systematic survey identifies prions and illuminates sequence features of prionogenic proteins. Cell..

[83] Couthouis J, Hart MP, Shorter J, De Jesus-Hernandez M, Erion R, Oristano R, Liu AX, Ramos D, Jethava N, Hosangadi D, Epstein J, Chiang A, Diaz Z, Nakaya T, Ibrahim F, Kim HJ, Solski JA, Williams KL, Mojsilovic-Petrovic J, Ingre C, Boylan K, Graff-Radford NR, Dickson DW, Clay-Falcone D, Elman L, McCluskey L, Greene R, Kalb RG, Lee VM, Trojanowski JQ, Ludolph A, Robberecht W, Andersen PM, Nicholson GA, Blair IP, King OD, Bonini NM, Van Deerlin V, Rademakers R, Mourelatos Z, Gitler AD (2011). A yeast functional screen predicts new candidate ALS disease genes. Proc Natl Acad Sci U S A.

[84] Moran JV, Holmes SE, Naas TP, DeBerardinis RJ, Boeke JD, Kazazian HH (1996). High frequency retrotransposition in cultured mammalian cells. Cell..

[85] Khazina E, Weichenrieder O (2009). Non-LTR retrotransposons encode noncanonical RRM domains in their first open reading frame. Proc Natl Acad Sci U S A..

[86] Kimberland ML, Divoky V, Prchal J, Schwahn U, Berger W, Kazazian HH (1999). Full-length human L1 insertions retain the capacity for high frequency retrotransposition in cultured cells. Hum Mol Genet..

[87] Ostertag EM, Prak E, DeBerardinis R, Moran JV, Kazazian HH (2000). Determination of L1 retrotransposition kinetics in cultured cells. Nucleic Acids Res..

[88] Kulpa DA, Moran JV (2006). Cis-preferential LINE-1 reverse transcriptase activity in ribonucleoprotein particles. Nat Struct Mol Biol..

[89] Bertrand E, Chartrand P, Schaefer M, Shenoy SM, Singer RH, Long RM (1998). Localization of ASH1 mRNA particles in living yeast. Mol Cell..

[90] Liu Q, Dreyfuss G (1996). A novel nuclear structure containing the survival of motor neurons protein. EMBO J..

[91] Wojciechowska M, Krzyzosiak WJ (2011). Cellular toxicity of expanded RNA repeats: focus on RNA foci. Hum Mol Genet..

[92] DeJesus-Hernandez M, Mackenzie IR, Boeve BF, Boxer AL, Baker M, Rutherford NJ, Nicholson AM, Finch NA, Flynn H, Adamson J, Kouri N, Wojtas A, Sengdy P, Hsiung GY, Karydas A, Seeley WW, Josephs KA, Coppola G, Geschwind DH, Wszolek ZK, Feldman H, Knopman DS, Petersen RC, Miller BL, Dickson DW, Boylan KB, Graff-Radford NR, Rademakers R (2011). Expanded GGGGCC hexanucleotide repeat in noncoding region of C9ORF72 causes chromosome 9p-linked FTD and ALS. Neuron..

[93] Renton AE, Majounie E, Waite A, Simon-Sanchez J, Rollinson S, Gibbs JR, Schymick JC, Laaksovirta H, van Swieten JC, Myllykangas L, Kalimo H, Paetau A, Abramzon Y, Remes AM, Kaganovich A, Scholz SW, Duckworth J, Ding J, Harmer DW, Hernandez DG, Johnson JO, Mok K, Ryten M, Trabzuni D, Guerreiro RJ, Orrell RW, Neal J, Murray A, Pearson J, Jansen IE, Sondervan D, Seelaar H, Blake D, Young K, Halliwell N, Callister JB, Toulson G, Richardson A, Gerhard A, Snowden J, Mann D, Neary D, Nalls MA, Peuralinna T, Jansson L, Isoviita VM, Kaivorinne AL, Holtta-Vuori M, Ikonen E, Sulkava R, Benatar M, Wuu J, Chio A, Restagno G, Borghero G, Sabatelli M, Heckerman D, Rogaeva E, Zinman L, Rothstein JD, Sendtner M, Drepper C, Eichler EE, Alkan C, Abdullaev Z, Pack SD, Dutra A, Pak E, Hardy J, Singleton A, Williams NM, Heutink P, Pickering-Brown S, Morris HR, Tienari PJ, Traynor BJ (2011). A hexanucleotide repeat expansion in C9ORF72 is the cause of chromosome 9p21-linked ALS-FTD. Neuron.

[94] Hensman Moss DJ, Poulter M, Beck J, Hehir J, Polke JM, Campbell T, Adamson G, Mudanohwo E, McColgan P, Haworth A, Wild EJ, Sweeney MG, Houlden H, Mead S, Tabrizi SJ (2014). C9orf72 expansions are the most common genetic cause of Huntington disease phenocopies. Neurology..

[95] Donnelly CJ, Zhang PW, Pham JT, Haeusler AR, Mistry NA, Vidensky S, Daley EL, Poth EM, Hoover B, Fines DM, Maragakis N, Tienari PJ, Petrucelli L, Traynor BJ, Wang J, Rigo F, Bennett CF, Blackshaw S, Sattler R, Rothstein JD (2013). RNA toxicity from the ALS/FTD C9ORF72 expansion is mitigated by antisense intervention. Neuron..

[96] Rossi S, Serrano A, Gerbino V, Giorgi A, Di Francesco L, Nencini M, Bozzo F, Schinina ME, Bagni C, Cestra G, Carri MT, Achsel T, Cozzolino M (2015). Nuclear accumulation of mRNAs underlies G4C2-repeat-induced translational repression in a cellular model of C9orf72 ALS. J Cell Sci..

[97] Kubo S, Seleme MC, Soifer HS, Perez JL, Moran JV, Kazazian HH, Kasahara N (2006). L1 retrotransposition in nondividing and primary human somatic cells. Proc Natl Acad Sci U S A..

[98] Shi X, Seluanov A, Gorbunova V (2007). Cell divisions are required for L1 retrotransposition. Mol Cell Biol..

[99] Xie Y, Mates L, Ivics Z, Izsvak Z, Martin SL, An W (2013). Cell division promotes efficient retrotransposition in a stable L1 reporter cell line. Mobile DNA..

[100] Mita P, Wudzinska A, Sun X, Andrade J, Nayak S, Kahler DJ, Badri S, LaCava J, Ueberheide B, Yun CY, Fenyo D, Boeke JD. LINE-1 protein localization and functional dynamics during the cell cycle. Elife. 2018;7. 10.7554/eLife.30058 PMC5821460 29309036

[101] Mueller S, Schittenhelm M, Honecker F, Malenke E, Lauber K, Wesselborg S, Hartmann JT, Bokemeyer C, Mayer F (2006). Cell-cycle progression and response of germ cell tumors to cisplatin in vitro. Int J Oncol..

[102] Ikegami S, Taguchi T, Ohashi M, Oguro M, Nagano H, Mano Y (1978). Aphidicolin prevents mitotic cell division by interfering with the activity of DNA polymerase-alpha. Nature..

[103] Pratt WM, Ruddon RW, Ensminger WD, Maybaum J (1994). The anticancer drugs.

[104] Sokolowski M, de Haro D, Christian CM, Kines KJ, Belancio VP (2013). Characterization of L1 ORF1p self-interaction and cellular localization using a mammalian two-hybrid system. PLoS One.

[105] Naufer MN, Furano AV, Williams MC. Protein-nucleic acid interactions of LINE-1 ORF1p. Semin Cell Dev Biol. 2018;S1084-9521(17):30451-2. 10.1016/j.semcdb.2018.03.019 PMC6428221 29596909

[106] Nishitani H, Taraviras S, Lygerou Z, Nishimoto T (2001). The human licensing factor for DNA replication Cdt1 accumulates in G1 and is destabilized after initiation of S-phase. J Biol Chem..

[107] McGarry TJ, Kirschner MW (1998). Geminin, an inhibitor of DNA replication, is degraded during mitosis. Cell..

[108] Ayala YM, Zago P, D'Ambrogio A, Xu YF, Petrucelli L, Buratti E, Baralle FE (2008). Structural determinants of the cellular localization and shuttling of TDP-43. J Cell Sci..

[109] la Cour T, Gupta R, Rapacki K, Skriver K, Poulsen FM, Brunak S (2003). NESbase version 1.0: a database of nuclear export signals. Nucleic Acids Res..

[110] Yang J, Bardes ES, Moore JD, Brennan J, Powers MA, Kornbluth S (1998). Control of cyclin B1 localization through regulated binding of the nuclear export factor CRM1. Genes Dev..

[111] Engel K, Kotlyarov A, Gaestel M (1998). Leptomycin B-sensitive nuclear export of MAPKAP kinase 2 is regulated by phosphorylation. EMBO J..

[112] Kanwal C, Li H, Lim CS (2002). Model system to study classical nuclear export signals. AAPS PharmSci..

[113] la Cour T, Kiemer L, Molgaard A, Gupta R, Skriver K, Brunak S (2004). Analysis and prediction of leucine-rich nuclear export signals. Protein Eng Des Sel..

[114] Garcia-Perez JL, Morell M, Scheys JO, Kulpa DA, Morell S, Carter CC, Hammer GD, Collins KL, O’Shea KS, Menendez P, Moran JV (2010). Epigenetic silencing of engineered L1 retrotransposition events in human embryonic carcinoma cells. Nature..

[115] Rosen DR, Siddique T, Patterson D, Figlewicz DA, Sapp P, Hentati A, Donaldson D, Goto J, O'Regan JP, Deng HX (1993). Mutations in Cu/Zn superoxide dismutase gene are associated with familial amyotrophic lateral sclerosis. Nature..

[116] Majounie E, Renton AE, Mok K, Dopper EG, Waite A, Rollinson S, Chio A, Restagno G, Nicolaou N, Simon-Sanchez J, van Swieten JC, Abramzon Y, Johnson JO, Sendtner M, Pamphlett R, Orrell RW, Mead S, Sidle KC, Houlden H, Rohrer JD, Morrison KE, Pall H, Talbot K, Ansorge O, Hernandez DG, Arepalli S, Sabatelli M, Mora G, Corbo M, Giannini F, Calvo A, Englund E, Borghero G, Floris GL, Remes AM, Laaksovirta H, McCluskey L, Trojanowski JQ, Van Deerlin VM, Schellenberg GD, Nalls MA, Drory VE, Lu CS, Yeh TH, Ishiura H, Takahashi Y, Tsuji S, Le Ber I, Brice A, Drepper C, Williams N, Kirby J, Shaw P, Hardy J, Tienari PJ, Heutink P, Morris HR, Pickering-Brown S, Traynor BJ (2012). Frequency of the C9orf72 hexanucleotide repeat expansion in patients with amyotrophic lateral sclerosis and frontotemporal dementia: a cross-sectional study. Lancet Neurol.

[117] Boeynaems S, Bogaert E, Van Damme P, Van Den Bosch L (2016). Inside out: the role of nucleocytoplasmic transport in ALS and FTLD. Acta Neuropathol..

[118] Ederle H, Dormann D (2017). TDP-43 and FUS en route from the nucleus to the cytoplasm. FEBS Lett..

[119] Colombrita C, Zennaro E, Fallini C, Weber M, Sommacal A, Buratti E, Silani V, Ratti A (2009). TDP-43 is recruited to stress granules in conditions of oxidative insult. J Neurochem..

[120] Liu-Yesucevitz L, Bilgutay A, Zhang YJ, Vanderweyde T, Citro A, Mehta T, Zaarur N, McKee A, Bowser R, Sherman M, Petrucelli L, Wolozin B (2010). Tar DNA binding protein-43 (TDP-43) associates with stress granules: analysis of cultured cells and pathological brain tissue. PLoS One..

[121] Dewey CM, Cenik B, Sephton CF, Dries DR, Mayer P, Good SK, Johnson BA, Herz J, Yu G (2011). TDP-43 is directed to stress granules by sorbitol, a novel physiological osmotic and oxidative stressor. Mol Cell Biol..

[122] Buratti E, Brindisi A, Giombi M, Tisminetzky S, Ayala YM, Baralle FE (2005). TDP-43 binds heterogeneous nuclear ribonucleoprotein A/B through its C-terminal tail: an important region for the inhibition of cystic fibrosis transmembrane conductance regulator exon 9 splicing. J Biol Chem..

[123] Kim HJ, Kim NC, Wang YD, Scarborough EA, Moore J, Diaz Z, MacLea KS, Freibaum B, Li S, Molliex A, Kanagaraj AP, Carter R, Boylan KB, Wojtas AM, Rademakers R, Pinkus JL, Greenberg SA, Trojanowski JQ, Traynor BJ, Smith BN, Topp S, Gkazi AS, Miller J, Shaw CE, Kottlors M, Kirschner J, Pestronk A, Li YR, Ford AF, Gitler AD, Benatar M, King OD, Kimonis VE, Ross ED, Weihl CC, Shorter J, Taylor JP (2013). Mutations in prion-like domains in hnRNPA2B1 and hnRNPA1 cause multisystem proteinopathy and ALS. Nature.

[124] Goodier JL, Cheung LE, Kazazian HH (2013). Mapping the LINE1 ORF1 protein interactome reveals associated inhibitors of human retrotransposition. Nucleic Acids Res..

[125] Mackenzie IR, Nicholson AM, Sarkar M, Messing J, Purice MD, Pottier C, Annu K, Baker M, Perkerson RB, Kurti A, Matchett BJ, Mittag T, Temirov J, Hsiung GR, Krieger C, Murray ME, Kato M, Fryer JD, Petrucelli L, Zinman L, Weintraub S, Mesulam M, Keith J, Zivkovic SA, Hirsch-Reinshagen V, Roos RP, Zuchner S, Graff-Radford NR, Petersen RC, Caselli RJ, Wszolek ZK, Finger E, Lippa C, Lacomis D, Stewart H, Dickson DW, Kim HJ, Rogaeva E, Bigio E, Boylan KB, Taylor JP, Rademakers R (2017). TIA1 Mutations in Amyotrophic Lateral Sclerosis and Frontotemporal Dementia Promote Phase Separation and Alter Stress Granule Dynamics. Neuron.

[126] Ash PE, Bieniek KF, Gendron TF, Caulfield T, Lin WL, Dejesus-Hernandez M, van Blitterswijk MM, Jansen-West K, Paul JW, Rademakers R, Boylan KB, Dickson DW, Petrucelli L (2013). Unconventional translation of C9ORF72 GGGGCC expansion generates insoluble polypeptides specific to c9FTD/ALS. Neuron..

[127] Mori K, Weng SM, Arzberger T, May S, Rentzsch K, Kremmer E, Schmid B, Kretzschmar HA, Cruts M, Van Broeckhoven C, Haass C, Edbauer D (2013). The C9orf72 GGGGCC repeat is translated into aggregating dipeptide-repeat proteins in FTLD/ALS. Science..

[128] Yamakawa M, Ito D, Honda T, Kubo K, Noda M, Nakajima K, Suzuki N (2015). Characterization of the dipeptide repeat protein in the molecular pathogenesis of c9FTD/ALS. Hum Mol Genet..

[129] Mizielinska S, Gronke S, Niccoli T, Ridler CE, Clayton EL, Devoy A, Moens T, Norona FE, Woollacott IOC, Pietrzyk J, Cleverley K, Nicoll AJ, Pickering-Brown S, Dols J, Cabecinha M, Hendrich O, Fratta P, Fisher EMC, Partridge L, Isaacs AM (2014). C9orf72 repeat expansions cause neurodegeneration in Drosophila through arginine-rich proteins. Science..

[130] Ji AL, Zhang X, Chen WW, Huang WJ (2017). Genetics insight into the amyotrophic lateral sclerosis/frontotemporal dementia spectrum. J Med Genet..

[131] Zhang YJ, Gendron TF, Grima JC, Sasaguri H, Jansen-West K, Xu YF, Katzman RB, Gass J, Murray ME, Shinohara M, Lin WL, Garrett A, Stankowski JN, Daughrity L, Tong J, Perkerson EA, Yue M, Chew J, Castanedes-Casey M, Kurti A, Wang ZS, Liesinger AM, Baker JD, Jiang J, Lagier-Tourenne C, Edbauer D, Cleveland DW, Rademakers R, Boylan KB, Bu G, Link CD, Dickey CA, Rothstein JD, Dickson DW, Fryer JD, Petrucelli L (2016). C9ORF72 poly(GA) aggregates sequester and impair HR23 and nucleocytoplasmic transport proteins. Nat Neurosci.

[132] Sendtner M (2011). TDP-43: multiple targets, multiple disease mechanisms?. Nat Neurosci..

[133] Lagier-Tourenne C, Polymenidou M, Cleveland DW (2010). TDP-43 and FUS/TLS: emerging roles in RNA processing and neurodegeneration. Hum Mol Genet..

[134] Ratti A, Buratti E (2016). Physiological functions and pathobiology of TDP-43 and FUS/TLS proteins. J Neurochem..

[135] Estes PS, Boehringer A, Zwick R, Tang JE, Grigsby B, Zarnescu DC (2011). Wild-type and A315T mutant TDP-43 exert differential neurotoxicity in a Drosophila model of ALS. Hum Mol Genet..

[136] Xu Z, Yang C (2014). TDP-43-The key to understanding amyotrophic lateral sclerosis. Rare Dis..

[137] Li W, Jin Y, Prazak L, Hammell M, Dubnau J (2012). Transposable elements in TDP-43-mediated neurodegenerative disorders. PLoS One..

[138] Manghera M, Ferguson-Parry J, Douville RN (2016). TDP-43 regulates endogenous retrovirus-K viral protein accumulation. Neurobiol Dis..

[139] Krug L, Chatterjee N, Borges-Monroy R, Hearn S, Liao WW, Morrill K, Prazak L, Rozhkov N, Theodorou D, Hammell M, Dubnau J (2017). Retrotransposon activation contributes to neurodegeneration in a Drosophila TDP-43 model of ALS. PLoS Genet..

[140] Moldovan JB. Identification of cellular host factors that associate with LINE-1 ORF1p and the effect of the Zinc Finger Antiviral Protein ZAP on LINE-1 retrotransposition [Ph.D. Thesis]: University of Michigan; 2015.

[141] Taylor MS, Altukhov I, Molloy KR, Mita P, Jiang H, Adney EM, Wudzinska A, Badri S, Ischenko D, Eng G, Burns KH, Fenyo D, Chait BT, Alexeev D, Rout MP, Boeke JD, LaCava J. Dissection of affinity captured LINE-1 macromolecular complexes. Elife. 2018;77:e30094. 10.7554/eLife.30094 PMC5821459 29309035

[142] Gao J, Wang L, Huntley ML, Perry G, Wang X. Pathomechanisms of TDP-43 in neurodegeneration. J Neurochem. 2018; 10.1111/jnc.14327. 10.1111/jnc.14327 PMC6110993 29486049

[143] Vogt MA, Ehsaei Z, Knuckles P, Higginbottom A, Helmbrecht MS, Kunath T, Eggan K, Williams LA, Shaw PJ, Wurst W, Floss T, Huber AB, Taylor V (2018). TDP-43 induces p53-mediated cell death of cortical progenitors and immature neurons. Sci Rep..

[144] Dewannieux M, Dupressoir A, Harper F, Pierron G, Heidmann T (2004). Identification of autonomous IAP LTR retrotransposons mobile in mammalian cells. Nat Genet..

[145] Crowther P, Doherty J, Linsenmeyer M, Williamson M, Woodcock D (1991). Revised genomic consensus for the hypermethylated CpG island region of the human L1 transposon and integration sites of full length L1 elements from recombinant clones made using methylation-tolerant host strains. Nucleic Acids Res..

[146] Munoz-Lopez M, Garcia-Canadas M, Macia A, Morell S, Garcia-Perez JL (2012). Analysis of LINE-1 expression in human pluripotent cells. Methods Mol Biol..

[147] Ling JP, Pletnikova O, Troncoso JC, Wong PC (2015). TDP-43 repression of nonconserved cryptic exons is compromised in ALS-FTD. Science..

[148] Kapeli K, Pratt GA, Vu AQ, Hutt KR, Martinez FJ, Sundararaman B, Batra R, Freese P, Lambert NJ, Huelga SC, Chun SJ, Liang TY, Chang J, Donohue JP, Shiue L, Zhang J, Zhu H, Cambi F, Kasarskis E, Hoon S, Ares M, Burge CB, Ravits J, Rigo F, Yeo GW (2016). Distinct and shared functions of ALS-associated proteins TDP-43, FUS and TAF15 revealed by multisystem analyses. Nat Commun..

[149] Svetoni F, Frisone P, Paronetto MP (2016). Role of FET proteins in neurodegenerative disorders. RNA Biol..

[150] Jin Y, Tam OH, Paniagua E, Hammell M (2015). TEtranscripts: a package for including transposable elements in differential expression analysis of RNA-seq datasets. Bioinformatics..

[151] Jurka J, Kapitonov VV, Pavlicek A, Klonowski P, Kohany O, Walichiewicz J (2005). Repbase Update, a database of eukaryotic repetitive elements. Cytogenet Genome Res..

[152] Bao W, Kojima KK, Kohany O (2015). Repbase Update, a database of repetitive elements in eukaryotic genomes. Mobile DNA..

[153] Pesiridis GS, Lee VM, Trojanowski JQ (2009). Mutations in TDP-43 link glycine-rich domain functions to amyotrophic lateral sclerosis. Hum Mol Genet..

[154] Winton MJ, Van Deerlin VM, Kwong LK, Yuan W, Wood EM, Yu CE, Schellenberg GD, Rademakers R, Caselli R, Karydas A, Trojanowski JQ, Miller BL, Lee VM (2008). A90V TDP-43 variant results in the aberrant localization of TDP-43 in vitro. FEBS Lett..

[155] Kovacs GG, Murrell JR, Horvath S, Haraszti L, Majtenyi K, Molnar MJ, Budka H, Ghetti B, Spina S (2009). TARDBP variation associated with frontotemporal dementia, supranuclear gaze palsy, and chorea. Mov Disord..

[156] Hooper LV, Stappenbeck TS, Hong CV, Gordon JI (2003). Angiogenins: a new class of microbicidal proteins involved in innate immunity. Nat Immunol..

[157] Kieran D, Sebastia J, Greenway MJ, King MA, Connaughton D, Concannon CG, Fenner B, Hardiman O, Prehn JH (2008). Control of motoneuron survival by angiogenin. J Neurosci..

[158] Li S, Chen Y, Sun D, Bai R, Gao X, Yang Y, Sheng J, Xu Z (2017). Angiogenin Prevents Progranulin A9D Mutation-Induced Neuronal-Like Cell Apoptosis Through Cleaving tRNAs into tiRNAs. Mol Neurobiol..

[159] Bradshaw WJ, Rehman S, Pham TT, Thiyagarajan N, Lee RL, Subramanian V, Acharya KR (2017). Structural insights into human angiogenin variants implicated in Parkinson's disease and Amyotrophic Lateral Sclerosis. Sci Rep..

[160] Gellera C, Colombrita C, Ticozzi N, Castellotti B, Bragato C, Ratti A, Taroni F, Silani V (2008). Identification of new ANG gene mutations in a large cohort of Italian patients with amyotrophic lateral sclerosis. Neurogenetics..

[161] van Es MA, Schelhaas HJ, van Vught PW, Ticozzi N, Andersen PM, Groen EJ, Schulte C, Blauw HM, Koppers M, Diekstra FP, Fumoto K, AL LC, Keagle P, Bloem BR, Scheffer H, van Nuenen BF, van Blitterswijk M, van Rheenen W, Wills AM, Lowe PP, Hu GF, Yu W, Kishikawa H, Wu D, Folkerth RD, Mariani C, Goldwurm S, Pezzoli G, Van Damme P, Lemmens R, Dahlberg C, Birve A, Fernandez-Santiago R, Waibel S, Klein C, Weber M, van der Kooi AJ, de Visser M, Verbaan D, van Hilten JJ, Heutink P, Hennekam EA, Cuppen E, Berg D, Brown RH, Silani V, Gasser T, Ludolph AC, Robberecht W, Ophoff RA, Veldink JH, Pasterkamp RJ, de Bakker PI, Landers JE, van de Warrenburg BP, van den Berg LH (2011). Angiogenin variants in Parkinson disease and amyotrophic lateral sclerosis. Ann Neurol.

[162] Bennett EA, Keller H, Mills RE, Schmidt S, Moran JV, Weichenrieder O, Devine SE (2008). Active Alu retrotransposons in the human genome. Genome Res..

[163] Kryatova MS, Steranka JP, Burns KH, Payer LM (2017). Insertion and deletion polymorphisms of the ancient AluS family in the human genome. Mobile DNA..

[164] Batzer MA, Deininger PL (2002). Alu repeats and human genomic diversity. Nat Rev Genet..

[165] Hancks DC, Kazazian HH. Roles for retrotransposon insertions in human disease. Mobile DNA. 2016;7(1):9. 10.1186/s13100-016-0065-9 PMC4859970 27158268

[166] Seleme MC, Vetter MR, Cordaux R, Bastone L, Batzer MA, Kazazian HH (2006). Extensive individual variation in L1 retrotransposition capability contributes to human genetic diversity. Proc Natl Acad Sci U S A..

[167] Thomson JA, Itskovitz-Eldor J, Shapiro SS, Waknitz MA, Swiergiel JJ, Marshall VS, Jones JM (1998). Embryonic stem cell lines derived from human blastocysts. Science..

[168] Garcia-Perez JL, Marchetto MC, Muotri AR, Coufal NG, Gage FH, O'Shea KS, Moran JV (2007). LINE-1 retrotransposition in human embryonic stem cells. Hum Mol Genet..

[169] Moszczynska A, Flack A, Qiu P, Muotri AR, Killinger BA (2015). Neurotoxic Methamphetamine Doses Increase LINE-1 Expression in the Neurogenic Zones of the Adult Rat Brain. Sci Rep..

[170] Sur D, Kustwar RK, Budania S, Mahadevan A, Hancks DC, Yadav V, Shankar SK, Mandal PK (2017). Detection of the LINE-1 retrotransposon RNA-binding protein ORF1p in different anatomical regions of the human brain. Mobile DNA..

[171] Prudencio M, Belzil VV, Batra R, Ross CA, Gendron TF, Pregent LJ, Murray ME, Overstreet KK, Piazza-Johnston AE, Desaro P, Bieniek KF, DeTure M, Lee WC, Biendarra SM, Davis MD, Baker MC, Perkerson RB, van Blitterswijk M, Stetler CT, Rademakers R, Link CD, Dickson DW, Boylan KB, Li H, Petrucelli L (2015). Distinct brain transcriptome profiles in C9orf72-associated and sporadic ALS. Nat Neurosci..

[172] Batra R, Hutt K, Vu A, Rabin SJ, Baughn MW, Libby RT, Hoon S, Ravits J, Yeo GW. Gene Expression Signatures of Sporadic ALS Motor Neuron Populations. bioRxiv. 2016. 10.1101/038448.

[173] Larsen PA, Hunnicutt KE, Larsen RJ, Yoder AD, Saunders AM (2018). Warning SINEs: Alu elements, evolution of the human brain, and the spectrum of neurological disease. Chromosome Res..

[174] Deininger P, Morales ME, White TB, Baddoo M, Hedges DJ, Servant G, Srivastav S, Smither ME, Concha M, DeHaro DL, Flemington EK, Belancio VP (2017). A comprehensive approach to expression of L1 loci. Nucleic Acids Res..

[175] Philippe C, Vargas-Landin DB, Doucet AJ, van Essen D, Vera-Otarola J, Kuciak M, Corbin A, Nigumann P, Cristofari G. Activation of individual L1 retrotransposon instances is restricted to cell-type dependent permissive loci. Elife. 2016;5:e13926. 10.7554/eLife.13926 PMC4866827 27016617

[176] Branciforte D, Martin SL (1994). Developmental and cell type specificity of LINE-1 expression in mouse testis: implications for transposition. Mol Cell Biol..

[177] Horn AV, Celic I, Dong C, Martirosyan I, Han JS (2017). A conserved role for the ESCRT membrane budding complex in LINE retrotransposition. PLoS Genet..

[178] Tiruchinapalli DM, Oleynikov Y, Kelic S, Shenoy SM, Hartley A, Stanton PK, Singer RH, Bassell GJ (2003). Activity-dependent trafficking and dynamic localization of zipcode binding protein 1 and beta-actin mRNA in dendrites and spines of hippocampal neurons. J Neurosci..

[179] Donnelly CJ, Willis DE, Xu M, Tep C, Jiang C, Yoo S, Schanen NC, Kirn-Safran CB, van Minnen J, English A, Yoon SO, Bassell GJ, Twiss JL (2011). Limited availability of ZBP1 restricts axonal mRNA localization and nerve regeneration capacity. EMBO J..

[180] Fallini C, Rouanet JP, Donlin-Asp PG, Guo P, Zhang H, Singer RH, Rossoll W, Bassell GJ (2014). Dynamics of survival of motor neuron (SMN) protein interaction with the mRNA-binding protein IMP1 facilitates its trafficking into motor neuron axons. Dev Neurobiol..

[181] Jonson L, Vikesaa J, Krogh A, Nielsen LK, Hansen T, Borup R, Johnsen AH, Christiansen J, Nielsen FC (2007). Molecular composition of IMP1 ribonucleoprotein granules. Mol Cell Proteomics..

[182] Harris CR, Normart R, Yang Q, Stevenson E, Haffty BG, Ganesan S, Cordon-Cardo C, Levine AJ, Tang LH (2010). Association of nuclear localization of a Long Interspersed Nuclear Element-1 Protein in breast tumors with poor prognostic outcomes. Genes Cancer..

[183] Lucchinetti E, Feng J, Silva R, Tolstonog GV, Schaub MC, Schumann GG, Zaugg M (2006). Inhibition of LINE-1 expression in the heart decreases ischemic damage by activation of Akt/PKB signaling. Physiol Genomics..

[184] Kirilyuk A, Tolstonog GV, Damert A, Held U, Hahn S, Lower R, Buschmann C, Horn AV, Traub P, Schumann GG (2008). Functional endogenous LINE-1 retrotransposons are expressed and mobilized in rat chloroleukemia cells. Nucleic Acids Res..

[185] Soper SF, van der Heijden GW, Hardiman TC, Goodheart M, Martin SL, de Boer P, Bortvin A (2008). Mouse maelstrom, a component of nuage, is essential for spermatogenesis and transposon repression in meiosis. Dev Cell..

[186] Malki S, van der Heijden GW, O’Donnell Kathryn A, Martin Sandra L, Bortvin A (2014). A role for retrotransposon LINE-1 in fetal oocyte attrition in mice. Dev Cell..

[187] Luo YB, Zhang L, Lin ZL, Ma JY, Jia J, Namgoong S, Sun QY (2016). Distinct subcellular localization and potential role of LINE1-ORF1P in meiotic oocytes. Histochem Cell Biol..

[188] Idica A, Sevrioukov EA, Zisoulis DG, Hamdorf M, Daugaard I, Kadandale P, Pedersen IM (2017). MicroRNA miR-128 represses LINE-1 (L1) retrotransposition by down-regulating the nuclear import factor TNPO1. J Biol Chem..

[189] Mandal PK, Ewing AD, Hancks DC, Kazazian HH (2013). Enrichment of processed pseudogene transcripts in L1-ribonucleoprotein particles. Hum Mol Genet..

[190] Moldovan JB, Moran JV (2015). The Zinc-Finger Antiviral Protein ZAP Inhibits LINE and Alu Retrotransposition. PLoS Genet..

[191] Gasior SL, Wakeman TP, Xu B, Deininger PL (2006). The human LINE-1 retrotransposon creates DNA double-strand breaks. J Mol Biol..

[192] Belgnaoui SM, Gosden RG, Semmes OJ, Haoudi A (2006). Human LINE-1 retrotransposon induces DNA damage and apoptosis in cancer cells. Cancer Cell Int..

[193] Wallace NA, Belancio VP, Deininger PL (2008). L1 mobile element expression causes multiple types of toxicity. Gene..

[194] Yu Q, Carbone CJ, Katlinskaya YV, Zheng H, Zheng K, Luo M, Wang PJ, Greenberg RA, Fuchs SY (2015). Type I Interferon controls propagation of Long Interspersed Element-1. J Biol Chem..

[195] Thomas CA, Tejwani L, Trujillo CA, Negraes PD, Herai RH, Mesci P, Macia A, Crow YJ, Muotri AR (2017). Modeling of TREX1-Dependent Autoimmune Disease using Human Stem Cells Highlights L1 Accumulation as a Source of Neuroinflammation. Cell Stem Cell.

[196] Ou SH, Wu F, Harrich D, Garcia-Martinez LF, Gaynor RB (1995). Cloning and characterization of a novel cellular protein, TDP-43, that binds to human immunodeficiency virus type 1 TAR DNA sequence motifs. J Virol..

[197] Nehls J, Koppensteiner H, Brack-Werner R, Floss T, Schindler M (2014). HIV-1 replication in human immune cells is independent of TAR DNA binding protein 43 (TDP-43) expression. PLoS One..

[198] Douville RN, Nath A (2017). Human Endogenous Retrovirus-K and TDP-43 Expression Bridges ALS and HIV Neuropathology. Front Microbiol..

[199] Polymenidou M, Lagier-Tourenne C, Hutt KR, Huelga SC, Moran J, Liang TY, Ling SC, Sun E, Wancewicz E, Mazur C, Kordasiewicz H, Sedaghat Y, Donohue JP, Shiue L, Bennett CF, Yeo GW, Cleveland DW (2011). Long pre-mRNA depletion and RNA missplicing contribute to neuronal vulnerability from loss of TDP-43. Nat Neurosci..

[200] Shan X, Chiang PM, Price DL, Wong PC (2010). Altered distributions of Gemini of coiled bodies and mitochondria in motor neurons of TDP-43 transgenic mice. Proc Natl Acad Sci U S A..

[201] Dhellin O, Maestre J, Heidmann T (1997). Functional differences between the human LINE retrotransposon and retroviral reverse transcriptases for invivo mRNA reverse transcription. EMBO J.

[202] Garcia Perez JL, Alarcon-Riquelme ME (2017). The TREX1 Dinosaur Bites the Brain through the LINE. Cell Stem Cell..

[203] Sciamanna I, Gualtieri A, Cossetti C, Osimo EF, Ferracin M, Macchia G, Arico E, Prosseda G, Vitullo P, Misteli T, Spadafora C (2013). A tumor-promoting mechanism mediated by retrotransposon-encoded reverse transcriptase is active in human transformed cell lines. Oncotarget..

[204] Mayer J, Harz C, Sanchez L, Pereira GC, Maldener E, Heras SR, Ostrow LW, Ravits J, Batra R, Meese E, Garcia-Perez JL, Goodier JL (2018). Transcriptional profiling of HERV-K(HML-2) in amyotrophic lateral sclerosis and potential implications for expression of HML-2 proteins. Mol Neurodegener..

[205] Chestnut BA, Chang Q, Price A, Lesuisse C, Wong M, Martin LJ (2011). Epigenetic regulation of motor neuron cell death through DNA methylation. J Neurosci..

[206] Martin LJ, Wong M (2013). Aberrant regulation of DNA methylation in amyotrophic lateral sclerosis: a new target of disease mechanisms. Neurotherapeutics..

[207] Coppede F, Stoccoro A, Mosca L, Gallo R, Tarlarini C, Lunetta C, Marocchi A, Migliore L, Penco S (2018). Increase in DNA methylation in patients with amyotrophic lateral sclerosis carriers of not fully penetrant SOD1 mutations. Amyotroph Lateral Scler Frontotemporal Degener..

[208] Hamzeiy H, Savas D, Tunca C, Sen NE, Gundogdu Eken A, Sahbaz I, Calini D, Tiloca C, Ticozzi N, Ratti A, Silani V, Basak AN (2018). Elevated Global DNA Methylation Is Not Exclusive to Amyotrophic Lateral Sclerosis and Is Also Observed in Spinocerebellar Ataxia Types 1 and 2. Neurodegener Dis..

[209] de Koning AP, Gu W, Castoe TA, Batzer MA, Pollock DD (2011). Repetitive elements may comprise over two-thirds of the human genome. PLoS Genet..

[210] Coady TH, Manley JL (2015). ALS mutations in TLS/FUS disrupt target gene expression. Genes Dev..

[211] Maruyama H, Morino H, Ito H, Izumi Y, Kato H, Watanabe Y, Kinoshita Y, Kamada M, Nodera H, Suzuki H, Komure O, Matsuura S, Kobatake K, Morimoto N, Abe K, Suzuki N, Aoki M, Kawata A, Hirai T, Kato T, Ogasawara K, Hirano A, Takumi T, Kusaka H, Hagiwara K, Kaji R, Kawakami H (2010). Mutations of optineurin in amyotrophic lateral sclerosis. Nature..

[212] Mao YS, Sunwoo H, Zhang B, Spector DL (2011). Direct visualization of the co-transcriptional assembly of a nuclear body by noncoding RNAs. Nat Cell Biol..

[213] Liu-Yesucevitz L, Lin AY, Ebata A, Boon JY, Reid W, Xu YF, Kobrin K, Murphy GJ, Petrucelli L, Wolozin B (2014). ALS-linked mutations enlarge TDP-43-enriched neuronal RNA granules in the dendritic arbor. J Neurosci..

[214] Zhang T, Baldie G, Periz G, Wang J (2014). RNA-processing protein TDP-43 regulates FOXO-dependent protein quality control in stress response. PLoS Genet..

[215] Farrawell NE, Lambert-Smith IA, Warraich ST, Blair IP, Saunders DN, Hatters DM, Yerbury JJ (2015). Distinct partitioning of ALS associated TDP-43, FUS and SOD1 mutants into cellular inclusions. Sci Rep..

[216] Osaka M, Ito D, Yagi T, Nihei Y, Suzuki N (2015). Evidence of a link between ubiquilin 2 and optineurin in amyotrophic lateral sclerosis. Hum Mol Genet..

[217] Soifer H, Higo C, Kazazian HH, Moran JV, Mitani K, Kasahara N (2001). Stable integration of transgenes delivered by a retrotransposon-adenovirus hybrid vector. Hum Gene Ther..

[218] Palmer DJ, Ng P (2008). Methods for the production of helper-dependent adenoviral vectors. Methods Mol Biol..

[219] Hulme AE, Bogerd HP, Cullen BR, Moran JV (2007). Selective inhibition of Alu retrotransposition by APOBEC3G. Gene..

[220] Stoecklin G, Mayo T, Anderson P (2006). ARE-mRNA degradation requires the 5'-3' decay pathway. EMBO Rep..

[221] Goodier JL, Cheung LE, Kazazian HH (2012). MOV10 RNA helicase is a potent inhibitor of retrotransposition in cells. PLoS Genet..

[222] Goodier JL, Pereira GC, Cheung LE, Rose RJ, Kazazian HH (2015). The Broad-Spectrum Antiviral Protein ZAP Restricts Human Retrotransposition. PLoS Genet..

[223] Kumaki Y, Oda M, Okano M (2008). QUMA: quantification tool for methylation analysis. Nucleic Acids Res.

[224] Klawitter S, Fuchs NV, Upton KR, Muñoz-Lopez M, Shukla R, Wang J, Garcia-Cañadas M, Lopez-Ruiz C, Gerhardt DJ, Sebe A, Grabundzija I, Merkert S, Gerdes P, Pulgarin JA, Bock A, Held U, Witthuhn A, Haase A, Sarkadi B, Löwer J, Wolvetang EJ, Martin U, Ivics Z, Izsvák Z, Garcia-Perez JL, Faulkner GJ, Schumann GG (2016). Reprogramming triggers endogenous L1 and Alu retrotransposition in human induced pluripotent stem cells. Nature Commun..

[225] Livak KJ, Schmittgen TD (2001). Analysis of relative gene expression data using real-time quantitative PCR and the 2(-Delta Delta C(T)) Method. Methods..

[226] Wissing S, Muñoz-Lopez M, Macia A, Yang Z, Montano M, Collins W, Garcia-Perez JL, Moran JV, Greene WC (2012). Reprogramming somatic cells into iPS cells activates LINE-1 retroelement mobility. Hum Mol Genet..

[227] Karolchik D, Hinrichs AS, Furey TS, Roskin KM, Sugnet CW, Haussler D, Kent WJ (2004). The UCSC Table Browser data retrieval tool. Nucleic Acids Res..

[228] Katoh K, Rozewicki J, Yamada KD. MAFFT online service: multiple sequence alignment, interactive sequence choice and visualization. Brief Bioinform. 2017;10.1093/bib/bbx108. 10.1093/bib/bbx108 PMC6781576 28968734

[229] Kearse M, Moir R, Wilson A, Stones-Havas S, Cheung M, Sturrock S, Buxton S, Cooper A, Markowitz S, Duran C, Thierer T, Ashton B, Meintjes P, Drummond A (2012). Geneious Basic: an integrated and extendable desktop software platform for the organization and analysis of sequence data. Bioinformatics..

[230] Arokium H, Kamata M, Kim S, Kim N, Liang M, Presson AP, Chen IS (2014). Deep sequencing reveals low incidence of endogenous LINE-1 retrotransposition in human induced pluripotent stem cells. PLoS One..

[231] Langmead B, Salzberg SL (2012). Fast gapped-read alignment with Bowtie 2. Nat Methods..

[232] Robinson MD, McCarthy DJ, Smyth GK (2010). edgeR: a Bioconductor package for differential expression analysis of digital gene expression data. Bioinformatics..

[233] Dobin A, Davis CA, Schlesinger F, Drenkow J, Zaleski C, Jha S, Batut P, Chaisson M, Gingeras TR (2013). STAR: ultrafast universal RNA-seq aligner. Bioinformatics..

[234] Anders S, Huber W (2010). Differential expression analysis for sequence count data. Genome Biol..

[235] Kim D, Langmead B, Salzberg SL (2015). HISAT: a fast spliced aligner with low memory requirements. Nat Methods..

[236] Quinlan AR, Hall IM (2010). BEDTools: a flexible suite of utilities for comparing genomic features. Bioinformatics..

[237] Law CW, Chen Y, Shi W, Smyth GK (2014). voom: Precision weights unlock linear model analysis tools for RNA-seq read counts. Genome Biol.

[238] Love MI, Huber W, Anders S (2014). Moderated estimation of fold change and dispersion for RNA-seq data with DESeq2. Genome Biol..

