# Supplementary material for: Properties of LINE-1 proteins and repeat element expression in the context of amyotrophic lateral sclerosis
Source: Mob DNA. 2018 Dec 15;9:35. doi: 10.1186/s13100-018-0138-z (PMC6295051; doi:10.1186/s13100-018-0138-z)
Supplement: Supplementary file 1 — Figure S1. Patterns of L1 ORF1p expression in various cell lines using multiple antibodies for detection. Figure S2. Frequency of polymorphisms and DNA methylation at the L1 ORF1p R159 residue. Figure S3. ORF1p does not contain a CRM1-dependent nuclear export signal. Figure S4. Cell culture toxicity assays. Figure S5. Overexpression or knockdown of TDP-43 protein does not alter L1 expression. Figure S6. Methylation analyses of the CpG island of the 5' UTR promoter of endogenous L1 elements show effects of altered levels of TDP-43. Figure S7. Expression levels of L1 (and Alu TEs determined by RT-qPCR. Figure S8. Representative gels showing expression of ORF1p in normal and ALS-associated tissues determined by Western blotting. Figure S9. TE locus-specific analyses of the GSE67196 RNA-Seq dataset [171]. (PDF 25088 kb) [file 13100_2018_138_MOESM1_ESM.pdf]

Properties of LINE-1 proteins and repeat element expression in the context of amyotrophic lateral sclerosis

Gavin C. Pereira[1], Laura Sanchez[2], Paul M. Schaughency[3], Alejandro Rubio-Roldán[2], Jungbin A. Choi[1], Evarist Planet[4], Ranjan Batra[5], Priscilla Turelli[4], Didier Trono[4], Lyle W. Ostrow[6], John Ravits[5], Haig H. Kazazian[1], Sarah J. Wheelan[3], Sara R. Heras[2,7], Jens Mayer[8], Jose Luis García-Pérez[2,9], John L. Goodier[1*].

1. McKusick-Nathans Institute of Genetic Medicine, Johns Hopkins University School of Medicine, Baltimore, Maryland, USA.
2. GENYO. Centre for Genomics and Oncological Research: Pfizer, University of Granada, Andalusian Regional Government, Granada, Spain.
3. Oncology Center-Cancer Biology, Johns Hopkins University School of Medicine, Baltimore, Maryland, USA.
4. School of Life Sciences, École Polytechnique Fédérale de Lausanne (EPFL), Lausanne CH-1015, Switzerland.
5. Department of Neurosciences, School of Medicine, University of California at San Diego, USA.
6. Neuromuscular Division, Johns Hopkins University School of Medicine, Baltimore, Maryland, USA.
7. Department of Biochemistry and Molecular Biology II, Faculty of Pharmacy, University of Granada, Spain.
8. Department of Human Genetics, Medical Faculty, University of Saarland, Homburg/Saar, Germany.
9. MRC Human Genetics Unit, Institute of Genetics and Molecular Medicine (IGMM), University of Edinburgh, Western General Hospital, Edinburgh, United Kingdom.

* Corresponding author: jgoodier@jhmi.edu

Additional file 1: Supplemental Figures

Figure S1. Patterns of L1 ORF1p expression in various cell lines using multiple antibodies for detection.

Figure S2. Frequency of polymorphisms and DNA methylation at the L1 ORF1p R159 residue.

Figure S3. ORF1p does not contain a CRM1-dependent nuclear export signal.

Figure S4. Cell culture toxicity assays.

Figure S5. Overexpression or knock-down of TDP-43 protein does not alter L1 expression.

Figure S6. Methylation analyses of the CpG island of the 5' UTR promoter of endogenous L1 elements show effects of altered levels of TDP-43.

Figure S7. Expression levels of L1 and Alu determined by RT-qPCR.

Figure S8. Representative gels showing expression of ORF1p in normal and ALS-associated tissues determined by Western blotting.

Figure S9. TE locus-specific analyses of the GSE67196 RNA-Seq dataset.

SUPPLEMENTAL FIGURES

Figure S1. Patterns of L1 ORF1p expression in various cell lines using multiple antibodies for detection. **A)** Western blot analysis of expression in human cell lines using the monoclonal α-4H1-ORF1 antibody. Left: following antibody probing, the membrane was stained with ponceau S to show transferred protein. **B)** Detection of endogenous ORF1p in cytoplasmic granules of 2102Ep cells with rabbit monoclonal α-JH73-ORF1 antibody [177]. **C)** In human neuroblastoma SH-SY5Y cells, ORF1p aggregates are predominantly seen in neurite outgrowths not in the cytoplasm proper. **D)** Detection of endogenous ORF1p in the cytoplasm of HEK 293T cells using the rabbit polyclonal α-AH40.1-ORF1 antibody [68]. **E)** Cytoplasmic granule, nucleolar and perinuclear concentration of endogenous ORF1p in 2102Ep cells detected by α-AH40.1-ORF1. **F)** Only a small percentage of endogenous ORF1p cytoplasmic granules colocalize with p-bodies detected by α-p70 S6 kinase antibody, which recognizes HEDLS/EDC4 (marked by arrows, [220]). **G-I)** Some ORF1p cytoplasmic granules colocalize with RFP-tagged LC3 but not with endogenous autophagic proteins ATG12 or ATG16L in untreated 2102Ep cells.

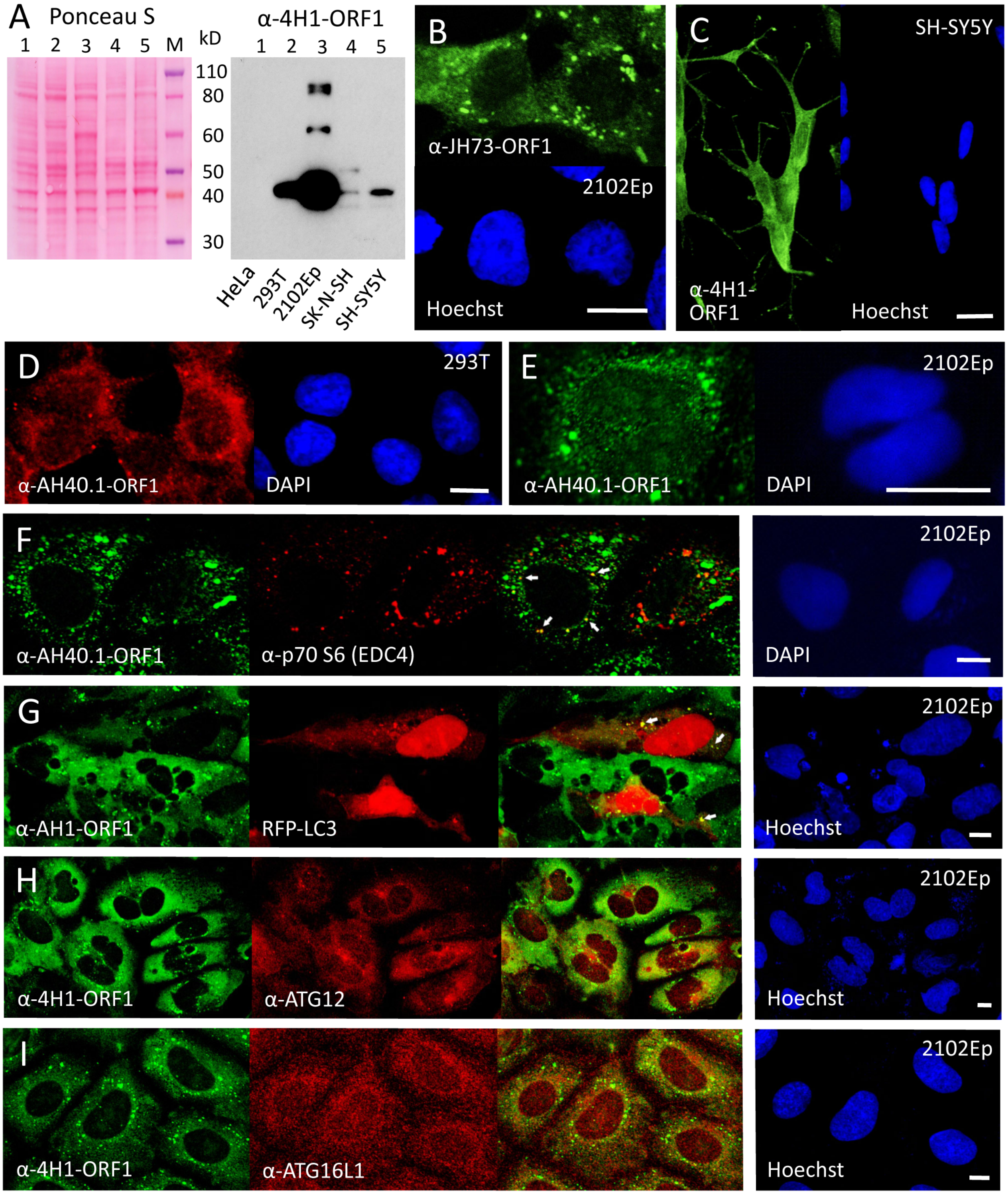

A
Ponceau S
1 2 3 4 5 M
kD
110
80
60
50
40
30
α-4H1-ORF1
1 2 3 4 5
HeLa
293T
2102Ep
SK-N-SH
SH-SY5Y
B
α-JH73-ORF1
2102Ep
Hoechst
C
SH-SY5Y
α-4H1-ORF1
Hoechst
D
293T
α-AH40.1-ORF1
DAPI
E
2102Ep
α-AH40.1-ORF1
DAPI
F
α-AH40.1-ORF1
α-p70 S6 (EDC4)
2102Ep
DAPI
G
α-AH1-ORF1
RFP-LC3
2102Ep
Hoechst
H
α-4H1-ORF1
α-ATG12
2102Ep
Hoechst
I
α-4H1-ORF1
α-ATG16L1
2102Ep
Hoechst

Fig. S1

Figure S2. Frequency of polymorphisms and DNA methylation at the L1 ORF1p R159 residue. **A)** Based on L1 elements compiled in L1Base2 [13], we examined the frequency of non-synonymous nucleotide changes within the R159 codon and resulting amino acids. FLI-L1s: L1s with intact ORF1 and ORF2; ORF2-L1s: L1s with intact ORF2 but disrupted ORF1; FLnI-L1s: non-intact L1s>4500 nucleotides in length. For each category, observed total numbers and percentages of codons for arginine (R) and the most frequently occurring codons for histidine (H), cysteine (C) and proline (P) are given. Also shown for the FLnI-L1s dataset is the frequency of a codon for glycine (G) (see the main paper text for details). A minority of L1 sequences (~2.7%) displayed other codons, very likely because of higher sequence divergence within the R159 region resulting in unreliable alignments and thus unreliable prediction of the R159 codon. **B)** Bisulfite conversion methylation analysis by QUantification tool for Methylation Analysis (QUMA; [223]) software of 9 CpGs within a 436-nt stretch of ORF1 shows no preference for conversion of the R159 CpG. Left: percent methylation of CpG residues within this stretch. CpG residues are numbered according to element L1-RP (accession number AF148856.1). Right: the individual methylation status of 26 L1 amplicon sequences. Open circles, closed circles and crosses represent unmethylated, methylated and mutated CpG positions, respectively.

A

		FLI-L1s		ORF2-L1s		FLnI-L1s	
AA	Codon	#	%	#	%	#	%
**Arg / R**	**CGT**	**139**	**95.2**	**85**	**88.5**	**3541**	**55.8**
	**CGC**					**25**	**0.4**
	**CGA**					**17**	**0.3**
	**CGG**					**2**	**0.03**
**His / H**	**CAT**	**4**	**2.7**	**7**	**7.3**	**1507**	**23.8**
	**CAC**					**14**	**0.2**
**Cys / C**	**TGT**	**2**	**1.4**	**4**	**4.2**	**949**	**15.0**
	**TGC**					**16**	**0.3**
**Pro / P**	**CCT**	**1**	**0.7**			**62**	**1.0**
	**CCG**					**1**	**0.02**
**Gly / G**	**GGT**					**30**	**0.5**
**Other**						**182**	**2.9**
**TOTAL**		**146**	**100**	**96**	**100**	**6346**	**100**

B

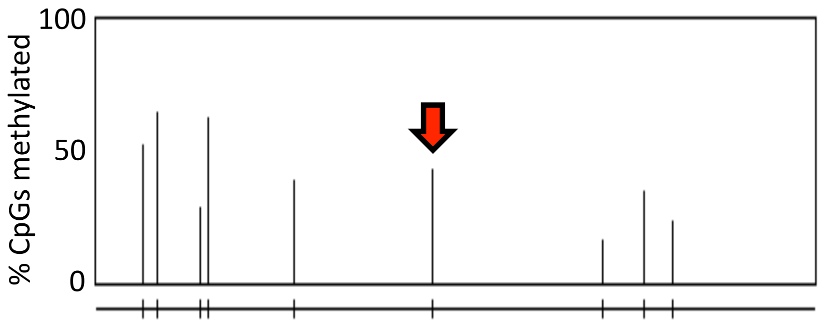

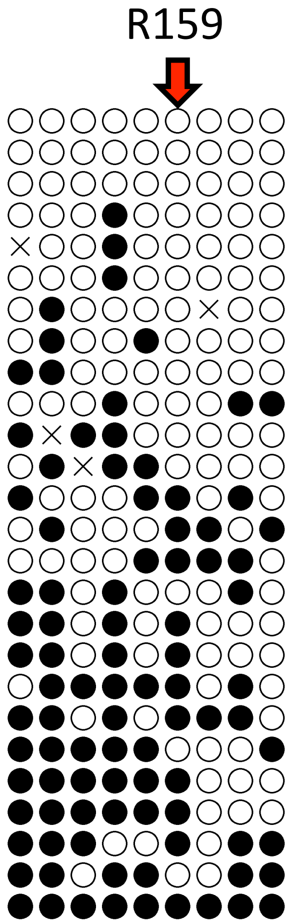

R159

CpG Position	1198	1207	1234	1239	1294	1381	1488	1514	1532	Total
Me-CpG	52%	64%	28%	62%	39%	42%	16%	35%	23%	40%

Fig. S2

Figure S3. ORF1p does not contain a CRM1-dependent nuclear export signal. **A)** Treatment of 2102Ep cells with 30 ng/ml leptomycin B for 4 hours or overnight does not obviously alter the localization pattern of endogenous ORF1p. Patient sera-derived α-ANA-N marks nucleoli. **B,C)** However, LMB efficiently inhibits nuclear export of control proteins, endogenous cyclin B1 (B) and exogenously expressed MK2 T205/317E mutant protein (C) [111]. **D)** Fusing the putative ORF1p nuclear export signal LKELLKEAL (aa 313-321) to EGFP does not visibly increase accumulation of EGFP protein in the cytoplasm. **E)** Mutating the putative ORF1p nuclear export signal sequence LKELLKEAL in ORF1-EGFP-L1-RP (ORF1-EGFP-L1-RP-LKEAAAAAL) does not visibly increase accumulation of ORF1p in nuclei. Size bars are 10 µm.

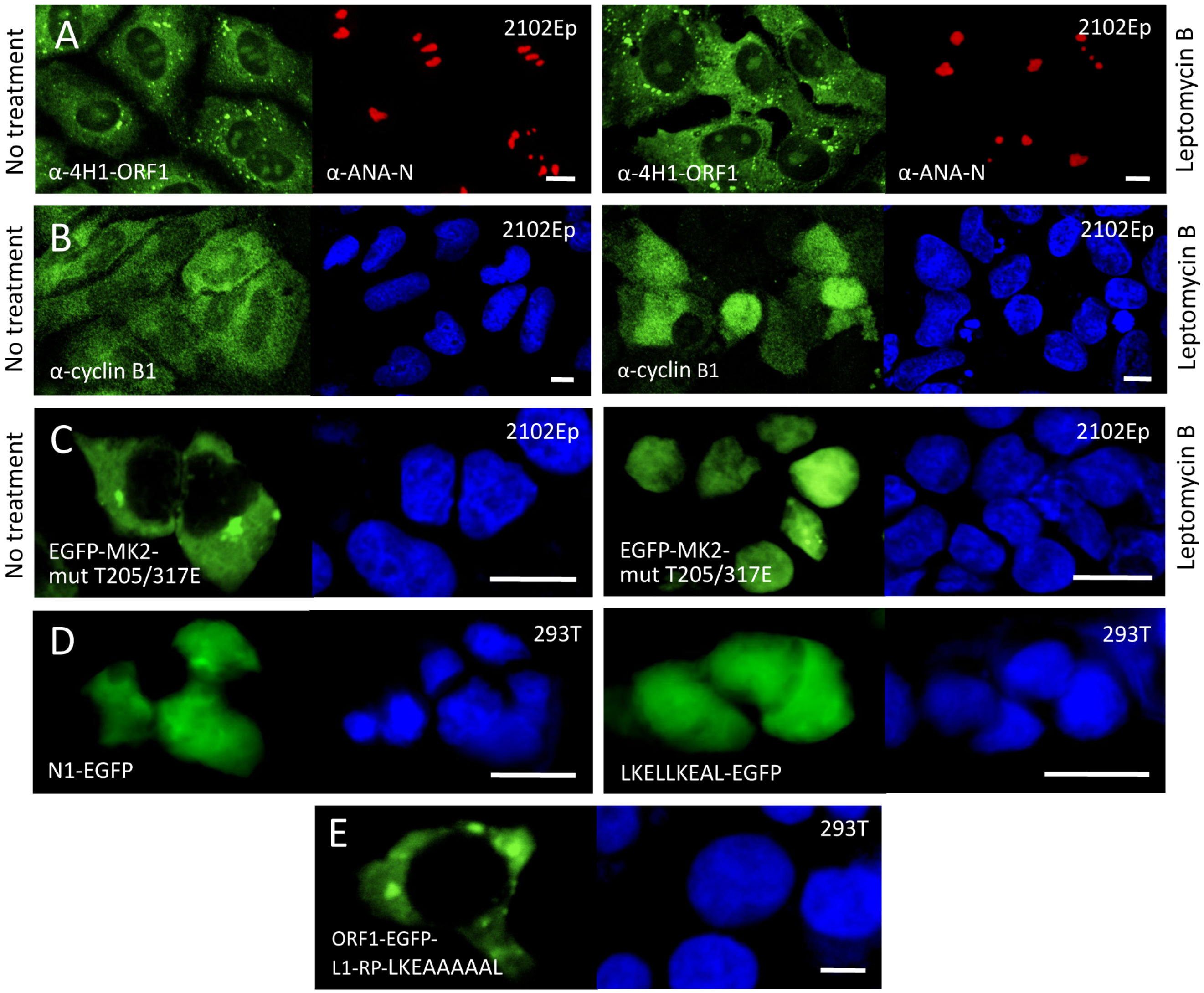
A
B
C
D
E
No treatment
Leptomycin B
α-4H1-ORF1
α-ANA-N
2102Ep
α-cyclin B1
EGFP-MK2-
mut T205/317E
N1-EGFP
LKELLKEAL-EGFP
293T
ORF1-EGFP-
L1-RP-LKEAAAAAL

Fig. S3

Figure S4. Cell culture toxicity assays. **A)** HEK 293T cells, transfected in five wells with each plasmid tested in the retrotransposition assay of Figure 5B, were stained on day 4 post-transfection with trypan blue and live-dead cells were counted using a Cellometer Auto T4 Cell Viability Counter (Nexcelom Bioscience). pcDNA6/myc-His B and pcDNA5 FRT/TO empty vectors were included as controls (lighter bars). **B-E)** Overexpression of TDP-43 does not induce cell toxicity. **B)** To assess cytotoxicity, TDP-43-V5 wild-type and mutant constructs or pcDNA6/myc-His B empty vector were cotransfected in HeLa cells in quadruplicate wells together with pcDNA3, a vector that expresses the neomycin phosphotransferase gene. Following G418 selection for 18 days, cells were fixed, stained, and antibiotic-resistant colonies were counted. Results are normalized to pcDNA6/myc-His B vector (lighter bar). **C)** Myc-TDP-43, V5-ANG or pcDNA3 empty vector were cotransfected in HeLa cells together with pcDNA6/myc-His B vector and cells were selected on blasticidin antibiotic. Cells were fixed and stained and colonies were counted as above. Results are normalized to pcDNA3 vector (lighter bar). **D)** HEK 293T cells transfected in quadruplicate with plasmids as indicated were stained on day 4 post-transfection with trypan blue and live-dead cells were counted. **E)** Results of MultiTox-Fluor Multiplex Cytotoxicity assay (Promega) showing no cell toxicity caused by overexpression of TDP-43 or FLAG-tagged FUS. Constructs were transfected in 96-well plates (5 wells each) and assayed at day 3. The histogram shows ratios of live to dead cell readings normalized to empty vector control (lighter bar). A-E) single biological replicates are shown. **F)** ALS-associated TBK1 mutations fail to rescue inhibition of cell culture retrotransposition caused by V5-tagged wild-type TBK1 protein. n=number of biological replicates.

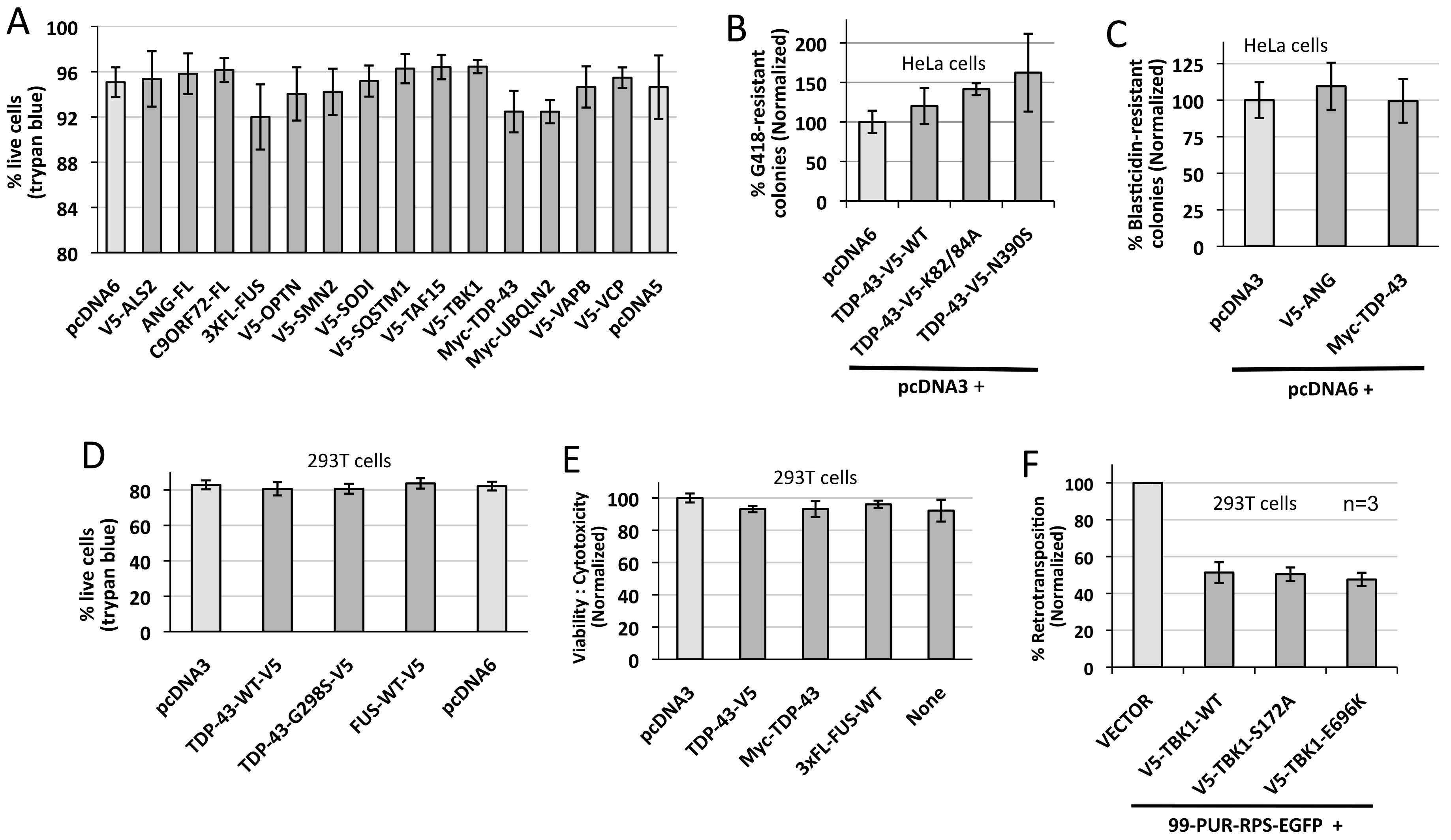

A
% live cells (trypan blue)
100
96
92
88
84
80
pcDNA6
V5-ALS2
ANG-FL
C9ORF72-FL
3XFL-FUS
V5-OPTN
V5-SMN2
V5-SODI
V5-SQSTM1
V5-TAF15
V5-TBK1
Myc-TDP-43
Myc-UBQLN2
V5-VAPB
V5-VCP
pcDNA5
B
% G418-resistant colonies (Normalized)
HeLa cells
200
150
100
50
0
pcDNA6
TDP-43-V5-WT
TDP-43-V5-K82/84A
TDP-43-V5-N390S
pcDNA3 +
C
% Blasticidin-resistant colonies (Normalized)
HeLa cells
125
100
75
50
25
0
pcDNA3
V5-ANG
Myc-TDP-43
pcDNA6 +
D
293T cells
% live cells (trypan blue)
80
60
40
20
0
pcDNA3
TDP-43-WT-V5
TDP-43-G298S-V5
FUS-WT-V5
pcDNA6
E
293T cells
Viability : Cytotoxicity (Normalized)
100
80
60
40
20
0
pcDNA3
TDP-43-V5
Myc-TDP-43
3xFL-FUS-WT
None
F
% Retrotransposition (Normalized)
293T cells
n=3
100
80
60
40
20
0
VECTOR
V5-TBK1-WT
V5-TBK1-S172A
V5-TBK1-E696K
99-PUR-RPS-EGFP +

Fig. S4

Figure S5. Overexpression or knockdown of TDP-43 protein does not alter L1 expression. **A)** HEK 293T cells were transfected with three different scrambled siRNAs (lanes 1,3,5) or siRNAs directed against TDP-43 (lanes 2 and 4) and tested for their effects on cell culture retrotransposition of 99-PUR-RPS-EGFP. Western blots show that esiTARDBP and si2TDP43 siRNAs decreased endogenous TDP-43 protein levels over 70% in HEK 293T cells but did not affect levels of endogenous L1 ORF1 protein (top) or frequency of L1 retrotransposition (bottom). **B,C)** Ectopic expression of tagged TDP-43 constructs fails to alter (B) levels of endogenous ORF1p or (C) FLAG-tagged ORF1p coexpressed from construct pc-L1-1FH in HEK 293T cells. **D)** Overexpression of TDP-43 protein has little effect on L1 endogenous RNA levels in 293T cells as determined by RT-PCR. Shown are results for two vector control samples (pcDNA3 and pcDNA5) and V5- or Myc-tagged TDP-43 expressing samples. Primer pairs amplified DNA fragments from the 5' UTR or ORF1 regions of endogenous L1s and from the actin beta gene. Water only and reverse transcriptase minus control reactions are also shown.

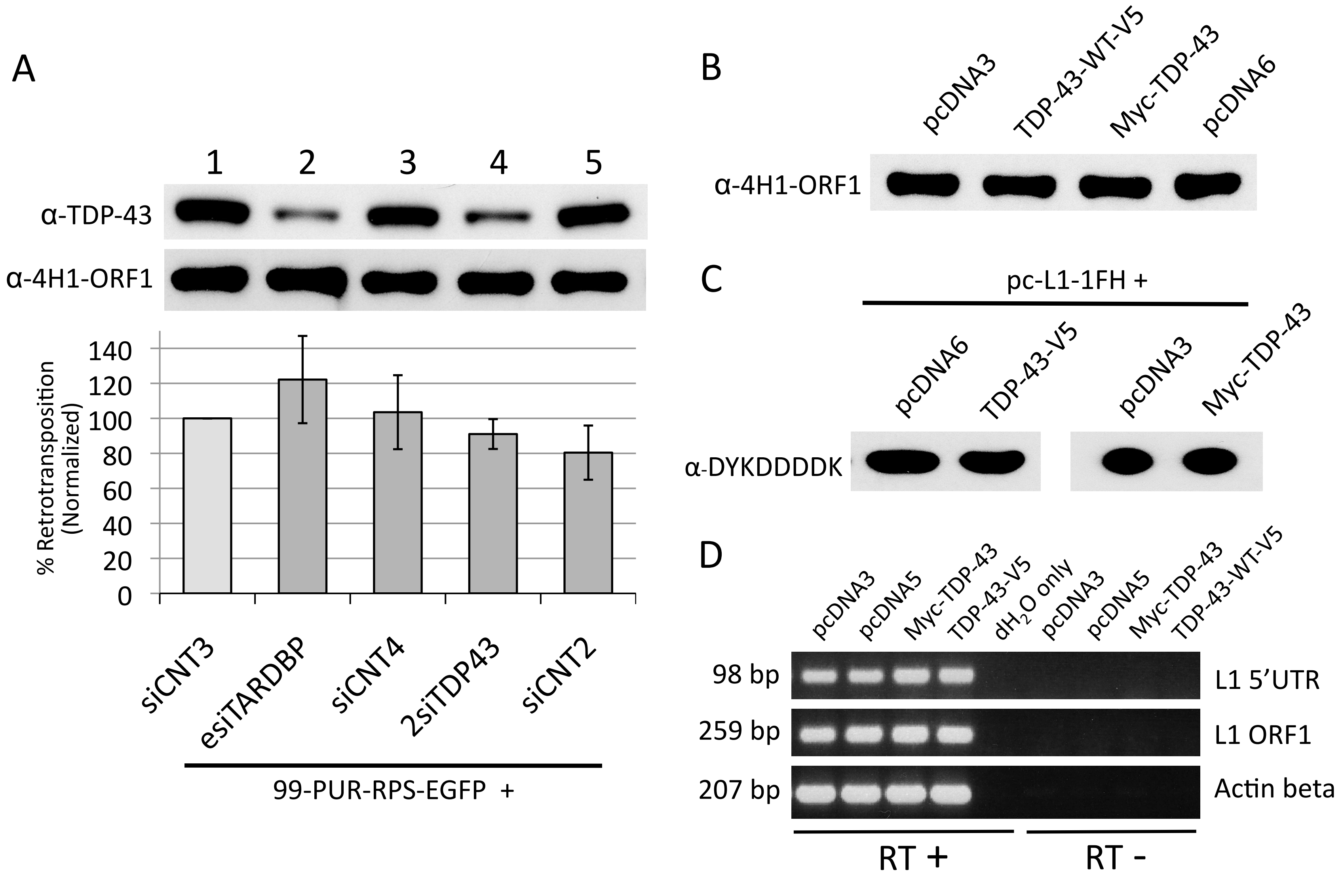

A
1
2
3
4
5
α-TDP-43
α-4H1-ORF1
% Retrotransposition (Normalized)
140
120
100
80
60
40
20
0
siCNT3
esiTARDBP
siCNT4
2siTDP43
siCNT2
99-PUR-RPS-EGFP +
B
pcDNA3
TDP-43-WT-V5
Myc-TDP-43
pcDNA6
α-4H1-ORF1
C
pc-L1-1FH +
pcDNA6
TDP-43-V5
pcDNA3
Myc-TDP-43
α-DYKDDDDK
D
pcDNA3
pcDNA5
Myc-TDP-43
TDP-43-V5
dH2O only
pcDNA3
pcDNA5
Myc-TDP-43
TDP-43-WT-V5
98 bp
259 bp
207 bp
L1 5'UTR
L1 ORF1
Actin beta
RT +
RT -

Fig. S5

Figure S6. Methylation analyses of the CpG island of the 5' UTR promoter of endogenous L1 elements show effects of altered levels of TDP-43. **A)** The individual methylation status of 18 L1 sequences in the presence (right) or absence (left) of ectopically expressed TDP-43-V5-WT protein. Open circles, closed circles, and crosses represent unmethylated, methylated, and mutated CpG positions, respectively. Average percent CpG methylation was significantly elevated in the presence of elevated TDP-43 protein (* $p<0.05$; ** $p<0.01$; *** $p<0.001$, Fisher's Exact Test as determined by QUMA). One replicate experiment is shown. **B,C)** The individual methylation status of 15 L1 sequences in HEK 293T cells reduced for TDP-43 levels by transfection of siRNAs esiTARDBP or 2siTDP43 (see Figure S5A). Both siRNAs caused a significant increase in CpG methylation of L1 promoters. CpG residues are numbered according to the element L1-RP. One replicate experiment each is shown. **D)** Expression of V5- or FLAG-tagged TDP-43 fails consistently to alter activity in HEK 293T cells of luciferase constructs containing either the sense or antisense L1-RP promoter as determined by the Dual-Glo Luciferase Assay System (Promega). Promoter activity from the antisense strand of the human L1 5' UTR is considerably weaker than from the sense strand [6]. L1 promoter constructs were previously described [A]. pGL3-Basic vector (Promega) lacks eukaryotic promoter and enhancer sequences. The pGL3-Control vector (Promega) contains SV40 promoter and enhancer sequences. Results are normalized to the L1-Sense cotransfected pcDNA3 vector control.

A. Yang N, Kazazian HH. An important role for RUNX3 in human L1 transcription and retrotransposition. Nucleic Acids Res. 2003;31(16):4929-40.

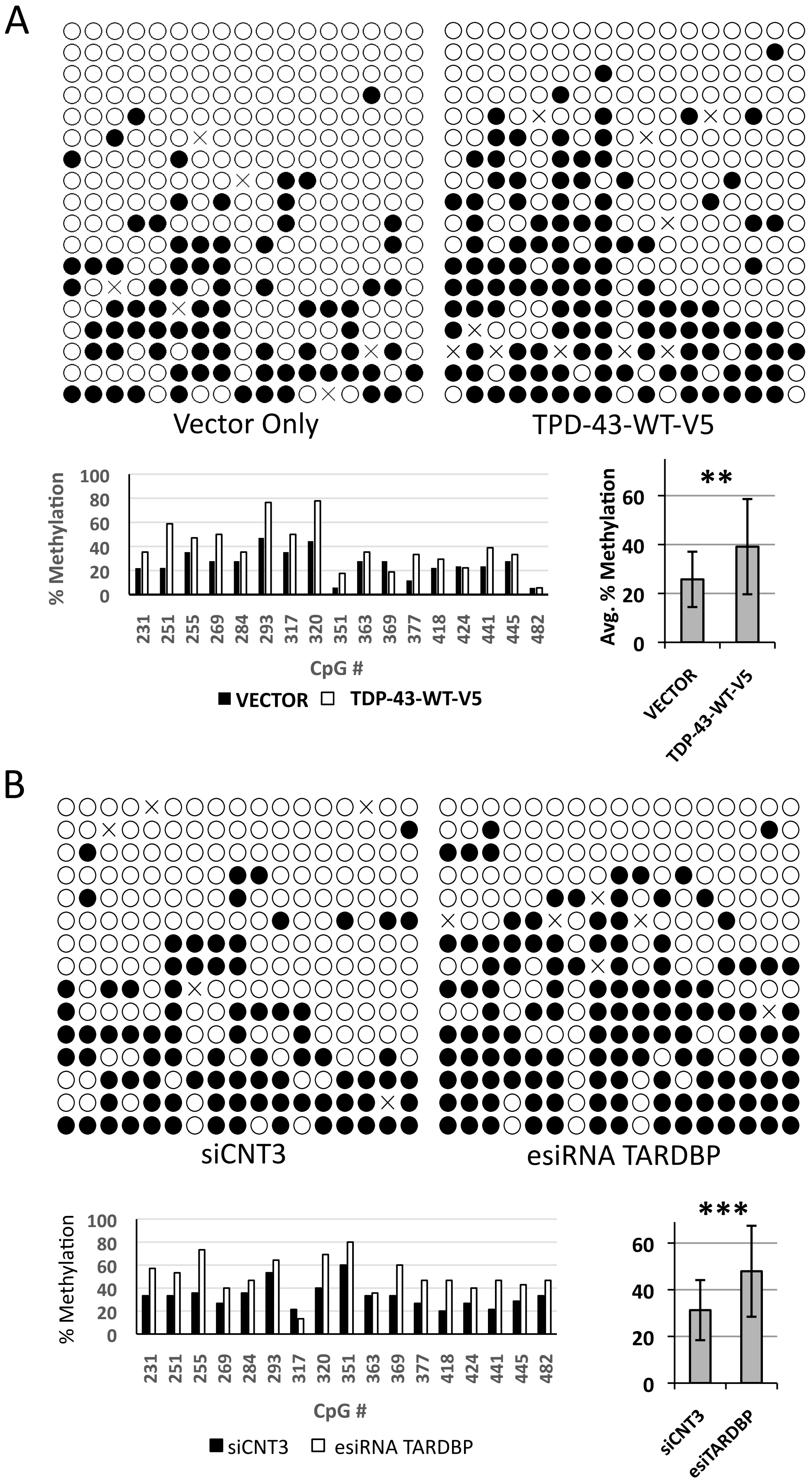
A
Vector Only
TPD-43-WT-V5
% Methylation
CpG #
231 251 255 269 284 293 317 320 351 363 369 377 418 424 441 445 482
VECTOR
TDP-43-WT-V5
Avg. % Methylation
**
B
siCNT3
esiRNA TARDBP
% Methylation
CpG #
siCNT3
esiRNA TARDBP
esiTARDBP
***

Fig. S6

C

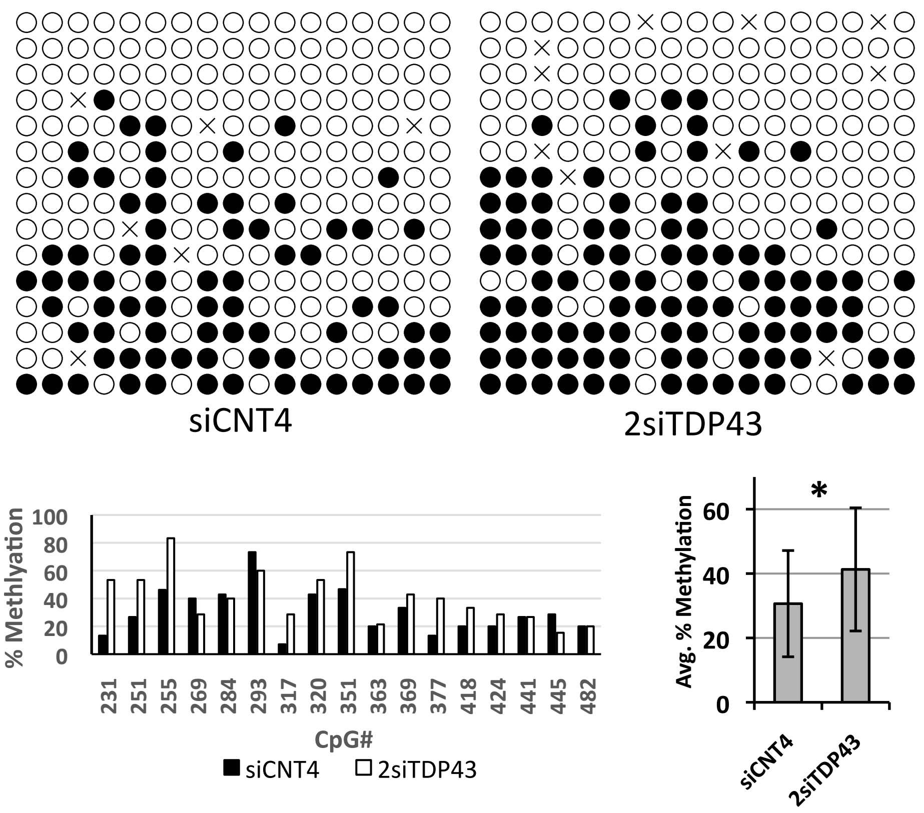
siCNT4
2siTDP43
% Methlyation
100
80
60
40
20
0
231 251 255 269 284 293 317 320 351 363 369 377 418 424 441 445 482
CpG#
siCNT4
2siTDP43
Avg. % Methylation
*
60
40
20
0
siCNT4
2siTDP43

D

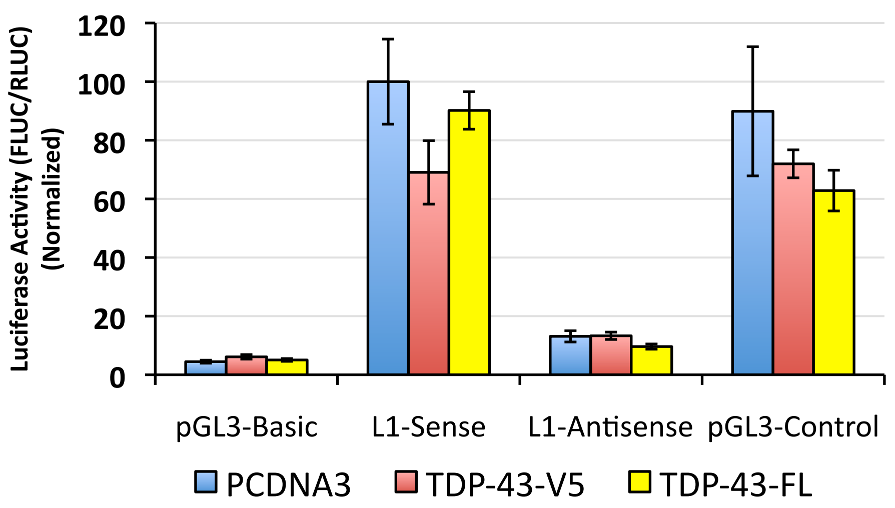
Luciferase Activity (FLUC/RLUC)
(Normalized)
120
100
80
60
40
20
0
pGL3-Basic
L1-Sense
L1-Antisense
pGL3-Control
PCDNA3
TDP-43-V5
TDP-43-FL

Fig. S6

Figure S7. Expression levels of L1 **(A,B)** and Alu **(C,D)** TEs determined by RT-qPCR. Overall transcript levels were measured in samples from various tissue types from ALS patients and non-affected controls employing a previously established strategy [146]. Hippocampal samples were available from ALS patients only. The graphs on the left depict normalized transcript levels determined for individual samples grouped by tissue type and disease state. The right graphs depict averaged normalized transcript levels for those samples and tissue types. Alu and L1 transcript levels are normalized to H9-hESC levels (set to 1), and HEF and HeLa cell results are included in the graphs. Standard errors are indicated for each tissue type. Note that much more variable and often higher transcript levels were determined for cerebellum samples. Only AluS occipital cortex samples showed significantly different transcript levels for ALS patients compared with controls ($p=0.02$; Student's t-test).

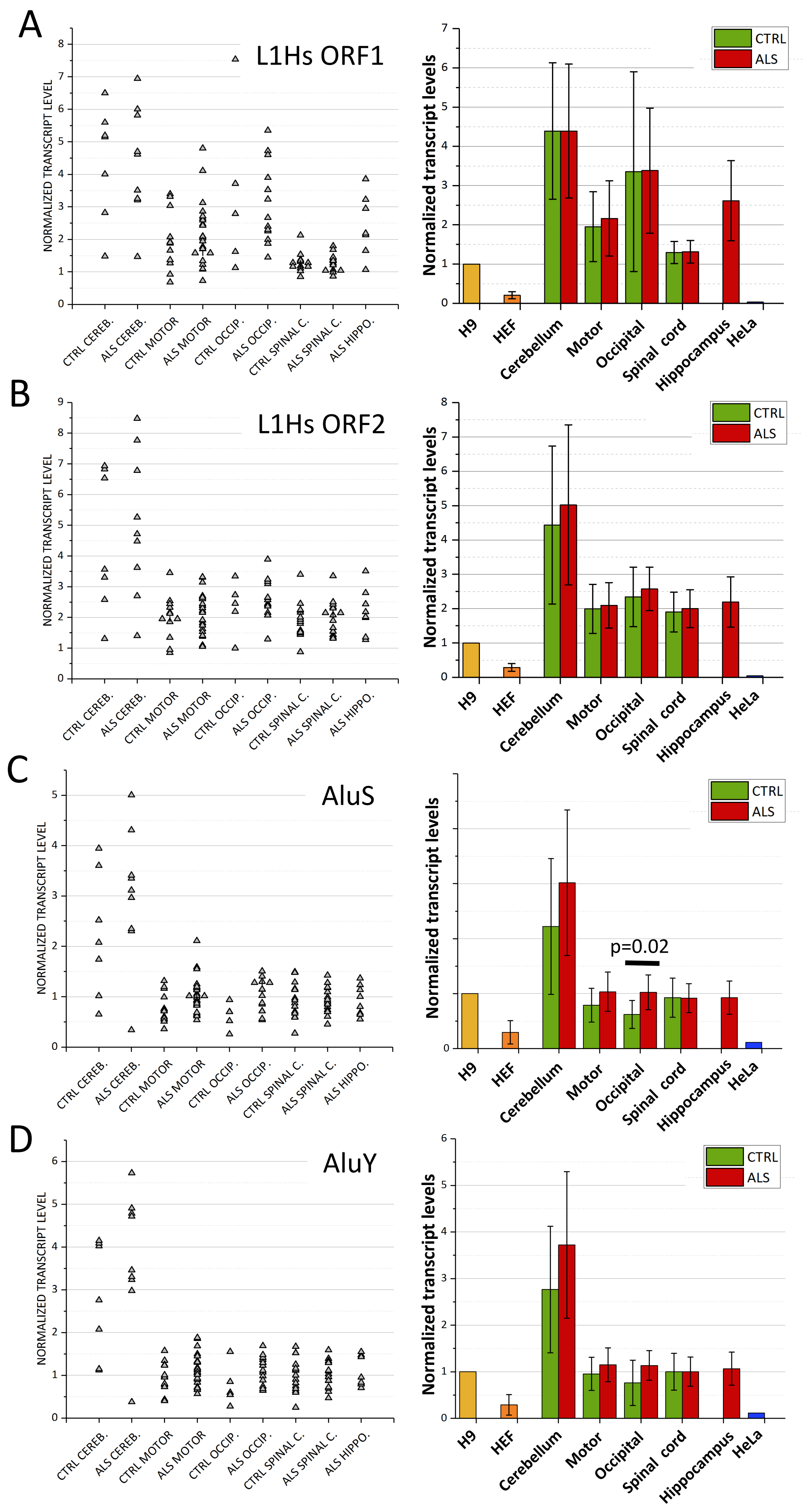
A
L1Hs ORF1
NORMALIZED TRANSCRIPT LEVEL
Normalized transcript levels
CTRL
ALS
CTRL CEREB.
ALS CEREB.
CTRL MOTOR
ALS MOTOR
CTRL OCCIP.
ALS OCCIP.
CTRL SPINAL C.
ALS SPINAL C.
ALS HIPPO.
H9
HEF
Cerebellum
Motor
Occipital
Spinal cord
Hippocampus
HeLa
B
L1Hs ORF2
C
AluS
p=0.02
D
AluY

Fig. S7

Figure S8. Representative gels showing expression of ORF1p in normal and ALS-associated tissues determined by Western blotting. Fifty µg of 2102Ep cell lysate and whole tissue lysates from **A)** frontal cortex, **B)** cerebellum, **C)** hippocampus, **D)** motor cortex, and **E)** cervical or thoracic spinal cord samples were probed with mouse monoclonal α-4H1-ORF1 antibody (right). Left: following antibody probing, the membrane was stained with ponceau S to show transferred protein. Bands consistent in size with full-length ORF1p are indicated by an arrow in E). **F)** Hippocampal samples were reprobed with rabbit polyclonal α-V14-ORF1 antibody [182]. **G)** Brain and spinal cord samples were also probed with rabbit monoclonal α-JH73-ORF1 antibody [177].

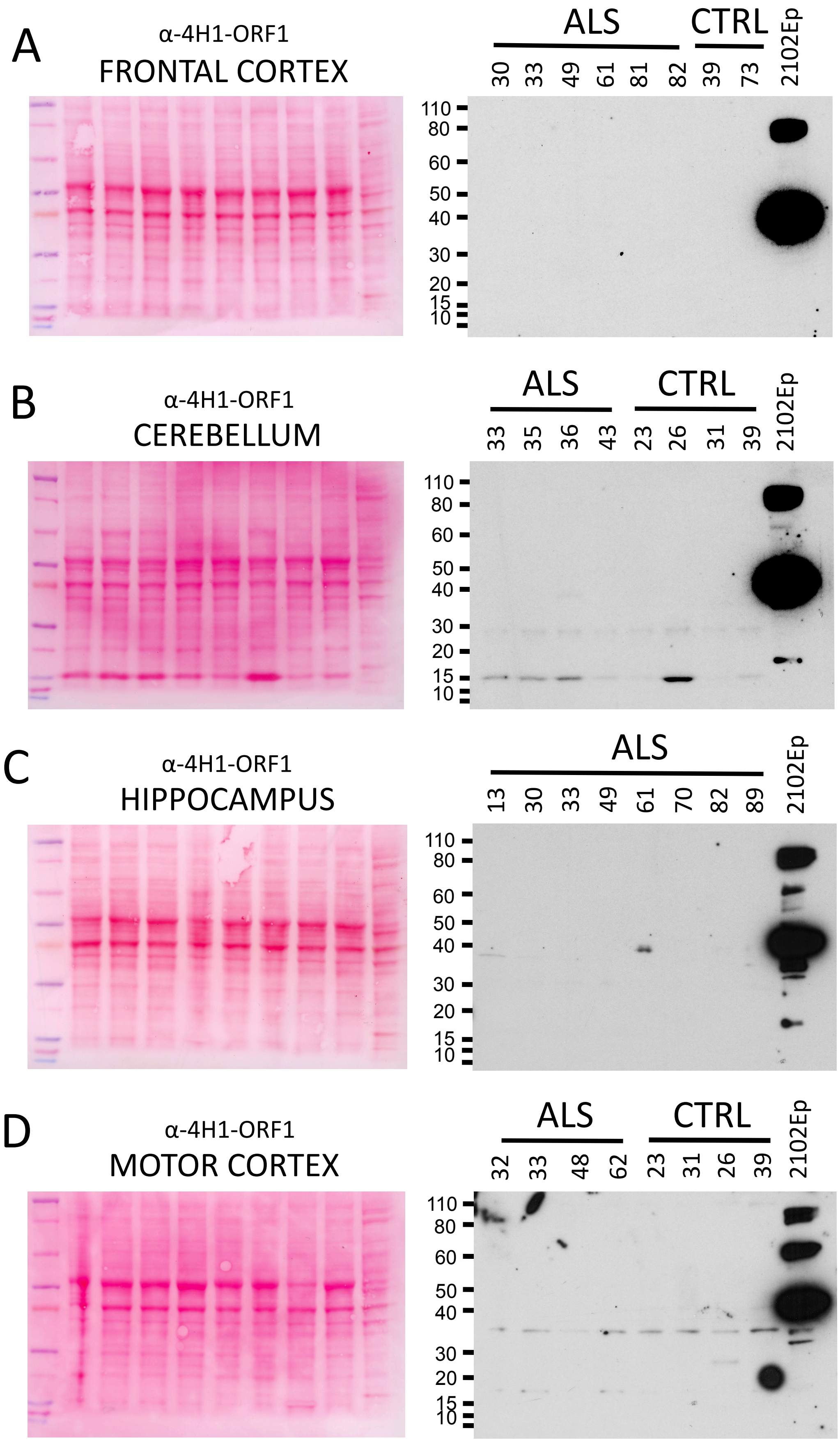
A
α-4H1-ORF1
FRONTAL CORTEX
ALS
CTRL
30 33 49 61 81 82 39 73 2102Ep
110 80 60 50 40 30 20 15 10
B
α-4H1-ORF1
CEREBELLUM
ALS
CTRL
33 35 36 43 23 26 31 39 2102Ep
110 80 60 50 40 30 20 15 10
C
α-4H1-ORF1
HIPPOCAMPUS
ALS
13 30 33 49 61 70 82 89 2102Ep
110 80 60 50 40 30 20 15 10
D
α-4H1-ORF1
MOTOR CORTEX
ALS
CTRL
32 33 48 62 23 31 26 39 2102Ep
110 80 60 50 40 30 20 15 10

Fig. S8

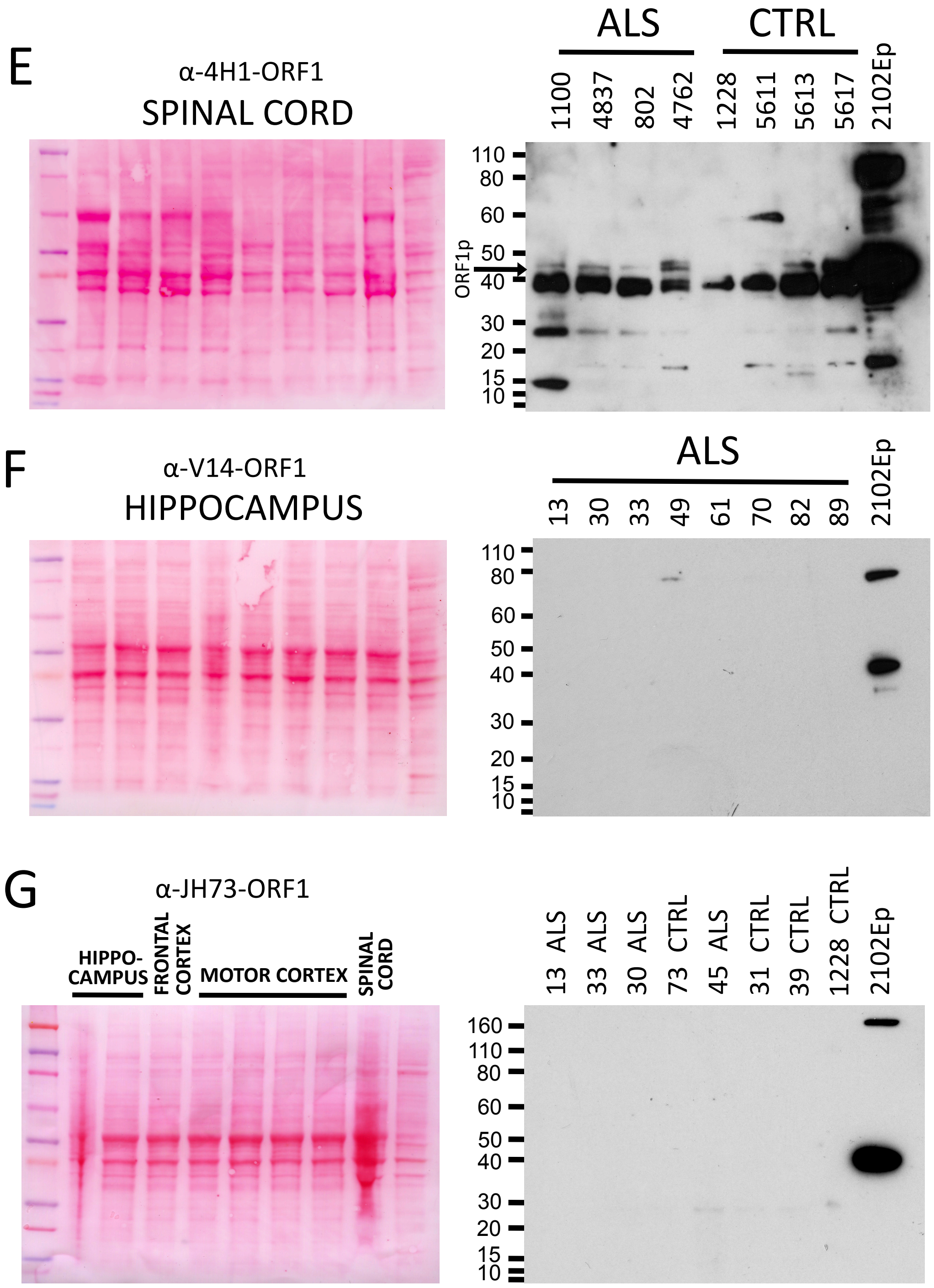
E
α-4H1-ORF1
SPINAL CORD
ALS
CTRL
1100
4837
802
4762
1228
5611
5613
5617
2102Ep
110
80
60
50
40
30
20
15
10
ORF1p
F
α-V14-ORF1
HIPPOCAMPUS
ALS
13
30
33
49
61
70
82
89
2102Ep
110
80
60
50
40
30
20
15
10
G
α-JH73-ORF1
HIPPO-
CAMPUS
FRONTAL
CORTEX
MOTOR CORTEX
SPINAL
CORD
13 ALS
33 ALS
30 ALS
73 CTRL
45 ALS
31 CTRL
39 CTRL
1228 CTRL
2102Ep
160
110
80
60
50
40
30
20
15
10

Fig. S8

Figure S9. TE locus-specific analyses of the GSE67196 RNA-Seq dataset [171]. **A)** Table summary of the numbers of significant DE TE loci identified in the dataset for selected TE-types. All significant data is presented in Table S3. **B)** PCA analyses for TEs of sALS (blue), C9ALS (green), and control (red) samples in cerebellum (left) and frontal cortex (right) samples. Individual patient samples are numbered. Discrete clustering of C9ALS samples is evident for frontal cortex only. **C)** Hierarchical clustering of differentially expressed TE loci in cerebellum (left) and frontal cortex. Each column of the heat map corresponds to a control, sALS, or C9ALS sample, color-coded as shown in the box, right. Each cell shows the raw Z-score of a single TE element color-coded as shown in the key at top left. Shown are the top 5000 endogenous retroelement loci (eres) with the largest standard deviations (SD) between samples.

A

TE TYPE	C9ALS vs sALS					C9ALS vs Ctrl				sALS vs Ctrl			
	TOTAL DE	DE Down	DE Up	TOTAL LOCI	% DE LOCI	TOTAL DE	DE Down	DE Up	% DE LOCI	TOTAL DE	DE Down	DE Up	% DE LOCI
LINE 1	951	86	865	48187	1.97	174	6	168	0.36	24	11	13	0.05
LINE 2	486	53	433	21315	2.28	108	4	104	0.51	17	10	7	0.08
LTR/ERV1	216	24	192	9048	2.39	43	5	38	0.48	6	3	3	0.07
LTR/ERVK	18	3	15	1196	1.51	4	0	4	0.33	1	0	1	0.08
LTR/ERVL-MaLR	247	40	207	13775	1.79	46	5	41	0.33	11	3	8	0.08
LTR/ERVL	106	22	84	6319	1.68	15	1	14	0.24	5	0	5	0.08
SVAs	17	1	16	1240	1.37	4	0	4	0.32	0	0	0	0.00
SINE/Alu	1194	19	1175	67802	1.76	146	8	138	0.22	16	16	0	0.02
SINE/MIR	339	59	280	19593	1.73	64	1	63	0.33	15	1	14	0.08
DNA	307	53	254	19053	1.61	37	2	35	0.19	11	3	8	0.06
?	82					11				3			
TOTAL	3963	360	3521	207528	1.81	652	32	609	0.33	109	47	59	0.06

B

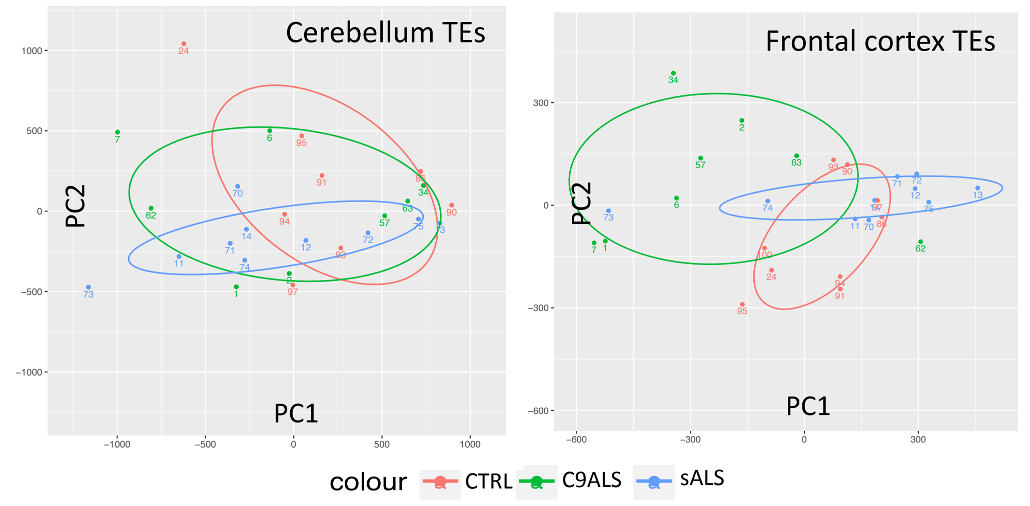

C

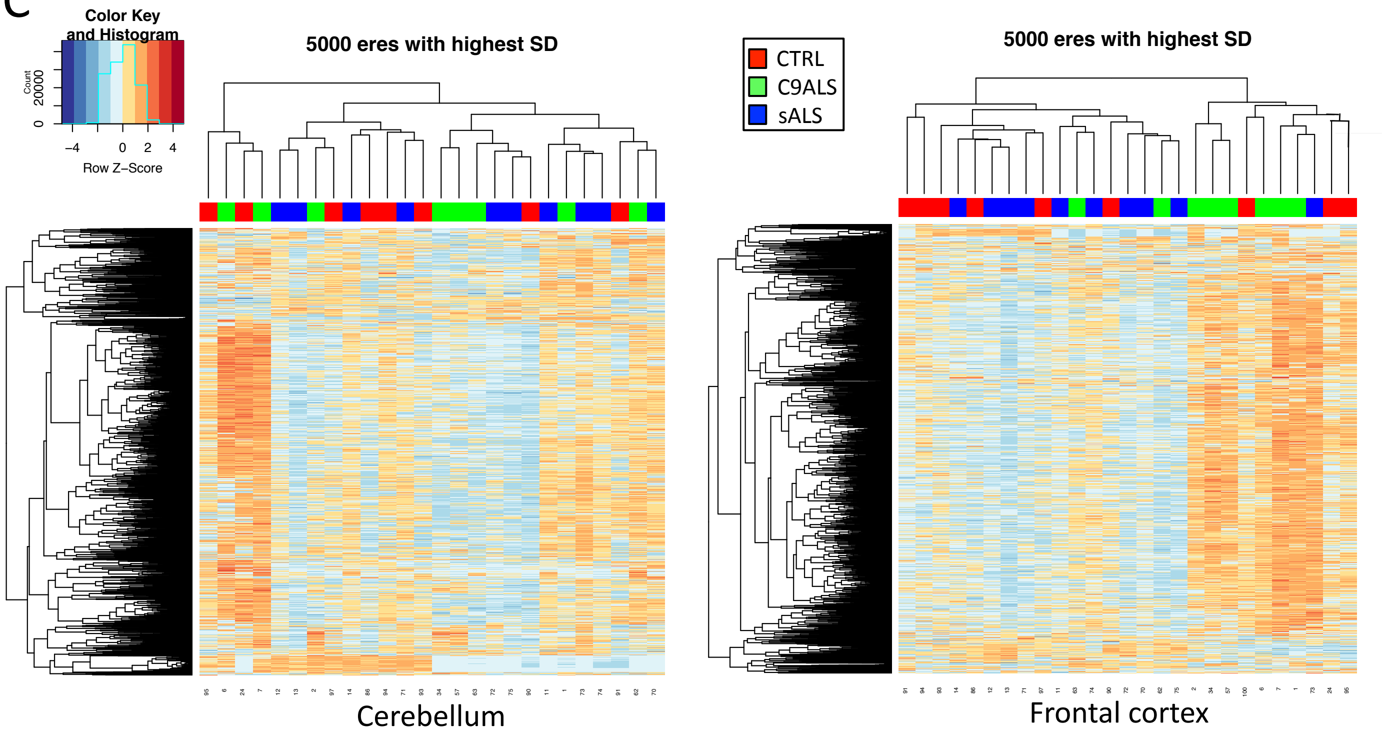

Fig. S9
