# Supplementary material for: Properties of LINE-1 proteins and repeat element expression in the context of amyotrophic lateral sclerosis
Source: Mob DNA. 2018 Dec 15;9:35. doi: 10.1186/s13100-018-0138-z (PMC6295051; doi:10.1186/s13100-018-0138-z)
Supplement: Supplementary file 3 — Table S2. Tissue samples used in this study. (PDF 65 kb) [file 13100_2018_138_MOESM3_ESM.pdf]

Table S2: Tissue samples used in this study

Source	Patient #	Region	Type	Age at Onset	Age at Death	Sex	Race	PMI (hours)	RT-qPCR	Western	RIN	Notes / Cause of Death
JHU	13	MOTOR	ALS	~61	71	M	W	?	Y		N/A	
		OCCIPITAL							Y		8	
		HIPPO							Y		3.3	
UCSD	19	MOTOR	CTRL	N/A	80	F	W	2.5	Y		5.3	Parkinson's disease
		CEREBELLUM							Y	Y	5.5	
UCSD	23	MOTOR	CTRL	N/A	77	M	W	6	Y	Y	9.2	Cardiac failure
		CEREBELLUM							Y	Y	6	
UCSD	26	MOTOR	CTRL	N/A	49	M	W	4	Y	Y	8.9	Cancer / Obesity
		CEREBELLUM							Y	Y	7.7	
JHU	30	MOTOR	ALS	28	44	M	W	26.5	Y		5.1	
		OCCIPITAL							Y		5.3	
		HIPPO							Y		4.4	
UCSD	31	MOTOR	CTRL	N/A	67	M	W	3.5	Y	Y	9.4	Lung cancer
		CEREBELLUM							Y	Y	7.7	
UCSD	32	MOTOR	ALS	69.5	71	M	W	4.5	Y	Y	7.1	
		CEREBELLUM							Y	Y	3	
UCSD	33	MOTOR	ALS	47.5	54	M	W	5	Y	Y	7.9	
		CEREBELLUM							Y	Y	5.2	
JHU	33	MOTOR	ALS	63	65	M	W	15.5	Y		N/A	
		OCCIPITAL							Y		2.7	
		HIPPO							Y	Y	2.3	
		FRONTAL								Y	N/A	
UCSD	35	MOTOR	ALS	68.25	74	F	W	5	Y	Y	9.9	
		CEREBELLUM							Y	Y	8	
UCSD	36	MOTOR	ALS	72	73	M	W	5	Y	Y	9.3	
		CEREBELLUM							Y	Y	5.6	
UCSD	39	MOTOR	CTRL	N/A	77	M	W	2	Y	Y	9.1	Aortic dissection / Multiple system failure
		CEREBELLUM							Y	Y	7.3	
		FRONTAL								Y	N/A	
UCSD	43	MOTOR	ALS	72.25	74	M	W	6	Y		4.7	
		CEREBELLUM							Y	Y	6.3	
JHU	44	MOTOR	CTRL	N/A	90	F	W	9	Y		3.4	Polio
		OCCIPITAL							Y		2.9	
UCSD	45	MOTOR	ALS	64	69	F	W	5	Y	Y	9.1	
		CEREBELLUM							Y	Y	7.9	
UCSD	48	MOTOR	ALS	65.2	67	M	W	6	Y	Y	10	
		CEREBELLUM							Y	Y	7.4	
JHU	49	MOTOR	ALS	62	68	F	W	4	Y		7.9	
		OCCIPITAL							Y		7.5	
		HIPPO							Y		2.7	
		FRONTAL								Y	N/A	
JHU	60	MOTOR	CTRL	N/A	80	M	W	15	Y	Y	6.8	Lewy body dementia / Peripheral neuropathy
		OCCIPITAL							Y		4.5	
UCSD	60	MOTOR	ALS	55	58	F	W	3	Y		10	
		CEREBELLUM							Y	Y	7.8	
JHU	61	MOTOR	ALS	71	72	F	W	12	Y		N/A	
		OCCIPITAL							Y		4.2	
		HIPPO							Y		5.4	
		FRONTAL								Y	N/A	
UCSD	62	MOTOR	ALS	50.3	52	M	W	6	Y	Y	10	
		CEREBELLUM							Y	Y	6.9	
UCSD	64	MOTOR	ALS	44	47	M	W	6.5			9.9	
		CEREBELLUM								Y	N/A	
JHU	70	MOTOR	ALS		63	M	W	6	Y		5.2	OPTN M98K
		OCCIPITAL							Y		5.6	
		HIPPO							Y		6.9	
UCSD	73	MOTOR	CTRL	N/A	74	M	W	5.5	Y		10	Cardiovascular failure
		CEREBELLUM							Y		2.9	
		FRONTAL								Y	N/A	
JHU	74	MOTOR	ALS	46	47	M	W	18.5	Y	Y	6	SOD1 A4V
		OCCIPITAL							Y		3.7	
JHU	82	MOTOR	ALS	63	66	M	W	13	Y		5.2	
		OCCIPITAL							Y		7	
		HIPPO							Y	Y	6.2	
		FRONTAL								Y	N/A	

JHU	83	MOTOR	ALS	44	50	M	B	4.5	Y	Y	8.5	SOD1 N139K
		OCCIPITAL							Y		7.6	
JHU	88	MOTOR	ALS	57	59	M	W	10	Y		3.5	FTD+fALS / C9orf72+
		OCCIPITAL							Y		2.1	
JHU	89	MOTOR	ALS	69	71	F	W	19.5	Y		5.8	
		OCCIPITAL							Y		4.3	
		HIPPO							Y	Y	4.3	
JHU	90	MOTOR	ALS	57	59	F	W	10	Y	Y	6.6	
		OCCIPITAL							Y		7.1	
JHU	91	MOTOR	ALS	50	53	M	W	?	Y	Y	7.9	
		OCCIPITAL							Y		7.6	
JHU	92	MOTOR	ALS	70	92	M	W	9.5	Y	Y	6.6	C9orf72+
		OCCIPITAL							Y		4.5	
GBB	13-06/ 93	MOTOR	CTRL		72	M	W	7	Y		6.6	Diffuse alveolar damage / Pneumonia
		OCCIPITAL							Y		6.3	
GBB	14-06	MOTOR	CTRL		71	F	na	5	Y		4.3	Hypertensive vasculopathy, Lacunar infarcts
		OCCIPITAL							Y		N/A	
GBB	15-09/ 101	MOTOR	CTRL		70	F	na	32	Y		9.9	Indeterminate
		OCCIPITAL							Y		N/A	
UCSD	103	MOTOR	CTRL	N/A	92	F	W	10	Y		9.6	Coronary artery disease / Congestive heart failure
		CEREBELLUM							Y		5	
MTB	145	TSC	CTRL		48.66	M	W	21	Y		5.3	Hypertensive arteriosclerotic cardiovascular disease
MTB	357	CSC	ALS		56.01	F	W	21	Y		8	
MTB	802	CSC	ALS		73.63	F	W	20		Y	8.3	
MTB	884	CSC	ALS		88.40	M	W	24	Y		6.6	
MTB	1100	CSC	ALS		57.19	F	W	14	Y	Y	6.4	
MTB	1131	TSC	CTRL		55.06	M	W	8	Y	Y	8.1	Arteriosclerotic cardiovasuclar disease
MTB	1228	TSC	CTRL		47.35	M	W	13	Y	Y	N/A	Arteriosclerotic cardiovasuclar disease
MTB	1515	CSC	ALS		56.05	F	W	6	Y	Y	2.9	
MTB	4263	CSC	CTRL		61.51	M	W	6	Y		7.8	Cardiac arrest
MTB	4762	CSC	ALS		59.65	M	W	6	Y	Y	2.9	
MTB	4768	CSC	ALS		45.97	M	W	19		Y	N/A	
MTB	4837	CSC	ALS		45.97	F	W	12		Y	N/A	
MTB	4857	CSC	ALS		78.33	F	W	19	Y		8.7	
MTB	5004	CSC	ALS		62.60	F	W	22	Y		7.7	
MTB	5148	CSC	ALS		62.62	F	W	9	Y		2.7	
MTB	5358	TSC	CTRL		54.58	F	W	24	Y		6	Hypertensive cardiovascular disease
MTB	5383	CSC	ALS		60.33	F	W	21	Y	Y	6.6	
MTB	5388	CSC	ALS		61.13	M	W	8	Y	Y	3.9	
MTB	5450	CSC	CTRL		48.82	M	B	6	Y	Y	4.1	Congestive heart failure
MTB	5451	CSC	CTRL		57.28	F	B	20		Y	N/A	
MTB	5452	CSC	CTRL		67.52	M	B	23	Y		6.7	Occlusive pulmonary thromboembolism
MTB	5456	CSC	CTRL		57.84	M	W	16	Y		8	Atherosclerotic cardiovascular disease
MTB	5458	CSC	CTRL		64.15	M	W	13	Y		7.6	Multiple Injuries
MTB	5593	CSC	ALS		70.46	F	W	24	Y	Y	7	
MTB	5595	CSC	ALS		56.12	M	W	16		Y	N/A	
MTB	5604	CSC	CTRL		73.33	F	W	20	Y		5.6	
MTB	5609	CSC	CTRL		54.78	F	W	6	Y		6.1	Atherosclerotic cardiovascular disease
MTB	5613	CSC	CTRL		72.36	M	W	24	Y	Y	6.5	Torso Injuries and compressional asphyxia
MTB	5615	TSC	CTRL		50.49	M	W	19		Y	N/A	Acute alcohol intoxication
MTB	5617	CSC	CTRL		59.91	M	W	10	Y	Y	4.6	Multiple Injuries
MTB	5630	CSC	CTRL		37.28	F	B	17	Y		7.1	Hypertrophic cardiomyopathy with aortic dissection
MTB	5656	CSC	CTRL		51.45	F	W	21		Y	N/A	Atherosclerotic cardiovascular disease
MTB	5666	CSC	CTRL		65.55	M	W	25	Y		6.2	Ruptured aortic aneurysm
MTB	5675	CSC	ALS		53.76	M	W	4	Y		7.6	
MTB	5678	CSC	ALS		55.68	M	W	6	Y		5.2	
MTB	5711	CSC	CTRL		50.50	F	B	15		Y	N/A	Complications of lung cancer
MTB	5789	CSC	ALS		71.07	F	W	17	Y		5.5	
MTB	5847	CSC	ALS		53.51	M	W	30	Y		8.1	

GBB: Georgetown Brain Bank
MTB: University of Maryland Brain and Tissue Bank, NIH NeuroBioBank
UCSD: Dept. of Neurosciences, University of California San Diego School of Medicine
JHU: Target ALS Human Multicenter Tissue Core, Johns Hopkins University School of Medicine
CSC / TSC: Cervical / Thoracic Spinal Cord
PMI: Post-mortem interval
RIN: RNA Integrity Number
N/A: not available or Western blot only
